# Human Schlafen 14 Cleavage of Short Double‐Stranded RNAs Underpins its Antiviral Activity

**DOI:** 10.1002/advs.202501727

**Published:** 2025-07-11

**Authors:** Mengyun Li, Dapeng Sun, Wei Hao, Hua Fu, Yueli Zhang, Zhijian Li, Bo Qin, Yumei Wang, Sheng Cui

**Affiliations:** ^1^ NHC Key Laboratory of Systems Biology of Pathogens National Institute of Pathogen Biology Chinese Academy of Medical Sciences and Peking Union Medical College Beijing 100730 P. R. China; ^2^ Institute of Physics Chinese Academy of Sciences Beijing 100190 P. R. China; ^3^ Key Laboratory of Pathogen Infection Prevention and Control (Peking Union Medical College) Ministry of Education Beijing 100730 P. R. China; ^4^ State Key Laboratory of Respiratory Health and Multimorbidity Chinese Academy of Medical Sciences and Peking Union Medical College Beijing 100730 P. R. China

**Keywords:** antiviral mechanisms, biochemical characterization, SLFN14, structures of SLFN14 apoenzyme, structures of SLFN14‐hairpin RNA complex

## Abstract

The Schlafen (SLFN) genes are induced by interferons, underscoring their roles in the immune response and viral replication inhibition. SLFN14, a member of SLFN family, is associated with multiple human diseases; however, neither its functions nor its disease mechanisms are fully understood. Herein, human SLFN14 biochemically is characterized, demonstrating that it specifically cleaves RNAs containing short duplex regions, such as hairpin RNAs and tRNAs, but does not have ATPase or helicase activity. Cryogenic electron microscopy structures of SLFN14 apoenzyme (2.84 Å) and SLFN14‐hairpin RNA complex (2.88 Å) are determined, revealing that SLFN14 assembles into a ring‐like dimer and dimerization is mainly mediated by hydrophobic contacts. Two N‐terminal RNase domains of SLFN14 are organized head‐to‐tail, forming an RNA‐binding groove that can accommodate a 12‐bp hairpin RNA. The hairpin RNA is recognized mainly through phosphate backbone interactions. Further, SLFN14 is shown to inhibits HIV‐1 pseudovirus replication. The anti‐HIV‐1 activity of SLFN14 is via codon‐usage‐dependent translational inhibition and impairment of the programmed ‐1 ribosomal frameshifting, with an efficiency comparable to that of Shiftless. Using tRNA PCR arrays, SLFN14 and SLFN11 are found to decrease both nuclear‐encoded and mitochondrial tRNAs in cells. Together, these results provide novel insights into the function of SLFN14 and its role in HIV‐1 restriction.

## Graphical abstract

SLFN14 is associated with human diseases. SLFN14 is found to cleave RNAs containing short duplexes. The cryo‐EM structures of SLFN14 and SLFN14‐hairpin RNA complex reveal that SLFN14 assembles into a ring‐like dimer; two RNase domains form an RNA‐binding groove accommodating a hairpin RNA. SLFN14 inhibits HIV‐1 replication via codon‐usage‐dependent translational inhibition and the programmed ‐1 ribosomal frameshifting.

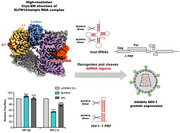

## 1 Introduction

Schlafen (SLFN) proteins are encoded by a family of interferon‐stimulated genes.^[^ ^1^ ^]^ Accumulating evidence has revealed that SLFNs are involved in various cellular functions, including cell proliferation, the immune response, and viral replication restriction.^[^ ^1^, ^2^ ^]^ In recent years, structural characterizations have demonstrated that SLFN proteins share conserved structural architectures despite their diverse roles in the immune responses.^[^ ^3^, ^4^, ^5^, ^6^ ^]^

Many SLFN proteins function as virus restriction factors and biomarkers in cancer therapy.^[^ ^7^, ^8^, ^9^, ^10^ ^]^ For instance, SLFN11 is a potent restriction factor of human immunodeficiency virus 1 (HIV‐1). It targets HIV‐1 protein translation by cleaving type II tRNAs in a codon‐usage‐dependent manner.^[^ ^11^, ^12^ ^]^ In a recent paper, SLFN11 was reported to mediate p53‐independent apoptosis through ribosome stalling on leucine‐encoding UUA codons.^[^ ^13^ ^]^ SLFN11 also functions as a single‐stranded DNA (ssDNA) sensor in the nucleus. The C‐terminal helicase domain of SLFN11 recognizes ssDNA harboring the CGT/A motifs, which leads to cytokine production and cell death through its N‐terminal RNase domain.^[^ ^14^, ^15^ ^]^

SLFN14 is another important member of the SLFN family (group III proteins containing RNase, SWAVDL, and helicase domains) that shares domain organization with SLFN11 and SLFN13.^[^ ^5^, ^6^ ^]^ It is also one of the few SLFN proteins expressed in both humans and mice. However, structural characterization and in‐depth functional analysis of SLFN14 remained lacking. SLFN14 is reported to cleave RNAs and bind ribosomes.^[^ ^16^, ^17^ ^]^ However, there are contradictory results reported previously. Pisareva et al. reported that a short isoform of rabbit SLFN14 (a C‐terminal truncated form, ≈95 kDa) is highly expressed in rabbit reticulocytes. This short isoform associates with ribosomes and exhibits RNase activity, but they did not show results for full‐length rabbit SLFN14 due to difficulty in protein preparation.^[^ ^17^ ^]^ Likewise, full‐length mouse SLFN14 neither has RNase activity nor ribosome‐binding activity, while its ≈65 kDa C‐terminal truncated form resumes both of these activities.^[^ ^17^ ^]^ Pisareva et al. showed that full‐length human SLFN14 protein was inactive in RNase activity and ribosome binding, whereas Fletcher et al. showed that full‐length human SLFN14 is an active RNase.^[^ ^16^ ^]^ Therefore, systematic characterization of the enzymatic activity and structure of SLFN14 is important for understanding its mechanism of action.

SLFN14, like SLFN11, also acts as a restriction factor against several viruses. For example, influenza virus infection induces SLFN14 expression, and SLFN14 overexpression in A549 cells inhibits influenza virus replication and delays transportation of the viral nucleoprotein from the cytoplasm to the nucleus.^[^ ^18^ ^]^ Overexpression of SLFN14 in varicella zoster virus (VZV)‐infected human dermal fibroblasts inhibits expression of viral glycoprotein E (gE) and immediate early protein 62 (IE62), which are essential for VZV replication.^[^ ^18^ ^]^ A recent paper revealed that SLFN14 impairs HIV‐1 protein translation in a codon‐usage‐dependent manner,^[^ ^19^ ^]^ suggesting its functional redundancy with respect to SLFN11.

In addition to its antiviral role, SLFN14 is implicated in human hematologic diseases.^[^ ^20^ ^]^ Fletcher et al. identified monoallelic mutations in the s*lfn14* gene as causative of inherited thrombocytopenia.^[^ ^21^ ^]^ Four missense mutations, K218E, K219N, V220D, and R223W were identified in SLFN14 from thrombocytopenia patients.^[^ ^22^, ^23^ ^]^ Recently, Fabienne et al. suggested that the regulation of ribosomal RNA degradation by SLFN14 is associated with hematologic diseases;^[^ ^24^ ^]^ however, the underlying molecular mechanism remains unclear.

Herein, we carried out a systematic characterization of the enzymatic activities of human SLFN14, revealing that SLFN14 has RNase activity that cleaves RNAs containing double‐strand regions. By determining cryogenic electron microscopy (cryo‐EM) structures of SLFN14 apoenzyme and SLNF14 bound to a short hairpin RNA, we demonstrate that SLFN14 assembles into a ring‐like homodimer, and that an RNA‐binding groove formed by the two SLFN14‐NTD domains can accommodate a short hairpin RNA of ∼12 bp. Further, we provide evidence that SLFN14 inhibits HIV‐1 pseudovirus replication by inhibiting viral protein expression as well as impairing the programmed ‐1 ribosomal frameshifting (‐1 PRF). Using comprehensive human tRNA PCR arrays, we reveal that SLFN14 and SLFN11 decrease both nuclear‐encoded and mitochondrial tRNAs in cells.

## 2 Results and Discussion

### 2.1 SLFN14 Cleaves RNAs Containing Double‐Stranded Regions

**Figure 1:**
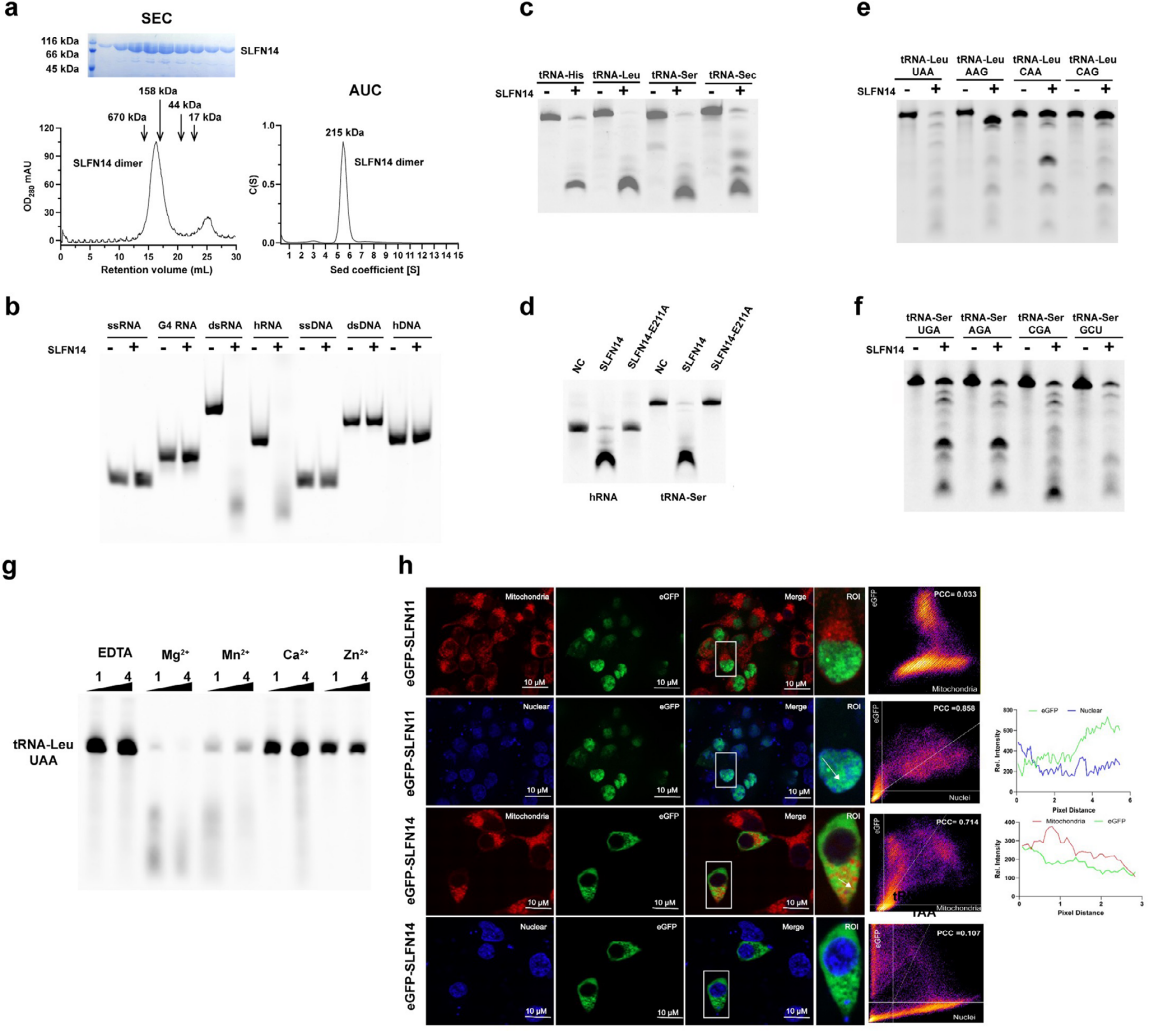
Biochemical characterization of human SLFN14. a) Size‐exclusion chromatography (SEC, left) and analytical ultracentrifugation (AUC, right) analysis of recombinant SLFN14 (strep‐strep‐SUMO tagged) expressed in HEK293F cells; both experiments were carried out in solutions with 150 mm NaCl. Left insert, SDS‐PAGE analysis of the peak fractions. SLFN14 dimer is indicated by an arrow; the molecular mass of gel‐filtration standards is indicated by arrows. b) SLFN14 exhibits different activity on various RNA substrates. Purified SLFN14 (0.5 µm) was incubated with single‐stranded RNA (ssRNA), G‐quadruplex RNA (G4 RNA), double‐stranded RNA (dsRNA), hairpin RNA (hRNA), single‐stranded DNA (ssDNA), double‐stranded DNA (dsDNA), and hairpin DNA (hDNA), respectively. The reaction mixtures were incubated at 37°C for 30 min and resolved using denaturing Urea‐PAGE. c) SLFN14 cleaves all three tRNA types. SLFN14 (0.5 µm) was incubated with type I tRNA (tRNA‐His), type II tRNA (tRNA‐Leu and tRNA‐Ser), and tRNA‐Sec, respectively. The reaction mixtures were incubated at 37°C for 30 min and resolved using denaturing Urea‐PAGE. d) Mutation E211A abrogates RNase activity of SLFN14 on hRNA and tRNA substrates. e) SLFN14 cleaves various tRNA‐Leu isoacceptors with different efficiencies. SLFN14 exhibited higher RNase activity on tRNA‐Leu UAA than on other isoacceptors. f) SLFN14 cleaves various tRNA‐Ser isoacceptors with similar efficiencies. g) Divalent ions are required for the RNase activity of SLFN14. Various ions (concentration 1 and 4µm) that were included in the reactions are indicated on top of the gel; the EDTA control ensured no ions were present in the reactions. Synthetic tRNA‐Leu UAA was used as the substrate. h) Subcellular distribution of SLFN11 and SLFN14. eGFP‐fused SLFN11‐WT or SLFN14‐WT were stably expressed in HEK293T cells. The subcellular distribution of SLFN11 and SLFN14 was visualized using fluorescence microscopy. Mitochondria were labeled with Mito Tracker Deep Red FM (red fluorescence), SLFN14 or SLFN11 with eGFP (green fluorescence), and nuclei with DAPI (blue fluorescence). The white box indicates the region of interest (ROI), and white arrows highlight the plot profiles (leftmost). Colocalization images and corresponding Pearson correlation coefficients (PCC) are shown adjacent to the plot profiles. Scale bars represent 10 µm.

The insolubility of purified SLFN14 has prevented the study of its enzymatic activities. To overcome this obstacle, we engineered a “strep‐strep‐SUMO‐tag” to the N‐terminus of human SLFN14, which yielded the production of a soluble protein (**Figure** **1**a). Both size‐exclusion chromatography (SEC) and analytical ultracentrifugation (AUC) experiments indicated that SLFN14 formed dimers in solutions with physiological salt concentration (150 mm NaCl, Figure 1a). Next, we investigated the substrate specificity of SLFN14 by incubating the enzyme with various nucleic acids (Figure 1b,c). The results showed that SLFN14 efficiently cleaves RNAs containing double‐stranded regions, including double‐stranded (ds) RNA, short hairpin RNA (hRNA), and transfer RNAs (tRNA), but does not cleave single‐stranded (ss) RNA, G4 RNA, and all DNA substrates (Figure 1b,c). Unlike SLFN11, which preferentially cleaves type II tRNA, SLFN14 cleaves all tRNA types (type I, II, and Sec tRNAs, Figure 1c). To rule out nuclease contaminations in the RNase assays, we mutated residue E211 (suspected of being critical to SLFN14 RNase activity) to alanine and confirmed that the E211A mutant was unable to cleave hRNA or tRNA (Figure 1d). Non‐specific cleavage of tRNA by SLFN14 suggests that it may employ a substrate recognition mechanism different from that of SLFN11. Nevertheless, we found that SLFN14 exhibited some level of specificity for tRNA‐Leu isoacceptors (Figure 1e), but not for tRNA‐Ser isoacceptors (Figure 1f).

Further, we tested the requirement of ions in SLFN14‐catalyzed RNA cleavage reactions. Different from SLFN11, which required Mn^2+^ for RNA cleavage reaction,^[^ ^5^ ^]^ we found that both Mg^2+^ and Mn^2+^ could enhance the cleavage activity of SLFN14, and magnesium ion was preferred over manganese ion (Figure 1g).

Together, these biochemical results indicated that both SLFN14 and SLFN11 cleave tRNAs, although their RNA substrate specificity and coenzyme requirement are district. Whereas SLFN11 specifically cleaves type II tRNAs, SLFN14 cleaves a broader range of substrates harboring double‐strand RNA regions. To understand the functional redundancy of SLFN14 and SLFN11, we investigated the subcellular distribution of the two proteins using fluorescence microscopy (Figure 1h). The results showed that, when overexpressed in HEK293T cells, the enhanced green fluorescent protein (eGFP) tagged SLFN11 proteins were primarily found in the nucleus, whereas most eGFP‐SLFN14 proteins were found in the cytoplasm (Figure 1h). The results suggest that SLFN14 and SLFN11 act in separate subcellular compartments.

### 2.2 Apo Structure of SLFN14

**Figure 2:**
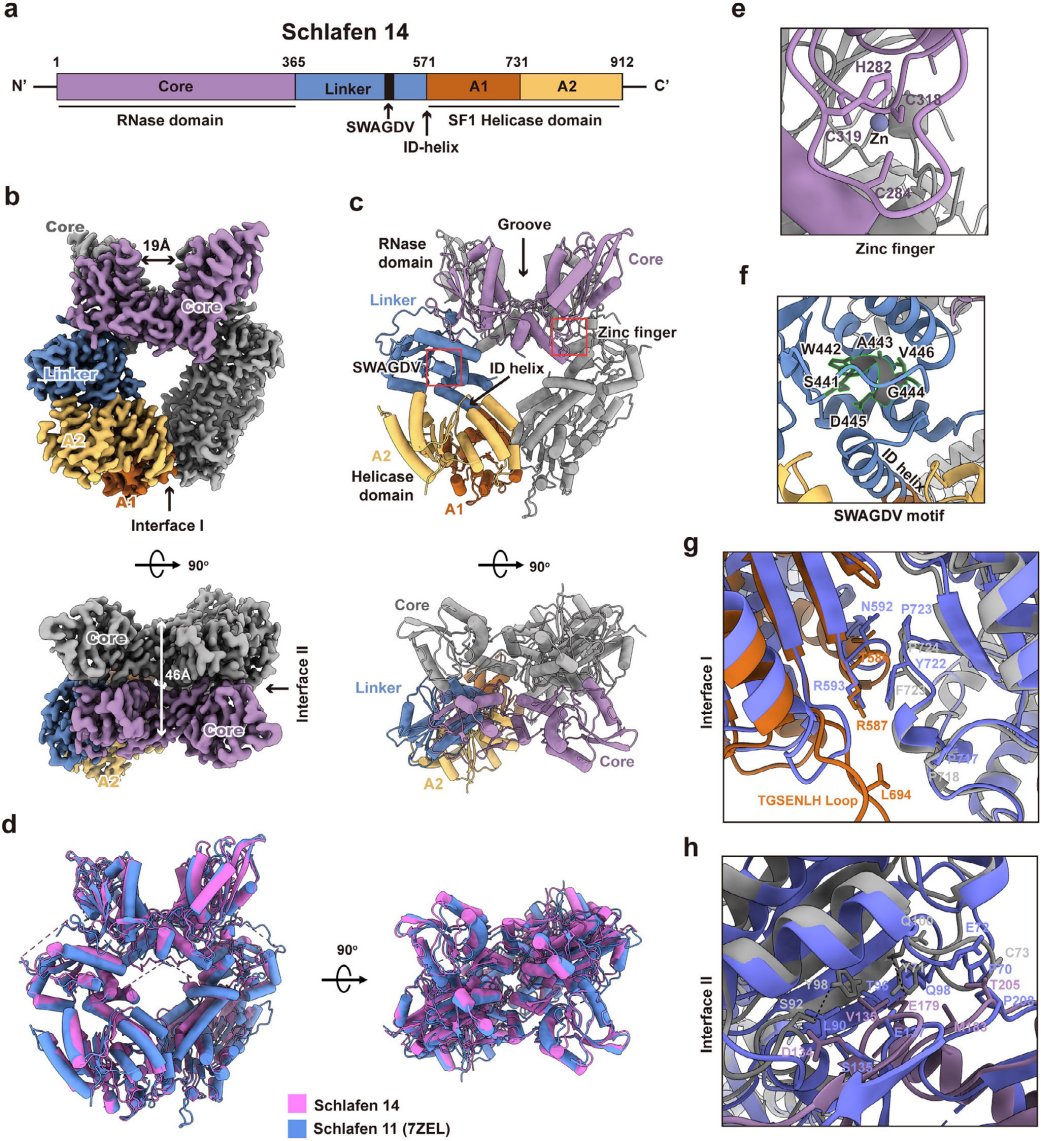
Cryo‐EM structure of human SLFN14 apoenzyme. a) Schematics of human SLFN14 domain organization. The core domain is colored purple, the Linker domain is colored blue, the SF1 helicase RecA‐like domain 1 (A1) is colored orange, and the RecA‐like domain 2 (A2) is colored yellow. b) Top, side view of cryo‐EM density map of the ring‐like SLFN14 homodimer. One protomer is colored by domains using the same scheme as in panel a, the other protomer is colored gray. A groove formed by two core domains exhibits a width of ≈19Å. Bottom, top view of the homodimer, the length of the groove is ≈46Å. Dimer interface I and II between core domains and helicase domains are indicated by arrows. c) Ribbon model of SLFN14 homodimer; the zinc finger and the SWAGDV motif are highlighted by red boxes. d) Left, side view of structure superimposition of human SLFN14 (magenta) with human SLFN11 (blue, PDB, 7ZEL) structures. Right, top view. e) Magnified view of the zinc finger. f) Magnified view of the SWAGDV motif. g,h) Superimposition of human SLFN14 and human SLFN11 structures zoomed in at the dimer interface I, panel g, and interface II, panel h. Key residues involved in dimerization are shown with stick models and labeled; human SLFN14 is colored by domains with the same scheme as in panels a & b; a human SLFN14 unique loop, the TGSENLH loop, is indicated.

Despite the similar domain structure between SLFN14 and other group III SLFN proteins, such as SLFN11, SLFN13, and SLFN5 (**Figures** **2**a, and S1, Supporting Information), we found that the RNA substrate specificity of SLFN14 was distinct from that of other SLFN family members. Therefore, we characterized SLFN14 structurally to determine the mechanism responsible for this specificity. During purification of recombinant proteins, we noticed that SLFN14 dimers were more stable than SLFN11 dimers. In physiological ionic strength (≈150 mm NaCl) solutions, SLFN14 dimers were stable (Figure 1a), whereas SLFN11 dimers could break down into monomers.^[^ ^5^ ^]^

To reveal the structural details underpinning the function of SLFN14, we determined the cryo‐EM structures of the full‐length human SLFN14 apoenzyme. Because wild‐type (WT) SLFN14 protein was not suitable for single‐particle analysis, primarily because it strongly aggregated on the cryo‐EM grid, we introduced four mutations, i.e., E211A, C365S, C775S, and C808S into SLFN14 (denoted SLFN14^mut^) to circumvent this problem. The three C‐to‐S mutations were introduced to disrupt intermolecular disulfate bonds, which are known to cause protein aggregation. SLFN14^mut^ was more stable and soluble, which allowed us to perform 3D reconstruction of the sample using ≈0.96 million particles, yielding an interpretable EM density map with an overall resolution of 2.84 Å (Figures S3a and S4, Supporting Information) that was suitable for model building (Figure 2b; Table S1, Supporting Information).

The cryo‐EM structure of apo SLFN14^mut^ features a dimer with an overall fold similar to that of the SLFN11 dimer (Figure 2b–d). Specifically, two intertwined SLFN14 monomers are organized with C2 symmetry, forming a ring‐like architecture (Figure 2b,c). This ring‐like architecture of SLFN14 is composed of two copies of SWAGDV motif and helicase domain forming the body of the ring and two copies of N‐terminal RNase domains organized head‐to‐tail, forming a central RNA binding groove on top of the ring (Figure 2b,c). The N‐terminal RNase domain (1‐365 aa, also known as the core domain) of SLFN14 has a fold similar to that of other SLFN cores reported previously.^[^ ^5^, ^6^, ^11^ ^]^ Two openings in the SLFN14 RNase domains are combined head‐to‐tail, forming an elongated groove with a length of ≈46Å and a width of ≈19Å (Figure 2b,c). The size of the groove is sufficient to accommodate an RNA duplex. The SLFN14 RNase domain is followed by a linker domain (366‐571 aa) and a superfamily I helicase domain (572‐912 aa, Figure 2b,c). The loop bridging the RNase and linker domains is disordered in the EM density map, whereas the long helix (558‐584 aa) connecting the linker and helicase domains is well ordered (Figure 2c). We term this helix ‘interdomain helix’ (ID‐helix) because it is structurally related to the ID‐helix identified in SLFN11 previously.^[^ ^5^ ^]^

SLFN14 harbors a zinc‐finger module located at the C‐lobe of the RNase domain, where a zinc ion is coordinated by residues C318, C319, H282, and C284 (Figure 2e). The SLFN family conserved ‘SWAVDL’ motif is changed to ‘SWAGDV’ in SLFN14 and is located in the linker domain. Despite the difference in sequence, it still adopts the helical conformation of SWAVDL motif found in other SLFN protein structures (Figure 2f).

We conducted a Dali search (http://ekhidna2.biocenter.helsinki.fi/dali)^[^ ^25^ ^]^ to identify SLFN14 homologs in the Protein Data Bank. SLFN11 apoenzyme (PDB, 7ZEL) was the top hit with Z‐score 42.2, rmsd 2.2Å, and aligned 827 C𝑎 atoms (Figure 2d). Despite overall structural similarity, the dimerization interfaces are different. Using software PISA (Proteins, Interfaces, Structures and Assemblies), we calculated the dimer interfacial area for SLFN14 as 2807Å^2^ (ΔG = −17.9 kcal mol^−1^), which is larger than that of SLFN11 dimer interfacial area, 1848Å^2^ (ΔG = −1.1 kcal mol^−1^). This suggests that the SLFN14 forms stable dimers in solution. As shown in Figure S2a–d (Supporting Information), both SEC and AUC experiments showed that SLFN14 formed primarily dimers in buffers containing either low salt (40mm NaCl) or physiological salt (150mm NaCl).

The cryo‐EM structure revealed two dimer interfaces (interface I and II, Figure 2g,h) in SLFN14. Dimer interface I is located between the helicase domains (Figure 2g), which is maintained primarily by the hydrophobic interactions between T586‐P724, R587‐F723 (π‐staking), and L694‐P718. At the interface I of SLFN11 dimer, although interactions between N592‐P723 and R593‐Y722 are conserved,^[^ ^5^ ^]^ hydrophobic interaction involving L694 is unavailable in SLFN11 (Figure 2g; Table S2, Supporting Information). Residue L694 is located on a loop (denoted the TGESENLH‐Loop, Figure 2g) unique in SLFN14. The topologically equivalent loop in SLFN11 is much shorter, thus, it cannot reach the other monomer (Figure 2g; Table S2, Supporting Information). The dimer interface II of SLFN14 is between two N‐terminal RNase domains (Figure 2h). It is centered by hydrophobic interactions between the hydrophobic patch Y71‐C73‐Y98 on one protomer and the hydrophobic patch V135‐M183‐T205 on the other protomer; this hydrophobic contact is further strengthened by two polar interactions between S92‐D134 and Q100‐E179. Again, we found that a polar contact Q100‐E179 is unique in SLFN14, and unavailable at SLFN11 dimer interface II (Figure 2g; Table S2, Supporting Information). Together, the dimer interface analysis suggests that SLFN14 may function exclusively as dimers in solution.

### 2.3 Structure of the SLFN14‐Hairpin RNA Complex

**Figure 3:**
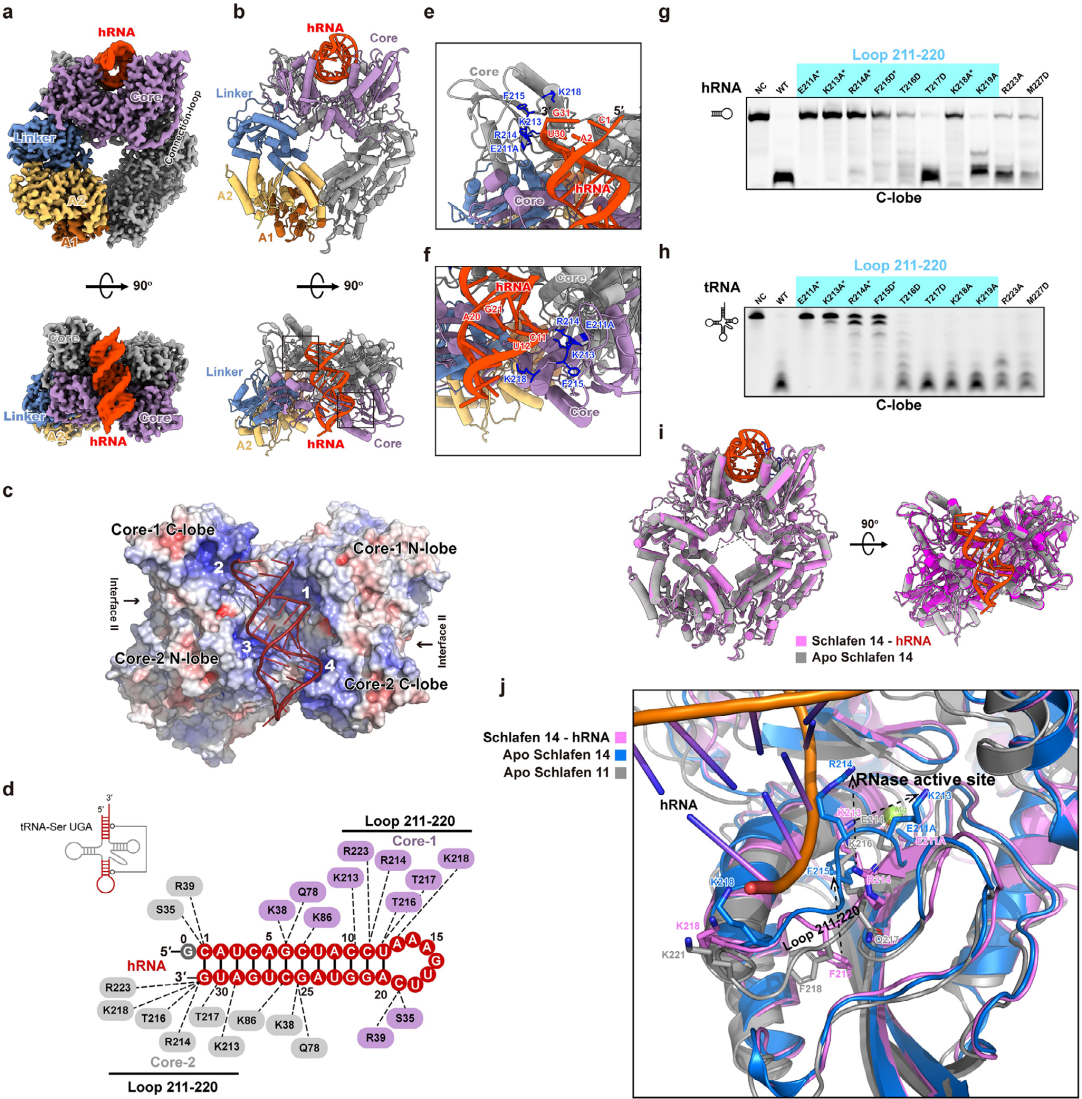
Cryo‐EM structure of human SLFN14 complexed by short hairpin RNA. a) Top, side view of cryo‐EM density map of SLFN14‐short hairpin RNA (hRNA) complex. One protomer is colored by domains using the same scheme as in Figure 2a, the other is colored gray. The hRNA accommodating the central groove formed by core domains is colored red. Bottom, top view of the density map. b) Ribbon model of SLFN14‐hRNA complex; key RNA binding sites are indicated by boxes and magnified in panels e and f. c) Surface electrostatic potential plot of SLFN14 RNA binding groove occupied by hRNA (red ribbon). Four positively charged zones 1–4 within the RNA binding groove are indicated. d) Diagram of SLFN14‐hRNA interactions; residues participating in the interaction are labeled and indicated by dashed lines. Top left insert, design of hRNA, red regions of tRNA‐Ser‐UGA are combined to yield hRNA. e,f) Detailed structure of SLFN14‐RNA interaction at two active sites. g,h) Mutagenesis studies. RNase assays were performed for wild‐type (WT) SLFNF14 and a selection of mutants (indicated on top of the gels). Panel g) hRNA was used as the substrates. Panel h) tRNA‐Leu was used as the substrate. i) Structure superimposition of apo and hRNA bound SLFN14. j) Zoom in of SLFN14 active site superimposed with SLFN11; catalytically important residues are shown with stick model and labelled.

We have shown that SLFN14 specifically cleaves tRNAs, dsRNA, and hRNA efficiently; thus, the short double strand RNA region that characterizes these substrates is probably the structural feature recognized by SLFN14. To test this hypothesis, we incubated dsRNA, hRNA, or tRNA substrates with SLFN14^mut^ as substrates and performed single‐particle analysis. Only 3D reconstruction of SLFN14‐hRNA complex particles (0.34 million particles) yielded a clear EM density map (2.88 Å resolution) of the bound hairpin RNA molecules and allowed model building (**Figure** **3**a,b; Figures S3b and S5, Supporting Information).

The hRNA was designed by fusing the acceptor stem with the anticodon stem loop of human tRNA‐Ser UGA, yielding a 32‐mer RNA strand that could fold into an RNA structure with a 12‐bp stem and a 7‐nt loop (Figure 3d). We located 31 out of 32 nucleotides into the final EM density map. Intriguingly, the addition of hRNA improved the overall resolution of the entire SLFN14 dimer. For example, the flexible Connection‐loop connecting the N‐ and C‐lobes of the SLFN14 RNase domain was disordered in the density map of the SLFN14 apoenzyme structure but was resolved clearly in the density map of the hRNA‐bound SLFN14 structure (Figures 2b and 3a). This result suggests that hRNA binding stabilizes the folding of SLFN14.

The SLFN14‐hRNA complex maintains the ring‐like fold similar to that of the apoenzyme. The hRNA accommodates a central RNA binding groove formed by two N‐terminal RNase domains of SLFN14 (Figure 3a–c). The bound hRNA adopts a typical A‐form RNA duplex conformation. Surface electrostatic plot revealed four positively charged zones within the RNA binding groove of the SLFN14‐hRNA complex structure (Figure 3c). Zones 1 & 3 are located on the RNase N‐lobes involving five residues (S35, K38, R39, Q78, and K86) in each lobe. Zones 2 & 4 are located on the RNase C‐lobes involving six residues (K213, R214, T216, T217, K218, and R223) in each lobe. All residues in the four zones recognized the hRNA through the phosphate backbone contacts, implying that the RNA‐binding mode of SLFN14 is probably structure‐specific rather than sequence‐specific (Figure 3d).

Mutagenesis study of amino acids in the RNA binding site of C‐lobe (zone 2 & 4) identified E211, K213, R214, and K218 as essential residues for RNA cleavage (Figure 3g,h). These residues are clustered in a short loop (residues 211–220), hence denoted Loop 211–220. Superimposition of the apoenzyme and the RNA‐bound SLFN14 structures revealed that Loop 211–220 underwent a large conformational rearrangement upon RNA binding (Figure 3i,j), suggesting that it plays an important role in RNA substrate recognition. The locations of the catalytically important residues in the structure of SLNF14‐hRNA complex (Figure 3e,f) revealed two RNase active sites in the RNA binding groove, in zones 2 & 4, which were separated by a distance of ≈12‐bp on the hRNA.

Residue E211 is highly conserved in SLFN family members. The counterpart of SLFN14 E211 is SLFN11 E214, which coordinates a metal ion in its RNase active site.^[^ ^5^ ^]^ Because E211 was mutated to alanine in SLFN14^mut^, we could not observe E211‐hRNA interaction or metal coordination in the structures. F215 points away from hRNA, suggesting that it does not directly interact with RNA. However, the results showed that the mutation F215A impaired the RNase activity of SLFN14, suggesting that it is involved in maintaining the active conformation of Loop 211–220 (Figure 3e,f,j).

Superimposition of SLFN14 apoenzyme, RNA‐bound SLNF14, and SLFN11 apoenzyme structures showed that the folding of the RNase active site is similar in these structures (Figure 3j). The catalytically important residues E211, K213, F215, and K218 identified by mutagenesis in SLFN14 are conserved among SLFN proteins, and R214 is not conserved (Figure S1, Supporting Information). In the hRNA bound SLFN14 structure, all catalytically important residues (except E211A) adopted conformations similar to those in the active site of SLFN11, suggesting a conformation optimal for RNase activity (Figure 3j). By contrast, in the SLFN14 apoenzyme structure, K213, R214, and F215 on Loop 211–220 are shifted away from the active site (Figure 3j), suggesting the SLFN14 apoenzyme structure may represent the RNase inactive state of the enzyme.

### 2.4 Mechanism of SLFN14‐Catalyzed RNA Cleavage

**Figure 4:**
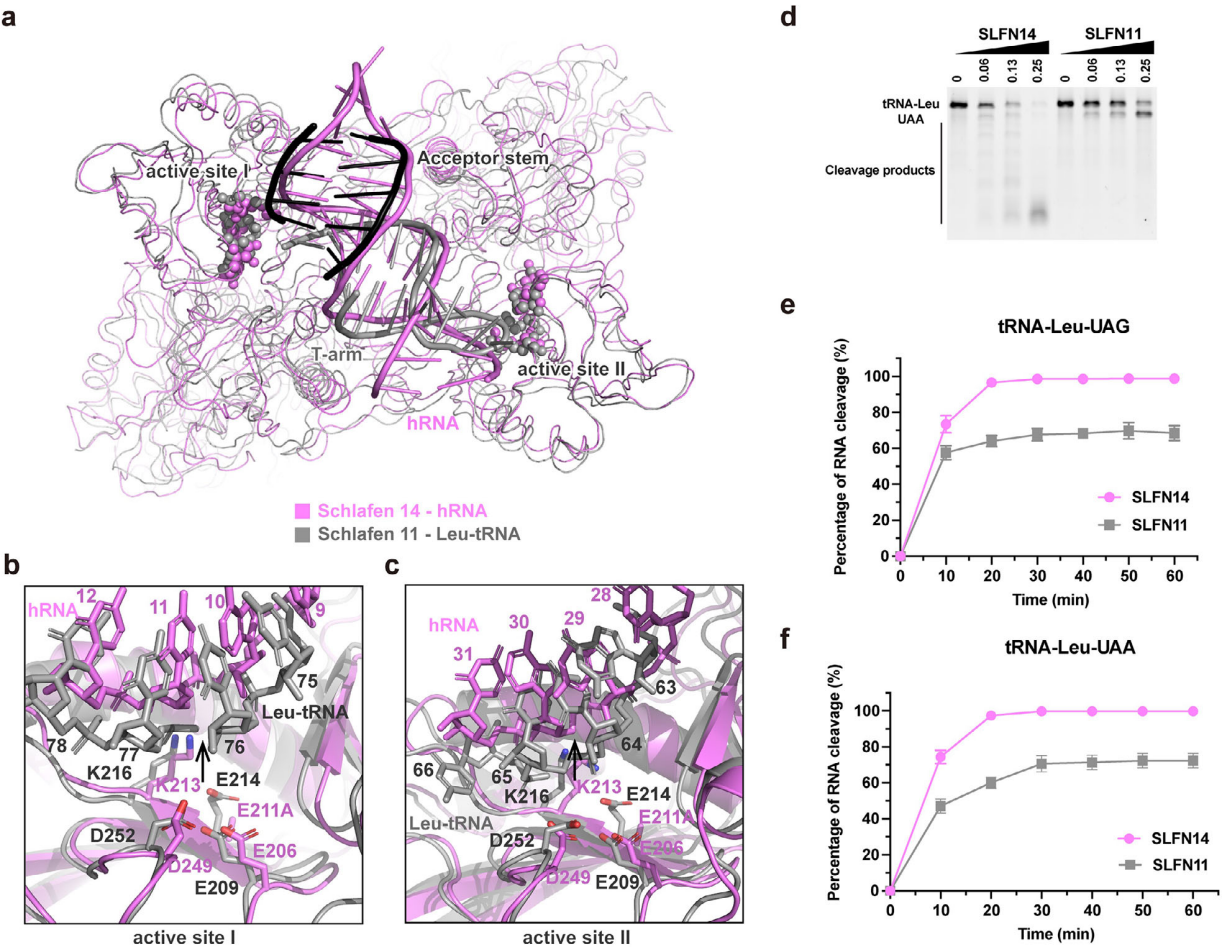
Comparison of the RNase active site and cleavage activity between SLFN14 and SLFN11. a) Superimposition of SLFN14‐hRNA structure (magenta) with SLFN11‐tRNA‐Leu structure (PDB: 9GMX, in which only the tRNA Acceptor stem, colored black, and T‐arm, colored gray, are shown). The protein portions of the complexes are shown with ribbon models, the RNA portions are shown with cartoon models. Residues at the RNase active site are shown with spheres. b) Zoom‐in view of the structure superimposition at the RNase active site I; key residues are shown with stick models and labeled. c) Zoom‐in view of the structure superimposition at the RNase active site II; key residues are shown with stick models and labeled. d) Cleaving of tRNA‐Leu‐UAA substrate by SLFN14 and SLFN11; cleavage products were resolved by denaturing Urea‐PAGE. Enzyme concentration is indicated on top of the gel; the concentration of tRNA substrate was 0.25 µm in all reactions. e,f) RNase activity comparison between SLFN14 (magenta symbols and lines) and SLFN11 (gray symbols and lines) in cleaving tRNA‐Leu UGA **e** and tRNA‐Leu UAA **f** over an incubation time of 60 min. Enzyme concentration and tRNA substrate concentration in all reactions were fixed at 0.25 µm. Values at each data point are presented as mean ± SD (*n* = 3).

During submission of this paper, Kugler and colleagues published high‐resolution cryo‐EM structures of SLFN11 bound by different tRNAs, providing details for RNA recognition and cleavage by SLFN11.^[^ ^26^ ^]^ Although SLFN14 and SLFN11 share the overall architecture, domain organization, and particularly the folding of the N‐terminal core domain, differences in the RNA recognition and cleavage mechanism remain to be understood. To this end, we compared the cryo‐EM structure of SLFN14‐hRNA with that of SLFN11‐Leu‐tRNA (PDB: 9GMX) and found that, although the central RNA binding groove of SLFN14 and SLFN11 showed similar shape and size, the underlying RNA recognition mechanisms were different (**Figure** **4**a–c). Because a catalytic SLFN14 mutant was used for determining our EM structures, in which residue E211 (equivalent to SLFN11 E214 that coordinates Mn^2+^ ions) was replaced by alanine, E211A mutation could abrogate the ability of ion coordination. This is consistent with the observation that we did not find densities for ions in our cryo‐EM structure (Figure S6, Supporting Information). Thus, the structure of SLFN14‐hRNA may represent a pre‐cleavage state of the enzyme (Figure 4b,c).

One of the key differences in RNA recognition between SLFN14 and SLFN11 is that, while SLFN14‐NTD recognizes a quasi‐symmetric A‐form RNA helix without a sequence readout mechanism, SLFN11 recognizes asymmetric features of the tRNAs, including specific sequence‐readout in the acceptor stem and variable arm.^[^ ^26^ ^]^ As shown in Figure 4a–c, the proficient active site I and the deficient active site II of SLFN11 recognized different structural features: the double‐strand region in the acceptor stem is recognized by active site I, and the loop region in the T‐arm is recognized by active site II. By contrast, active sites I & II in SLFN14 both recognized the double‐strand regions in hRNA without sequence‐readout (Figure 4a–c). Therefore, the cryo‐EM structures of SLFN14‐hRNA indicate a similar RNA binding mode at both active sites, which is structurally indistinguishable (Figure 4a–c).

Given that SLFN14 is an active RNase (Figure 1b–g) harboring two structurally indistinguishable RNase active sites (Figure 4a–c), it is possible that both sites in SLFN14 are proficient in RNA cleavage, which may explain that the SLFN14‐mediated cleavage can yield shorter products. To test this hypothesis, we directly compared the RNase activity of SLFN14 and SLFN11 in cleaving the tRNA‐Leu UAA (Figure 4d). Consistent with the results reported previously,^[^ ^5^, ^26^ ^]^ SLFN11 cleaved at ≈10 nucleotides from the 3’‐ end of the tRNA to produce a band slightly lower than the substrate (Figure 4d). By contrast, SLFN14 cleaved the same tRNA down to many short fragments, suggesting that SLFN14 cleaved the tRNA at multiple sites.

Next, we compared the cleavage of tRNA‐Leu‐UAG and tRNA‐Leu‐UAA by SLFN11 and SLFN14 over a time course of 60 min (Figure 4e,f); the concentration of enzyme and RNA in all reactions was fixed to 0.25 µm. SLFN14 efficiently cleaved ≈100% of the tRNA in 20 min, whereas SLFN11 only achieved ≈70% the cleavage after 60 min (Figure 4e,f), which agreed with the results reported previously.^[^ ^26^ ^]^ These results suggest that, unlike SLFN11‐catalyzed cleavage, after which the product remains bound to the enzyme, SLFN14 cleaves the substrate into shorter fragments and releases the products after the reaction.

Collectively, our results suggest different RNA recognition and cleavage mechanisms of SLFN14 from those of SLFN11. SLFN14 may adopt an induced‐fit mechanism for RNA substrate recognition, which involves RNA binding‐induced conformational change of the Loop 211–220. Binding of a hRNA (∼12 bp) induces a conformational transition of its RNase active site from an inactive to active state.

### 2.5 The ATPase/Helicase Active Site and ssDNA Binding Sites of SLFN14

One unusual feature of SLFN14 is the presence of non‐canonical residues at conserved positions in the Walker A motif of its SF1 helicase domain, suggesting that SLFN14 is inactive in ATPase and helicase activity. The Walker A motif “PGSGKT” shared by other SLFN proteins is changed to “PG *VR* KT” in SLFN14. Nevertheless, the structure of SLFN14 shows that the PG *VR* KT motif still adopts the P‐loop conformation of the bona fide Walker A motif (Figure S7a, Supporting Information). Additionally, the ID‐helix in SLFN14 blocks access to the ATP‐binding pocket, as observed previously for the autoinhibited ATPase conformation observed in SLFN11 (Figure S7a, Supporting Information).

Next, we superimposed the SLFN11‐ssDNA complex (PBD, 7ZES) and SLFN14 apoenzyme structure to analyze whether DNA binding groove exists in SLFN14 (Figure S7b, Supporting Information). Although overall folding of the putative SLFN14 DNA binding groove is similar to that of SLFN11, many ssDNA binding residues are not conserved (Figure S7b, Supporting Information). Particularly, four positively charged DNA binding residues of SLFN11 R656, R674, R855, and R856 are replaced by noncharged residues in SLFN14, namely Q650, C668, Q866, and Q867 (Figure S7b, Supporting Information). To understand whether different residues at the DNA binding groove indeed affect ssDNA binding capability, we compared binding of SLFN14 and SLFN11 with a ssDNA strand; this was the same 50‐nt oligo used for characterizing DNA binding of SLFN11 previously.^[^ ^5^ ^]^

First, we performed electrophoretic mobility shift assays (EMSA), which showed that both SLFN14 and SLFN11 bound ssDNA with similar affinity (Figure S7c, Supporting Information). Nevertheless, heavy aggregations found in the wells of native‐PAGE indicated that this method might not be optimal (Figure S7c, Supporting Information); therefore, we used biolayer interferometry (BLI) to gain further details in binding kinetics (Figure S7d, Supporting Information). We labeled biotinylated ssDNA probes on streptavidin biosensors for binding with SLFN proteins in solutions of different protein concentrations (Figure S7d, Supporting Information). Steady‐state evaluations of the BLI titrations showed that binding affinity of ssDNA for SLFN14 is ∼ 4‐folds higher than that of SLFN11; the *K_D_* values were 9 and 37 nm, respectively. However, in the dissociation steps, SLFN11 showed remarkably slower off‐rate; the k_dis_ values for SLFN11 and SLFN14 were 4.8*10^−4^/s and 1.3*10^−3^/s, respectively. These results demonstrate that while the SLFN11‐ssDNA interaction is a ‘fast‐on and slow‐off’ process, the SLFN14‐ssDNA interaction is a ‘fast‐on and fast‐off’ process, which suggests SLFN11 tends to stay binding with ssDNA.

To test whether SLFN14 is deficient in ATPase and helicase activities, we performed ATPase and RNA duplex unwinding experiments (Figure S8, Supporting Information). MERS‐CoV nsp13, an active SF1 RNA helicase,^[^ ^27^ ^]^ was used as the positive control. Whereas MERS nsp13 efficiently hydrolyzed ATP and unwound an 18‐bp partial RNA duplex substrate with a 5’ single strand overhang, SLFN14‐E211A (an RNase catalytic mutant that still harbors WT C‐terminal helicase domain) did not hydrolyze ATP even in the presence of nucleic acids (Figure S8a, Supporting Information). SLFN14‐E211A also could not unwind the partial RNA duplex substrate, indicating that SLFN14 is inactive as a helicase (Figure S8b, Supporting Information).

### 2.6 SLFN14 Restricts HIV‐1 Pseudovirus Replication

**Figure 5:**
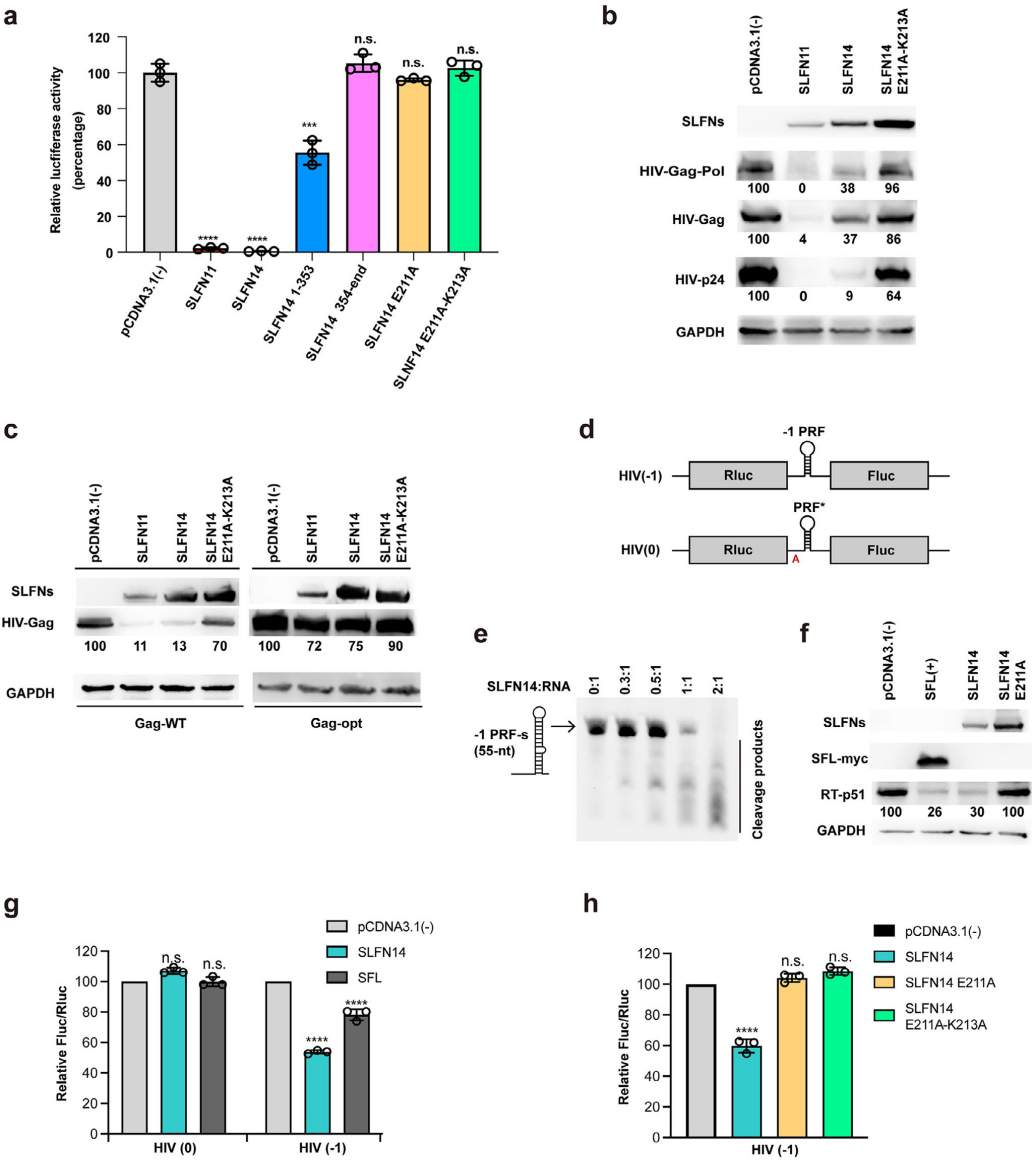
SLFN14 inhibits HIV‐1 pseudovirus replication via codon‐usage‐based suppression of viral protein translation and programmed ‐1 ribosomal frameshifting. a) SLFN14 inhibits VSV‐G pseudotyped HIV‐1 production. HEK293T cells were co‐transfected with pNL‐4‐3. luc.R‐. E ‐, pCMV‐VSV‐G, and indicated plasmid expressing SLFN11 and SLFN14 variants (cloned to pCDNA3.1 vectors). Levels of infectious viruses in the supernatants were determined by reinfecting fresh HEK293T cells, followed by measuring luciferase activity. Data, representing three independent experiments, are presented as means ± SD (*n* = 3 per group). Statistical analysis was performed using one‐way ANOVA. ^***^ *P*<0.001 vs. pCDNA3.1. ^****^ *P*<0.0001 vs. pCDNA3.1. b) SLFN14 inhibits HIV‐1 protein expression in a codon‐usage‐dependent manner. HEK293T cells were co‐transfected with SLFN11, SLFN14 or SLFN14 E211A‐K213A, along with pNL‐4‐3.luc.R‐. E‐. At 48 h post‐transfection, cell lysates were immunoblotted for V5 (C‐terminal tag of SLFNs), Gag‐Pol, Gag, p24, and GAPDH using an antibody. The number under the immunoblotting band is the quantification of band density. c) HEK293T cells were co‐transfected with SLFN11, SLFN14 or an RNase‐deficient mutant SLFN14 E211A‐K213A, along with plasmids encoding either the wild‐type gag gene sequence (Gag‐WT, left panel) or codon‐optimized gag sequence (Gag‐opt, right panel). At 48 h post‐transfection, cell lysates were immunoblotted for V5, Gag, and GAPDH using an antibody. d) Schematic representation of the dual‐luciferase reporter assay. The HIV(‐1) contains the wild‐type ‐1 PRF slippery sequence from HIV‐1. An additional adenine was inserted after the slippery sequence in the HIV (0) as a negative control for ‐1 PRF. e.Cleavage of ‐1 PRF‐s (a 55‐nt RNA spanning core regions of HIV‐1 ‐1 PRF sequence) by SLFN14; the RNA concentration was fixed to 0.25 µm, and the enzyme‐to‐RNA ratio is listed on top of the gel. f) SLFN14 inhibits the expression of HIV‐1 reverse transcriptase. HEK293T cells were co‐transfected with the indicated plasmids along with pNL‐4‐3.luc.R‐. E‐. 48 h post‐transfection, cell lysates were immunoblotted for V5 (the C‐terminal tag of SLFN14 or its mutant E211A), myc (the C‐terminal tag of SFL), HIV‐1 RT (RT‐p51), and GAPDH using respective antibodies. The numbers under the immunoblotting bands of RT‐p51 are the band density quantification. g,h) SLFN14 inhibits the ‐1 PRF of HIV‐1. HEK293T cells were co‐transfected with a dual‐luciferase reporter and either wild‐type SLFN14 or mutant constructs, with pCDNA3.1 and SFL as controls. Luciferase activities (Fluc/Rluc ratio) were measured 32 h post‐transfection to assess ‐1PRF efficiency, normalized to pCDNA3.1 (set at 100%). Data are presented as means ± SD from three replicates (*n* = 3 per group,^****^ *P* < 0.0001 vs. pCDNA3.1).

Several SLFN proteins including SLFN11, 12, and 13 were found to restrict HIV‐1 replication through codon‐usage‐based translational suppression.^[^ ^6^, ^12^, ^28^ ^]^ To investigate whether SLFN14 has similar activity, we performed HIV‐1 pseudovirus infection assays. We transfected pNL4‐3.luc.R‐.E‐ (HIV‐1 backbone plasmid) and pCMV‐VSV‐G along with vectors expressing WT SLFN11, WT‐SLFN14, SLFN14‐N (residues 1–353), SLFN14‐C (residues 354–912), SLFN14 E211A (RNase catalytic mutant) or SLFN14 E211A‐K213A (double RNase catalytic mutation that abrogates both RNA cleavage and RNA binding) to HEK293T cells. As shown in **Figure** **5**a, WT‐SLFN11 and WT‐SLFN14 greatly reduced HIV‐1 pseudovirus replication to the background level, indicating that SLFN14 restricts HIV‐1 replication similarly to SLFN11. It has been previously shown that, endogenous expression level of SLFN14 and SLFN11 in HEK293T cells is very low;^[^ ^12^ ^]^ therefore, the inhibition of HIV‐1 pseudovirus replication observed in the above experiments was most likely attributed to the ectopically expressed SLFN proteins.

SLFN14‐N harboring only the RNase domain that retained ≈50% of inhibitory activity against HIV‐1 pseudovirus, whereas SLFN14‐C harboring the Linker and helicase domains had no inhibitory activity (Figure 5a). This result indicates that the N‐terminal RNase domain of SLFN14 is critical for anti‐HIV‐1 activity. Nevertheless, the C‐terminal portion of SLFN14 still contributed to anti‐HIV‐1 activity because the anti‐HIV‐1 activity of SLFN14‐N was ≈50% lower than that of full‐length SLFN14 (Figure 5a). The RNase catalytic mutant SLFN14‐E211A lost nearly all anti‐HIV‐1 activities, and the activity of the double mutant SLFN14 E211A‐K213A was even more severely reduced (Figure 5a). Therefore, we were confident in using this double mutant as the negative control in the following experiments.

### 2.7 Mechanisms Underpinning Anti‐HIV‐1 Activity

To investigate whether the anti‐HIV‐1 activity of SLFN14 is due to its ability to inhibit viral protein translation, we co‐transfected plasmid pNL4‐3.luc.R‐. E‐ with plasmids encoding SLFN11, SLFN14 or SLFN14 E211A‐K213A. SLFN11 inhibited expression of all viral proteins, including HIV‐1 gag‐pol precursor, HIV‐1 gag, and HIV‐1 p24, while SLFN14 preferentially inhibited HIV‐1 p24 expression; the expression of HIV‐1 p24 was ∼ 4‐fold lower than that of the expression of other HIV‐1 precursor proteins in the presence of SLFN14 (Figure 5b). The double mutant SLFN14 E211A‐K213A had a negligible effect on the expression of all of these HIV‐1 proteins expression, including p24.

To determine whether the SLFN14‐mediated inhibition of HIV‐1 protein translation was governed by codon usage, we optimized the codon usage of the HIV‐1 gag gene. As shown in Figure 5c, translation of the original HIV‐1 gag gene (CAI = 0.64) was greatly suppressed by both SLFN11 and SLNF14. By contrast, translation of the codon‐optimized HIV‐1 gag gene (CAI = 0.90) was unaffected by either SLFN11 or SLNF14 (Figure 5c). These results suggest that SLNF14 inhibits HIV‐1 protein translation in a codon‐ usage‐dependent manner.

We reasoned that differences in SLFN14‐mediated translational inhibition of various HIV‐1 proteins could be due to additional mechanisms. It is known that Shiftless (SFL) can inhibit the programmed ‐1 ribosomal frameshifting (‐1 PRF) during HIV‐1 Gag‐Pol translation.^[^ ^29^ ^]^ Inhibition of ‐1 PRF results in lowered expression of HIV‐1 pol and proteinase, which could eventually hinder proteolytic processing of HIV‐1 gag to yield p24. To test whether SLFN14 can also inhibit ‐1 PRF, we employed a previously reported dual‐luciferase reporter pDual‐HIV (‐1) system,^[^ ^29^ ^]^ in which ‐1 PRF signals (the slippery sequence and downstream stimulatory hairpin) of HIV‐1 gag‐pol are inserted between Renilla luciferase (Rluc) and firefly luciferase (Fluc) coding sequences (Figure 5d). A control reporter pDual‐HIV (0) contained a mutation in the slippery sequence that assigned Fluc and Rluc to the same reading frame. As shown in Figure 5g, SLFN14 had even higher inhibitory activity against ‐1 PRF than the SFL, indicating that SLFN14 could indeed inhibit ‐1 PRF. Further, we found that the SLFN14‐mediated ‐1 PRF inhibition is dependent on SLFN14 RNase activity, because the catalytic mutants SLFN14 E211A and SLFN14 E211A‐K213A lost the inhibitory activity against ‐1 PRF (Figure 5h).

Further, we directly analyze the expression of the viral reverse transcriptase (RT or p51), because RT expression is dependent on ‐1 PRF of HIV‐1 Gag‐Pol. As shown in Figure 5f, when co‐transfected plasmids encoding SFL, WT‐SLFN14 or a SLFN14 catalytic mutant E211A along with the pNL‐4‐3.luc.R‐. E‐ plasmid, we found that SLFN14 could inhibit the expression of HIV‐1 RT with an efficacy similar to that of SFL, but the SLFN14 E211A mutant lost the ability of RT expression inhibition, confirming the SLFN14‐mediated ‐1 PRF inhibition was dependent on its RNase activity (Figure 5f).

Of note, the stimulatory hairpin element downstream of the slippery sequence of HIV‐1 gag‐pol is a 11‐bp hairpin (Figure 5d),^[^ ^29^ ^]^ which is not very different in size from that of the 12‐bp hRNA that is accommodated by the RNA binding groove of SLFN14. It is thus possible that, SLFN14 inhibits ‐1 PRF by binding and/or cleaving the stimulatory hairpin. To support this hypothesis, we synthesized a 55‐nt RNA strand harboring the core region of HIV‐1 ‐1 PRF sequence (denoted ‐1 PRF‐s, Figure 5e), which contained the slippery sequence and the downstream stimulatory hairpin. As anticipated, SLFN14 efficiently cleaved ‐1 PRF‐s in a concentration‐dependent manner (Figure 5e).

### 2.8 Decrease of Cellular tRNAs by SLFN14 and SLFN11

**Figure 6:**
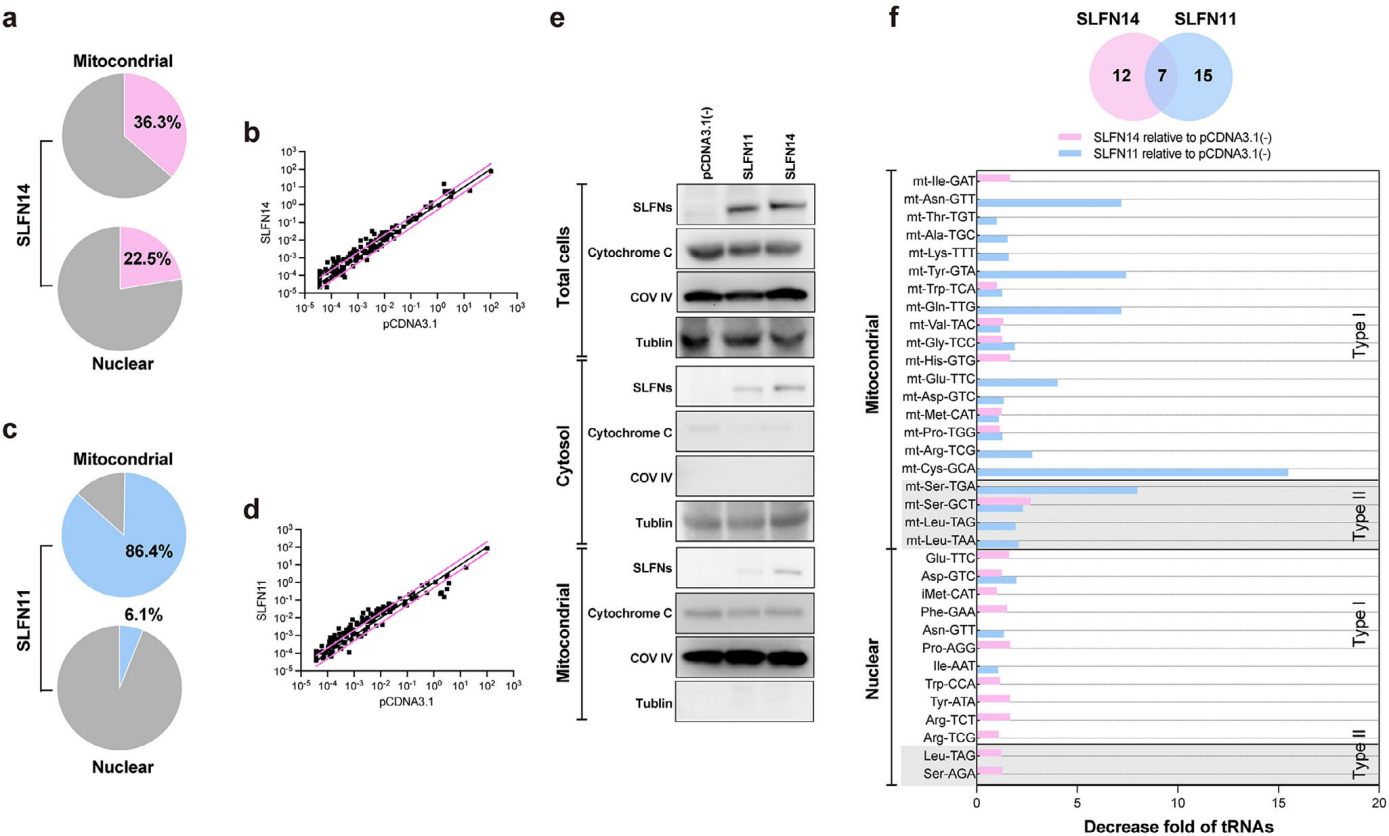
Comparison of tRNA sequencing data for SLFN14 and SLFN11 overexpressing cells. a,c) SLFN14 (pink) and SLFN11(blue) overexpression reduces tRNA abundance in mitochondria and nuclear. Panel a shows the percentage of tRNA exhibiting down‐fold changes in abundance upon SLFN14 overexpression in mitochondria (top) and nucleus (below). Followed by the corresponding percentages for tRNA down‐fold changes by SLFN11 in panel c. b,d) Differential tRNA expression in response to SLFN14 and SLFN11 overexpression. Scatter plot depicting the fold change in the tRNA expression levels (2 ^ ^−ΔCt^) relative to pCDNA3.1 control. The black represents a fold change of 1 (no difference). The pink lines indicate the 2‐fold change thresholds. The plot b shows SLFN14‐overexpressing cells compared to pCDNA3.1 control, and the plot d shows SLFN11‐overexpressing cells compared pCDNA3.1 control. e) Western blot detection of SLFN11 and SLFN14. Western blot analysis was performed to assess SLFN11 and SLFN14 expression in whole cell lysates, cytoplasmic supernatants, and isolated mitochondria. COX IV served as a mitochondrial internal control, and tubulin as a cytoplasmic control. V5 antibodies were used to detect SLFN11 and SLFN14. Cytochrome C was used as a key mitochondrial protein. f) Comparison of decreased tRNA isoacceptors by SLFN14 and SLFN11. The Venn diagram shows three sections of decreased tRNA isoacceptors: unique to SLFN14 (pink), common to both (color overlay), and unique to SLFN11 (blue). The bar chart below provides a detailed representation of the Venn diagram.

To investigate the tRNA cleavage specificities of SLFN14 and SLFN11 in cells, we employed the nrStar™ human tRNA PCR array to measure the levels of all known tRNAs, including 163 nuclear‐encoded tRNAs and 22 mitochondrial tRNAs, in HEK293T cells overexpressing SLFN14 or SLFN11. Total RNAs were extracted 48 h after plasmid transformation, and the RNAs were demethylated to allow efficient reverse transcription reaction for tRNA amplification. The quantitative PCR‐based microarray profiling (**Figure** **6**a,b; Figure S9, Supporting Information) showed that SLFN14 expression decreased the nuclear‐encoded tRNAs as well as mitochondrial tRNAs. Likewise, SLFN11 expression also decreased the nuclear‐encoded and mitochondrial tRNAs (Figure 6c,d). These results suggest that SLFN14 and SLFN11 targets mitochondrial tRNAs, either directly or indirectly; however, more experimental evidence supporting this hypothesis will be needed to support this hypothesis. It worth noting that, although SLFN14 was primarily found in the cytoplasm and SLFN11 was mainly found in the nucleus (Figure 1h), overexpression of these proteins in HKE293T cells could result in the spreading of the recombinant proteins in all intracellular compartments. Indeed, both SLFN14 and SLFN11 were detectable in mitochondria (Figure 6e). Although it is unknown how SLFN14 and SLFN11 were transported into mitochondria, the presence of these enzymes could explain why mitochondrial tRNAs were cleaved (Figure 6a–d).

Using bioinformatic methods, such as TargetP v2.0, we could not detect mitochondrial targeting signals (MTS) in the amino acid sequence of SLFN14 or SLFN11. Nonetheless, the Human Protein Atlas (https://www.proteinatlas.org) data showed that among gene clusters expressed in immune cells, SLFN11 was placed in the non‐specific mitochondria cluster, implying it has functions in mitochondria. To investigate whether SLFN14 and SLFN11 can be transported into mitochondria, we isolated the mitochondria from HEK293T cells expressing V5‐tagged SLFN14 or SLFN11. The western plot experiments showed that SLFN14 and SLFN11 are present in the mitochondria (Figure 6e), which provides an explanation that both proteins could decrease mitochondrial tRNAs levels in the tRNA PCR array experiments.

Among the tRNA genes tested, SLFN14 decreased type I and II tRNAs of either nuclear‐encoded or mitochondrial (Figure 6f). By contrast, SLFN11 decreased more type II mitochondrial tRNAs, including mt‐Ser‐UGA, mt‐Ser‐GCU, mt‐Leu‐UAG, and mt‐Leu‐UAA, than the nuclear‐encoded type II tRNAs (Figure 6f). Unexpectedly, some type I tRNAs that were also decreased by SLFN11 efficiently, among which mt‐Cys‐GCA was the most affected tRNA, which contradicts with the preference of SLFN11 of cleaving type II tRNAs.

## 3 Conclusion

SLFN14 and SLFN11 are two structurally characterized full‐length SLFN members in subgroup III of the SLFN protein family that share a ring‐like dimeric structure. Each SLFN monomer harbors an N‐terminal RNase core domain, a Linker domain harboring the conserved SWAVDL/SWAGDV motif, and an SF1 helicase domain. Despite the aforementioned similarities, their differences are manifold.

First, the SLFN14 dimer is more stable than the SLFN11 dimer under physiological saline concentrations, which we attribute to highly hydrophobic dimer interfaces in SLFN14. Intriguingly, when we predicted models for SLFN14 and SLNF11 as homodimers using software AlphaFold3 (AF3), the SLFN14 AF3 model was a dimer, in agreement with the cryo‐EM structure, whereas the SLFN11 AF3 model was an opened dimer, in which the interface between the helicase domains (interface I) fell apart (Figure S11, Supporting Information).

Second, SLFN14 recognizes short RNA duplexes instead of the intact tRNA molecule recognized by SLFN11. This may explain why SLFN14 has broader substrate specificity than SLFN11. Short RNA duplexes are ubiquitous features present in numerous cellular and invading RNAs, therefore, SLFN14 could participate in a variety of processes wherever dsRNA regions are exposed. For example, SLFN14 inhibits ‐1 PRF of HIV‐1 gag‐pol probably by recognizing the stimulatory hairpin downstream the slippery sequence that also contains stem‐loop regions.

Third, the C‐terminal helicase domain of SLFN14 is deficient in ATPase/helicase activity and probably has reduced ssDNA capability. By contrast, although the SLFN11 apoenzyme has non‐detectable ATPase activity due to conformational autoinhibition, it harbors a *bone fide* Walker A motif. Once the autoinhibitory state of SLFN11 apoenzyme is alleviated under certain conditions, its ATPase activity can be activated. It remains unclear what function of the C‐terminal helicase domain serves in SLFN14, if it is devoid of ATPase/helicase activity and defective in ssDNA binding? This warrants further investigations.

Forth, SLFN14 and SLFN11 both functioned as host restriction factors against HIV‐1 pseudovirus replication by inhibiting HIV‐1 protein translation in a codon‐usage‐ dependent manner. Whereas SLFN11 specifically targets type II tRNAs with rare codons, SLFN14 targets several tRNA types. Nonetheless, we provide evidence that tRNAs of rare codon were among the most decreased tRNAs by SLFN14 (Figure S10, Supporting Information), which in part supports the codon‐usage‐dependent translational inhibition.

In summary, this study provides novel insights for the enzymatic activity of SLFN14 and the structural basis for the SLFN14‐RNA interactions. We discovered novel mechanisms underlying the anti‐HIV‐1 activity of SLFN14, which is different from that of SLFN11. Our findings deepen the knowledge of SLFN protein functions and their role in human diseases.

## 4 Experimental Section

### Plasmid Construction

The human SLFN14 (SLFN14, Gene ID: 342618) and SLFN11 (SLFN11, Gene ID: 91607) genes were synthesized by Genscript, amplified via PCR, and cloned into the pCDNA3.1 expression vector with either an N‐terminal Strep‐II‐SUMO tag, V5 tag, or enhanced green fluorescent protein (eGFP). SLFN14 was additionally cloned into pFastBac1 (Thermo Fisher) with a 6×His‐SUMO fusion at the N‐terminus. Site‐directed mutagenesis PCR (Table S7, Supporting Information) was used to generate SLFN14 mutants.

The pNL4‐3.luc.R‐E‐ plasmid, encodes an Env‐defective, luciferase reporter expressing HIV‐1 genome plasmid, was kindly provided by Dr Yuxian He. The pcDNA3.1‐gag‐WT contains the wild‐type *gag* gene sequence of HIV‐1, retaining the original viral codon usage. Gag‐opti is a construct containing the *gag* gene sequence, which was optimized for human expression with high CAI.

The dual luciferase system^[^ ^29^ ^]^ employs the pDual‐HIV (‐1) construct containing the HIV‐1 ‐1PRF sequence inserted between the coding regions of Renilla (Rluc) and Firefly (Fluc) luciferases. The control pDual‐HIV (0) vector includes an additional adenine after the mutant slippery sequence to maintain Fluc and Rluc in the same reading frame.

The *Shiftless* (SFL, Gene ID: 55337) coding region was cloned into pCDNA3.1 with an N‐terminal Myc tag, as previously described protocol.^[^ ^29^ ^]^

### Cell Culture

*Spodoptera frugiperda* Sf21 insect cells (Thermo Fisher Scientific) were cultured in suspension at 28 °C in Sf‐900™ II SFM medium (Gibco), supplemented with 10 µg mL^−1^ gentamycin. For protein expression, Sf21 cells were amplified in SIM SF expression medium (Sino Biological). Human embryonic kidney (HEK) 293F and HEK293T cells were cultured in a humidified incubator at 37 °C with 5% CO_2_. HEK293F cells were maintained in SMM 293T II medium (Sino Biological). While HEK293T cells were cultured in Dulbecco's Modified Eagle Medium (DMEM, Gibco) supplemented with 1% penicillin‐streptomycin and 10% fetal bovine serum.

### Protein Expression and Purification

The genes encoding various SLFN14 constructs were chemically synthesized by GenScript (Table S6, Supporting Information). These included a mutant designed for cryo‐EM sample preparation (SLFN14 E211A‐C365S‐C775S‐C808S, denoted SLFN14^mut^) and wild‐type SLFN14 (WT‐SLFN14) for biochemical characterization and antiviral assays.

For cryo‐EM sample preparation, SLFN14^mut^ was cloned into the pFastBac1 vector (Thermo Fisher) with a 6×His‐SUMO tag engineered at the N‐terminus. The plasmid was transfected to DH10Bac competent cells to generate recombinant bacmid. Following bacmid transfection into Sf21 cells, recombinant baculoviruses were amplified for infection of 1.6L Sf21 cells (at a density of 3.0×10^6^ cells/mL). Cells were cultured at 28°C and 120 rpm for 48 h post‐infection before harvesting. Harvested cells were collected by centrifugation, resuspended in lysis buffer (50 mm Tris‐HCl, pH 7.5, 150 mm NaCl, 25 mm imidazole, and 1 mm phenylmethylsulfonyl fluoride (PMSF)), and disrupted by ultrasonication. The cell lysate was clarified by centrifugation at 47,850 *g* for 1 h at 4°C. The supernatant was loaded onto Ni‐NTA resin (Qiagen) pre‐equilibrated with the lysis buffer. The resin was washed with increasing concentrations of NaCl in lysis buffer: 3 column volumes (CVs) of 500 mm NaCl, followed by 3 CVs of 900 mm NaCl, and 3 CVs of 1.5 m NaCl. The bound protein was eluted with lysis buffer containing 200 mm imidazole. The eluate was then loaded onto a HiTrap Heparin HP column (Cytiva) pre‐equilibrated with buffer A (20 mm HEPES pH 7.5, 2 mm MgCl_2_, and 3 mm dithiothreitol (DTT)), and eluted with a linear gradient of buffer B (20 mm HEPES pH 7.5, 1 m KCl, 2 mm MgCl_2_, and 3 mm DTT). Finally, the protein was purified by size‐exclusion chromatography on a Superdex 200 10/300 column (GE Healthcare) in buffer containing 20 mm HEPES pH 7.5, 150 mm KCl, 2 mm MgCl_2_, and 3 mm DTT. Peak fractions were used for cryo‐EM sample preparation.

To enhance the stability of the WT‐SLFN14, the gene was codon‐optimized and cloned into the pCDNA3.1 vector, incorporating an N‐terminal strep‐strep‐SUMO tag. The plasmid was transiently transfected into HEK293F cells using polyethyleneimine (PEI, Polysciences). Cells were cultured in suspension at 37°C with 5% CO_2_ and 130 rpm for 72 h. After 72 h, cells were harvested by centrifugation and resuspended in lysis buffer (100 mm Tris‐HCl, pH 7.5, 150 mm NaCl, 5 mm EDTA, and 1 mm PMSF). Cell lysis was achieved by ultrasonication, followed by clarification by centrifugation. The supernatant was then loaded onto Strep‐Tactin resin (IBA Lifesciences) pre‐equilibrated with lysis buffer. The resin was washed to remove non‐specifically bound proteins using the same protocol as described for SLFN14^mut^. The bound protein was subsequently eluted with lysis buffer containing 10 mm D‐desthiobiotin (Macklin). Eluted fractions were pooled, concentrated, and further purified by size‐exclusion chromatography on a Superdex 200 10/300 column (GE Healthcare) pre‐equilibrated in buffer containing 10 mm Tris‐HCl, pH 7.5, 150 mm KCl, and 3 mm DTT. Purified WT‐SLFN14 and its mutants were analyzed by SDS‐PAGE, concentrated, and flash‐frozen in liquid nitrogen for subsequent biochemical assays.

SLFN14 mutants were expressed and purified using the same protocol as WT‐SLFN14. WT‐SLNF11 was also expressed and purified using a similar protocol, with all buffer pH values adjusted to 8.5.

### Analytical Ultracentrifugation

Sedimentation velocity experiments were performed in a Proteome Lab Optima analytical ultracentrifuge (Beckman Coulter, Brea, CA), equipped with AN‐60Ti rotor (4‐holes) and conventional double‐sector aluminum centerpieces of 12 mm optical path length, loaded with 380 µL of samples and 400 µL of buffer (20 mm HEPES pH 7.5, 2 mm MgCl_2_, 3 mm DTT and 40 mm NaCl or 150 mm NaCl). Before the run, the rotor was equilibrated for ≈8 h at 4°C in the centrifuge. And then experiments were carried out at 4 °C and 50,000 rpm, using continuous scan mode and radial spacing of 0.003 cm. Scans were collected in 30 S intervals at 280 nm. The fitting of absorbance versus cell radius data was performed using *SEDFIT* software (https://sedfitsedphat.nibib.nih.gov/software/default.aspx) and continuous sedimentation coefficient distribution c (s) model, covering range of 0–14 S. Biophysical properties of the buffer at low salt concentrations are characterized by a density (ρ) of 1.00129 g/cm^3^ and a viscosity (η) of 0.017864 P. Under physiological salt concentrations, the buffer's density increases to 1.00532 g/cm^3^ while maintaining the same viscosity (0.017864 P). Additionally, the partial specific volumes (V̅) for SLFN14 and SLFN11 proteins were 0.74610 cm^3^/g and 0.73680 cm^3^/g, respectively.

### Preparation of the Nucleic Acid Substrates

All nucleic acid substrates used in this study were chemically synthesized (GenScript) and their sequences were provided in Table S8 (Supporting Information). The nucleic acids were dissolved in RNase‐free water and annealed using PCR thermal cyclers. Annealing was performed by heating the solution to 95 °C for 1 min, followed by 91 cycles of decreasing the temperature by 1 °C per cycle. For double‐stranded nucleic acid substrates, a 1:2 molar ratio of fluorescently labeled nucleic acid to unlabeled nucleic acid was used. The mixture was then annealed using the protocol described above.

### RNase Assay

RNase activity was assessed using a previously described protocol.^[^ ^6^ ^]^ Briefly, 0.5 µm of the enzyme was incubated with 0.25 µm of the nucleic acid substrate in 1x reaction buffer (40 mm Tris‐HCl, pH 8.0, 20 mm KCl, 4 mM MgCl_2_, and 2 mm DTT) at 37°C for 30 min. Reactions were terminated by the addition of 2.5 µL of 5x loading buffer (100 mm Tris‐HCl, pH 7.5, 50% Glycerol, 1% SDS, and 5 mm EDTA). Cleavage products were then analyzed by 15% denaturing 8 m urea‐polyacrylamide gel electrophoresis (Urea–PAGE) at room temperature, run at 150 V for 40 min in 1x TBE running buffer. The gel was visualized using a ChemiDoc MP Imaging System (Bio‐Rad) and analyzed with Image Lab software (Bio‐Rad). The sequences of all nucleic acid substrates employed in this study are summarized in Table S8 (Supporting Information).

### Electrophoretic Mobility Shift Assay (EMSA)

Binding of SLFN11 to 50 nt single stranded DNA (ssDNA) substrate was monitored by electrophoretic mobility shift assay (EMSA). 37.5–300 nm SLFN14 or SLFN11 was incubated with 50 nm 5’Cy3 labelled ssDNA at 4 °C for 30 min in EMSA buffer (25 mm HEPES pH 7.5, 50 mm NaCl, 8% glycerol, 2 mm MgCl2, 1 mm DTT). Samples were applied to a Native PAGE 8% gel. The electrophoresis was performed in 0.5× TBE buffer at 100 V for 60 min at 4 °C. The gels were imaged using a ChemiDoc MP Imaging System (Bio‐Rad) and analyzed with Image Lab software (Bio‐Rad). The ssDNA sequence is AATTGGTCGTAGCAAGCTCTAGCACCGCTTAAACGCACGTACGCGCTGTC.

### Biolayer Interferometry

The experiment was conducted at 25°C in the buffer containing PBS plus 0.02% surfactant P20 and 0.1% BSA by the Octet RED96e instrument (Fortebio). The volumes for the solutions were 200 µL, and the assay was performed in black, solid, flat bottom 96‐well plates with a shaking speed of 1000 rpm. The streptavidin (SA) biosensors were activated in PBS for at least 10 min. A time of 120 s was performed for sensor check before loading the biotin labeled 50 nt ssDNA (100 nM), which took 50 s. A time of 100 s was taken for protein association in the following step. The dissociation was the last step and was performed for 300 s. The reference baseline was recorded for a sensor loaded with ligand but no analyte, which used to be subtracted to correct any systematic baseline drift. To compare the ssDNA affinity of SLFN11 and SLFN14, 1000, 500, 250, 125, 62.5 and 0 nm SLFN11 or SLFN14 were used to associate with 100 nm biotinylated ssDNA coated SA biosensors, respectively. All data were processed by Data Analysis 11. The ssDNA sequence is AATTGGTCGTAGCAAGCTCTAGCACCGCTTAAACGCACGTACGCGCTGTC.

### Fluorescence Microscopy

HEK293T cells were seeded in a 35 mm^2^ glass‐bottomed dish (NEST) and transfected with either the pCDNA3.1‐eGFP‐SLFN11 or pCDNA3.1‐eGFP‐SLFN14 plasmid using Lipofectamine 2000 (Thermo Fisher Scientific). After 24 h of transfection, cells were incubated at 37°C for 15 min in PBS containing 1 µm of Mito Tracker Deep Red FM (Yeasen Biotech) to label mitochondria. Following incubation, cells were washed with PBS and fixed with 4% paraformaldehyde for 15 min at room temperature. Nuclear staining was performed using 1 µg ml^−1^ DAPI (Thermo Fisher Scientific) for 5 min at room temperature. After staining, cells were washed three times with PBS and immediately subjected to fluorescence imaging. Imaging was acquired using an Axio Observer ApoTome 2 laser scanning confocal microscope (Carl Zeiss) equipped with a 63x oil immersion objective. Image analysis was performed using Fiji (ImageJ) software. Pearson correlation coefficients were calculated using Fiji, and plot profiles were analyzed using Zeiss Zen software.

### Sample Preparation and Cryo‐EM Data Acquisition

The short hairpin RNA (hRNA)with sequence5’‐GCAUCAGCUACCUAAAGUUCAGGUAGCUGAUG‐3’ was dissolved in a buffer containing 40 mm Tris‐HCl (pH 8.0), 20 mm KCl, 4 mm MgCl_2_, and 2 mm DTT. To assemble the SLFN14‐hRNA complex, a 1:1.2 molar ratio of protein to RNA was used, and the mixture was incubated overnight at 4°C. The following day, the complex was centrifuged to remove any insoluble. Subsequently, 3 µL of the supernatant was applied to glow‐discharged Quantifoil R1.2/1.3 Cu 300‐mesh holey carbon grids.

The grids were blotted for 5 s at a blotting force of 1 with 100% humidity at 4 °C. All grids were plunge‐frozen into liquid ethane using a Vitrobot Mark IV instrument (Thermo Fisher Scientific). Cryo‐EM images were collected at liquid nitrogen temperature under a Titan Krios microscopy operating at 300 kV, Gatan K3 Summit detector, and Prior to detection, inelastically scattered electrons were filtered out with a Bio Quantum K3 imaging filter (Gatan) using a slit width of 20 eV. Movie stacks were automatically acquired in super‐resolution mode with a physical pixel size of 0.5044 Å per pixel, with defocus values ranging from −0.8 to −2.0 um. Each micrograph stack containing 32 frames was exposed to a total electron exposure of 60 e /A^2^. EPU3.0 (Thermo Fisher Scientific) was used for automated data collection.

In total, 11 100, and 27 052 movie stacks were collected for the apo SLFN14, and SLFN14‐hairpin RNA complex datasets, respectively.

### Cryo‐EM Data Collection Processing

All micrographs were motion‐corrected using Relion's implementation of motion correction.^[^ ^30^ ^]^ Contrast Transfer Function (CTF) parameters were estimated using CTFFIND‐4.1.^[^ ^31^ ^]^ Both datasets were initially processed using Relion4.0.^[^ ^30^ ^]^

For the apo‐SLFN14 dimer, a total of 5,491,194 particles were automatically picked from 8399 micrographs and subjected to several rounds of 2D classification (Figure S3, Supporting Information). The best 2 528 635 particles were then re‐extracted and imported into cryoSPARC‐3.3.2 for further processing.^[^ ^32^ ^]^ Two rounds of 2D classification were performed, selecting 1 948 620 particles for ab‐initio reconstruction to generate four initial models.

One of the models with a final class of 873 318 particles was subjected to a non‐uniform refinement with C1 symmetry, resulting in a 2.8 Å resolution map. Using the refined map from cryoSPARC as a reference.^[^ ^32^ ^]^ Two rounds of 3D classifications were performed in Relion4.0 to screen the best particles for the final 3D refinement and reconstruction (Figures S3a and S4, Supporting Information). In the end, a reconstruction of apo‐SLFN14 at 2.84 Å resolution was obtained (with C1 symmetry applied during the refinement, Figure 2b) from a final dataset composed of 960,006 particles (Table S1, Supporting Information). The 3D auto‐refined map was sharpened using Relion's post‐processing procedure for model building and refinement. The refined map was also post‐processed by DeepEMhancer (Sanchez‐Garcia et al., 2021), this map was used for the preparation of the figures.

The SLFN14‐hRNA complex dataset was processed similarly. Briefly, 18 825 micrographs were selected from 27 052 micrographs, and 3 914 350 particles were initially picked using the apo structure of SLFN14 as a template. This was followed by several rounds of 2D classification in Relion4.0 to curate particle sets. The best particles were re‐extracted for further 3D classification, and refinement, resulting in a reconstruction of the SLFN14‐hRNA complex at 2.7 Å resolution (with C1 symmetry applied during the refinement,) from a final dataset composed of ≈2,397,691 particles (Table S1, Supporting Information), and the reconstructed map was sharpened using Relion's post‐processing procedure for model building and refinement. The refined map was also post‐processed by DeepEMhancer (Sanchez‐Garcia et al., 2021) for the preparation of the figures.

From the refined map of the SLFN14‐hRNA complex, a soft mask including the ds‐RNA region was generated (Figures S3b and S5, Supporting Information). One round of masked classification without alignments was performed to classify the particle sets into 4 classes. A class with 338 615 particles was used for the final reconstruction, resulting in a 2.88 Å resolution map. The local resolution was estimated by Relion4.0.^[^ ^30^ ^]^

### Model Building and Refinement

An initial model of SLFN14 was generated using AlphaFold2.^[^ ^33^ ^]^ This model was then rigidly fitted into the density map of the apo‐SLFN14 using UCSF Chimera,^[^ ^34^ ^]^ followed by manual adjustments in Coot.^[^ ^35^ ^]^ Real‐space refinement was subsequently performed within PHENIX.^[^ ^36^ ^]^ The overall model validation was carried out using the comprehensive validation tool (cryo‐EM) within PHENIX.^[^ ^36^ ^]^ Post‐processing and sharpening of the cryo‐EM maps were conducted in CryoSPARC, employing homogeneous refinement, non‐uniform refinement, local refinement, and DeepEMhancer (Sanchez‐Garcia et al., 2021). A summary of the cryo‐EM data collection, 3D reconstruction, and model refinement statistics is presented in Table S1 (Supporting Information). Structural figures were generated using PyMOL (Molecular Graphics System, LLC) or Chimera X.44.^[^ ^37^ ^]^

### ATPase Assay

To assess ATPase activity, 2 µm of the enzyme was incubated in the presence or absence of 1 µM of each potential stimulator (ssDNA, hRNA, or tRNA). These reactions were performed under the same conditions as the RNase assay. ATPase activity was measured at 1‐min intervals over a 6‐min reaction using the QuantiChrom ATPase/GTPase assay kit (BioAssay Systems). At each time point, the reaction was stopped by the addition of a reagent from the ATPase assay kit. The optical density (OD) of the reaction mixture was subsequently measured at 620 nm (OD620) using a SpectraMax® iD5 spectrophotometer (Molecular Devices). Data analysis was performed using GraphPad Prism 10 software.

### Helicase Assay

Helicase assay was performed using a previously established protocol.^[^ ^27^ ^]^ Briefly, 100 nm of the enzyme was incubated in a 10 µL reaction mixture containing 50 mm HEPES (pH 7.5), 5 mm MgCl_2_, 2 mm DTT, 1 mm ATP, and 50 nm of a partial duplex RNA substrate. To prevent re‐annealing of the substrate, 300 nm of unlabeled trap RNA was added to the reaction. The mixture was incubated at 37 °C for 30 min before being terminated by the addition of 2.5 µL of loading buffer. Samples were then analyzed using the same method as the RNase assay. The gel was subsequently scanned using a ChemiDoc MP Imaging System (Bio‐Rad) and analyzed with Image Lab software (Bio‐Rad).

### Transfection, Virus Production, and Luciferase Assay

HEK293T cells were co‐transfected with either pCDNA3.1‐SLFN14‐V5 (or its truncations and mutants) and the pNL4‐3. Luc. R‐.E‐ vector, or pCDNA3.1‐SLFN14‐V5 (or its truncations and mutants), and pCDNA3.1‐gag‐WT (or codon‐optimized gene) using Lipofectamine 2000 (Thermo Fisher Scientific) according to the manufacturer's protocol. 48 h post‐transfection, the cells were harvested, lysed, and used for Western blot assays.

To quantify HIV‐1 production, HEK293T cells were co‐transfected with the pNL4‐3. Luc.R‐.E‐ vector and the pCMV‐VSV‐G packaging vector, along with either empty vector (pCDNA3.1), pCDNA3.1‐SLFN14‐V5, or its truncations and mutants. 6 h post‐transfection, the supernatant was removed and replaced with fresh medium. 48 h post‐transfection, supernatants containing HIV‐1 pseudotyped virus particles were collected. These supernatants were then used to infect fresh HEK293T cells for 6 h, followed by replacement with a fresh medium. Luciferase activity was measured in the infected cells 48 h post‐infection using the Luciferase Assay Kit (Promega).

### Western Blot Analysis

Cells were harvested in ice‐cold RIPA buffer (Solarbio) and lysed for 15 min. The lysates were centrifuged at 13 000 g for 10 min to remove cellular debris. Supernatants were separated by 15% SDS‐PAGE and transferred to PVDF membranes (Millipore). Membranes were incubated with 5% non‐fat milk for 1 h at room temperature. Membranes were incubated with primary antibodies at 4 °C overnight. After washing, membranes were incubated with HRP‐conjugated secondary antibodies for 2 h at room temperature. Following further washing, membranes were developed using SuperSignal™ West Pico PLUS Chemiluminescent Substrate (Thermo Fisher Scientific) and visualized using a ChemiDoc MP Imaging System (Bio‐Rad).

### Programmed ‐1 Ribosomal Frameshifting (‐1PRF) Efficiency Assay

The ‐1PRF efficiency assay was performed as previously described.^[^ ^29^ ^]^ Briefly, HEK293T cells were co‐transfected with either empty vector (pCDNA3.1), SLF‐Myc, or pCDNA3.1‐SLFN14‐V5 expression plasmids, along with a dual‐luciferase reporter plasmid. 32 h post‐transfection, cells were lysed, and luciferase activity was measured using the Promega Dual‐Luciferase Reporter Assay System on a Promega GloMax navigator. The ‐1PRF efficiency of SLFN14 mutants was determined using the same protocol.

### tRNA PCR Array

HEK293T cells were transfected with either empty vector (pCDNA3.1) or expression plasmids encoding SLFN11‐V5 (pCDNA3.1‐SLFN11‐V5) or SLFN14‐V5 (pCDNA3.1‐SLFN14‐V5). 48 h post‐transfection, cells were lysed directly in the culture dish by adding 2 mL of TRIzol Reagent (Thermo Fisher Scientific). Total RNA was extracted from the lysates using standard procedures. RNA yield and quality assessment, followed by DNase treatment and RNA cleanup. RNA demethylation was performed to ensure accurate tRNA quantification. First‐strand cDNA synthesis was carried out using the rtStar™ tRNA‐optimized First‐Strand Synthesis Kit (Arraystar). The generated cDNA was subsequently analyzed using the nrStar™ human tRNA PCR Array (Arraystar) on the CFX96 Real‐Time PCR Detection System (Bio‐Rad). The array targets tRNA sequences, with the U6 gene serving as an internal control. Relative tRNA levels were calculated using the comparative cycle threshold (2‐^ΔΔCT^) method.

### Isolation of Mitochondria

Mitochondria were isolated from HEK293T cells using the Cell Mitochondria Isolation Kit (Beyotime Biotechnology) following the manufacturer's protocol. Briefly, cells cultured in 10 cm dishes were transfected and harvested after 48 h. Cells were washed with PBS, trypsinized with Trypsin‐EDTA Solution, and collected by centrifugation at 100g for 5 min at room temperature. The cell pellet was resuspended in 600 µL mitochondria isolation buffer supplemented with 1 mm PMSF and incubated on ice for 10 min. Cells were subjected to freeze‐thaw using liquid nitrogen, followed by centrifugation at 4 °C for 10 min. The supernatant was transferred to a new tube and centrifuged at 11 000*g* for 10 min at 4 °C to separate the cytoplasmic fraction from the mitochondrial pellet. The mitochondrial pellet was lysed in mitochondria lysis buffer containing 1 mM PMSF. Both cytoplasmic and mitochondrial fractions were subsequently analyzed by Western blotting.

### Quantification and Statistical Analysis

Statistical analysis was conducted using one‐way ANOVA to assess differences between groups. Data are expressed as means ± SD, derived from three independent experiments (*n* = 3 per group, as specified in the figure legends) unless otherwise specified. GraphPad Prism v10 was employed for all statistical calculations, including mean determination, SD computation, and P‐value generation via two‐tailed paired Student's t‐test. To ensure reproducibility, the number of experimental replicates and key analytical parameters (e.g., significance thresholds) are explicitly detailed in each figure legend.

## Conflict of Interest

The authors declare no conflict of interest.

## Author Contributions

M.L., D.S., and W.H. contributed equally to this work and are joint first authors. S.C., M.L., and W.H. designed the project. D.S., M.L., and W.H. prepared the sample for cryo‐EM studies; D.S., W.H., and Y.W. collected the EM images and determined the EM structures. S.C. W.H. and M.L. wrote and revised the paper. W.H. and M.L. established the expression and purification of the recombinant SLFN14 and SLFN11 proteins and carried out biochemical characterization of the proteins, and performed anti‐HIV assays. S.C. W.H. and M.L. analyzed the data. H.F. assisted in EM studies; Y.Z. Z.L. and B.Q. provided assistance to functional analysis and biochemical characterizations of SLFN14. All authors reviewed the results and approved the final version of the manuscript.

## Supporting information



Supporting Information

## Data Availability

The atomic models of the SLFN14 apoenzyme and the SLFN14‐hairpin RNA complex have been deposited into the Protein Data Bank (PDB) with accession codes 9JR9 and 9UIE, respectively. The corresponding 3D cryo‐EM density maps have been deposited into the Electron Microscopy Data Bank (EMDB) under the accession codes EMD‐61748 and EMD‐61749, respectively.

## References

[1] E. Mavrommatis , E. N. Fish , L. C. Platanias , J. Interferon. Cytokine Res. 2013, 33, 206. 23570387 10.1089/jir.2012.0133 PMC3624771

[2] O. Bustos , S. Naik , G. Ayers , C. Casola , M. A. Perez‐Lamigueiro , P. T. Chippindale , E. J. Pritham , E. de la Casa‐Esperon , Gene 2009, 447, 1. 19619625 10.1016/j.gene.2009.07.006 PMC9533870

[3] C. W. Garvie , X. Wu , M. Papanastasiou , S. Lee , J. Fuller , G. R. Schnitzler , S. W. Horner , A. Baker , T. Zhang , J. P. Mullahoo , L. Westlake , S. H. Hoyt , M. Toetzl , M. J. Ranaghan , L. de. Waal , J. McGaunn , B. Kaplan , F. Piccioni , X. Yang , M. Lange , A. Tersteegen , D. Raymond , T. A. Lewis , S. A. Carr , A. D. Cherniack , C. T. Lemke , M. Meyerson , H. Greulich , Nat. Commun. 2021, 12, 4375. 34272366 10.1038/s41467-021-24495-w PMC8285493

[4] F. J. Metzner , E. Huber , K. P. Hopfner , K. Lammens , Nucleic Acids Res. 2022, 50, 1147. 35037067 10.1093/nar/gkab1278 PMC8789055

[5] F. J. Metzner , S. J. Wenzl , M. Kugler , S. Krebs , K. P. Hopfner , K. Lammens , Nat. Commun. 2022, 13, 5464. 36115853 10.1038/s41467-022-33123-0 PMC9482658

[6] J. Y. Yang , X. Y. Deng , Y. S. Li , X. C. Ma , J. X. Feng , B. Yu , Y. Chen , Y. L. Luo , X. Wang , M. L. Chen , Z. X. Fang , F. X. Zheng , Y. P. Li , Q. Zhong , T. B Kang , L. B. Song , R. H. Xu , M. S. Zeng , W. Chen , H. Zhang , W. Xie , S. Gao , Nat. Commun. 2018, 9, 1165. 29563550 10.1038/s41467-018-03544-x PMC5862951

[7] S. Al‐Marsoummi , E. E. Vomhof‐DeKrey , M. D. Basson , Cells 2021, 10, 2238. 34571887 10.3390/cells10092238 PMC8465726

[8] J. Barretina , G. Caponigro , N. Stransky , K. Venkatesan , A. A. Margolin , S. Kim , C. J. Wilson , J. Lehar , G. V. Kryukov , D. Sonkin , A. Reddy , M. Liu , L. Murray , M. F. Berger , J. E. Monahan , P. Morais , J. Meltzer , A. Korejwa , J. Jané‐Valbuena , F. A. Mapa , J. Thibault , E. Bric‐Furlong , P. Raman , A. Shipway , I. H. Engels , J. Cheng , G. K. Yu , J. Yu , P. A. Jr , M. de Silva , et al., Nature 2012, 483, 603. 22460905 10.1038/nature11003 PMC3320027

[9] E. T. Kim , J. M. Dybas , K. Kulej , E. D. Reyes , A. M. Price , L. N. Akhtar , A. Orr , B. A. Garcia , C. Boutell , M. D. Weitzman , Nat. Microbiol. 2021, 6, 234. 33432153 10.1038/s41564-020-00826-3 PMC7856100

[10] E. T. Kim , M. D. Weitzman , Viruses 2022, 14, 442.35216035

[11] P. Hou , W. Hao , B. Qin , M. Li , R. Zhao , S. Cui , Nucleic Acids Res. 2023, 51, 7053. 37293979 10.1093/nar/gkad509 PMC10359600

[12] M. Li , E. Kao , X. Gao , H. Sandig , K. Limmer , M. Pavon‐Eternod , T. E. Jones , S. Landry , T. Pan , M. D. Weitzman , M. David , Nature 2012, 491, 125. 23000900 10.1038/nature11433 PMC3705913

[13] N. J. Boon , R. A. Oliveira , P. R. Körner , A. Kochavi , S. Mertens , Y. Malka , R. Voogd , S. E. M. van der Horst , M. A. Huismans , L. P. Smabers , J. M Draper , L. F. A. Wessels , P. Haahr , J. M. L. Roodhart , T. N. M. Schumacher , H. J. Snippert , R. Agami , T. R. Brummelkamp , Science 2024, 384, 785. 38753784 10.1126/science.adh7950

[14] S. A. Ragland , J. C. Kagan , Sci. Immunol. 2024, 9, adp4474. 10.1126/sciimmunol.adp4474 PMC12337006 38875318

[15] P. Zhang , X. Hu , Z. Li , Q. Liu , L. Liu , Y. Jin , S. Liu , X. Zhao , J. Wang , D. Hao , H. Z. Chen , D. P. Liu , Science immunology 2024, 9, adj5465. 10.1126/sciimmunol.adj5465 38875319

[16] S. J. Fletcher , V. P. Pisareva , A. O. Khan , A. Tcherepanov , N. V. Morgan , A. V. Pisarev , RNA 2018, 24, 939. 29678925 10.1261/rna.066415.118 PMC6004054

[17] V. P. Pisareva , I. A. Muslimov , A. Tcherepanov , A. V. Pisarev , Biochemistry 2015, 54, 3286. 25996083 10.1021/acs.biochem.5b00302 PMC4461289

[18] R. K. Seong , S. W. Seo , J. A. Kim , S. J. Fletcher , N. V. Morgan , M. Kumar , Y. K. Choi , O. S. Shin , Immunobiology 2017, 222, 979. 28734654 10.1016/j.imbio.2017.07.002 PMC5990420

[19] C. Valenzuela , S. Saucedo , M. Llano , Viruses 2024, 16, 502. 38675845 10.3390/v16040502 PMC11054720

[20] C. Marconi , C. A. Di Buduo , S. Barozzi , F. Palombo , S. Pardini , C. Zaninetti , T. Pippucci , P. Noris , A. Balduini , M. Seri , A. Pecci , Thromb. Haemost. 2016, 115, 1076. 26769223 10.1160/TH15-11-0884

[21] S. J. Fletcher , B. Johnson , G. C. Lowe , D. Bem , S. Drake , M. Lordkipanidzé , I. S. Guiú , B. Dawood , J. Rivera , M. A. Simpson , M. E. Daly , J. Motwani , P. W. Collins , S. P. Watson , N. V. Morgan , J. Clin. Invest. 2015, 125, 3600. 26280575 10.1172/JCI80347 PMC4588283

[22] D. Polokhov , D. Fedorova , A. Ignatova , E. Ponomarenko , E. Rashevskaya , A. Martyanov , N. Podoplelova , M. Aleksenko , I. Mersiyanova , E. Seregina , A. Poletaev , E. Truchina , E. Raykina , S. Plyasunova , G. Novichkova , P. Zharkov , M. Panteleev , Orphanet J. Rare Dis. 2023, 18, 74. 37041648 10.1186/s13023-023-02675-9 PMC10091655

[23] R. J. Stapley , V. P. Pisareva , A. V. Pisarev , N. V. Morgan , Platelets 2020, 31, 407. 31378119 10.1080/09537104.2019.1648781 PMC7055508

[24] F. Ver Donck , K. Ramaekers , C. Thys , C. Van Laer , K. Peerlinck , C. Van Geet , K. Eto , V. Labarque , K. Freson , Blood 2023, 141, 2261. 36790527 10.1182/blood.2022017712 PMC10646786

[25] L. Holm , Nucleic Acids Res. 2022, 50, W210. 35610055 10.1093/nar/gkac387 PMC9252788

[26] M. Kugler , F. J. Metzner , G. Witte , K. P. Hopfner , K. Lammens , Nat Commun. 2024, 15, 10500. 39627193 10.1038/s41467-024-54833-7 PMC11615386

[27] W. Hao , J. A. Wojdyla , R. Zhao , R. Han , R. Das , I. Zlatev , M. Manoharan , M. Wang , S. Cui , PLoS Pathog. 2017, 13, 1006474. 10.1371/journal.ppat.1006474 PMC5501694 28651017

[28] M. Kobayashi‐Ishihara , K. Frazão Smutná , F. E. Alonso , J. Argilaguet , A. Esteve‐Codina , K. Geiger , M. Genescà , J. Grau‐Expósito , C. Duran‐Castells , S. Rogenmoser , R. Böttcher , J. Jungfleisch , B. Oliva , J. P. Martinez , M. Li , M. David , M. Yamagishi , M. Ruiz‐Riol , C. Brander , Y. Tsunetsugu‐Yokota , M. J. Buzon , J. Díez , A. Meyerhans , Commun. Biol. 2023, 6, 487. 37165099 10.1038/s42003-023-04841-y PMC10172343

[29] X. Wang , Y. Xuan , Y. Han , X. Ding , K. Ye , F. Yang , P. Gao , S. P. Goff , G. Gao , Cell 2019, 176, 625. 30682371 10.1016/j.cell.2018.12.030 PMC8486322

[30] D. Kimanius , L. Dong , G. Sharov , T. Nakane , S. H. W. Scheres , Biochem. J. 2021, 478, 4169. 34783343 10.1042/BCJ20210708 PMC8786306

[31] A. Rohou , N. Grigorieff , J. Struct. Biol. 2015, 192, 216. 26278980 10.1016/j.jsb.2015.08.008 PMC6760662

[32] A. Punjani , J. L. Rubinstein , D. J. Fleet , M. A. Brubaker , Nat. Methods 2017, 14, 290. 28165473 10.1038/nmeth.4169

[33] J. Jumper , R. Evans , A. Pritzel , T. Green , M. Figurnov , O. Ronneberger , K. Tunyasuvunakool , R. Bates , A. Žídek , A. Potapenko , A. Bridgland , C. Meyer , S. A. A. Kohl , A. J. Ballard , A. Cowie , B. Romera‐Paredes , S. Nikolov , R. Jain , J. Adler , T. Back , S. Petersen , D. Reiman , E. Clancy , M. Zielinsky , M. Steinegger , M. Pacholska , T. Berghammer , S. Bodenstein , D. Silver , O. Vinyals , A. W. Senior , K. Kavukcuoglu , P. Kohli , D. Hassabis , Nature 2021, 596, 583. 34265844 10.1038/s41586-021-03819-2 PMC8371605

[34] E. F. Pettersen , T. D. Goddard , C. C. Huang , G. S. Couch , D. M. Greenblatt , E. C. Meng , T. E. Ferrin , J. Comput. Chem. 2004, 25, 1605. 15264254 10.1002/jcc.20084

[35] P. Emsley , B. Lohkamp , W. G. Scott , K. Cowtan , Acta Crystallogr. D Biol. Crystallogr. 2010, 66, 486. 20383002 10.1107/S0907444910007493 PMC2852313

[36] P. D. Adams , P. V. Afonine , G. Bunkóczi , V. B. Chen , I. W. Davis , N. Echols , J. J. Headd , L. W. Hung , G. J. Kapral , R. W. Grosse‐Kunstleve , A. J. McCoy , N. W. Moriarty , R. Oeffner , R. J. Read , D. C. Richardson , J. S. Richardson , T. C. Terwilliger , P. H. Zwart , Acta Crystallogr. D Biol. Crystallogr. 2010, 66, 213. 20124702 10.1107/S0907444909052925 PMC2815670

[37] E. F. Pettersen , T. D. Goddard , C. C. Huang , E. C. Meng , G. S. Couch , T. I. Croll , J. H. Morris , T. E. Ferrin , Protein Sci. 2021, 30, 70. 32881101 10.1002/pro.3943 PMC7737788

