# Supplementary material for: Human Schlafen 14 Cleavage of Short Double‐Stranded RNAs Underpins its Antiviral Activity
Source: Adv Sci (Weinh). 2025 Jul 11;12(37):e01727. doi: 10.1002/advs.202501727 (PMC12499436; doi:10.1002/advs.202501727)
Supplement: Supplementary file 1 — Supporting Information [file ADVS-12-e01727-s001.docx]

**Supplementary materials for**

**Human Schlafen 14 Cleavage of Short Double-stranded RNAs underpins its Antiviral Activity**


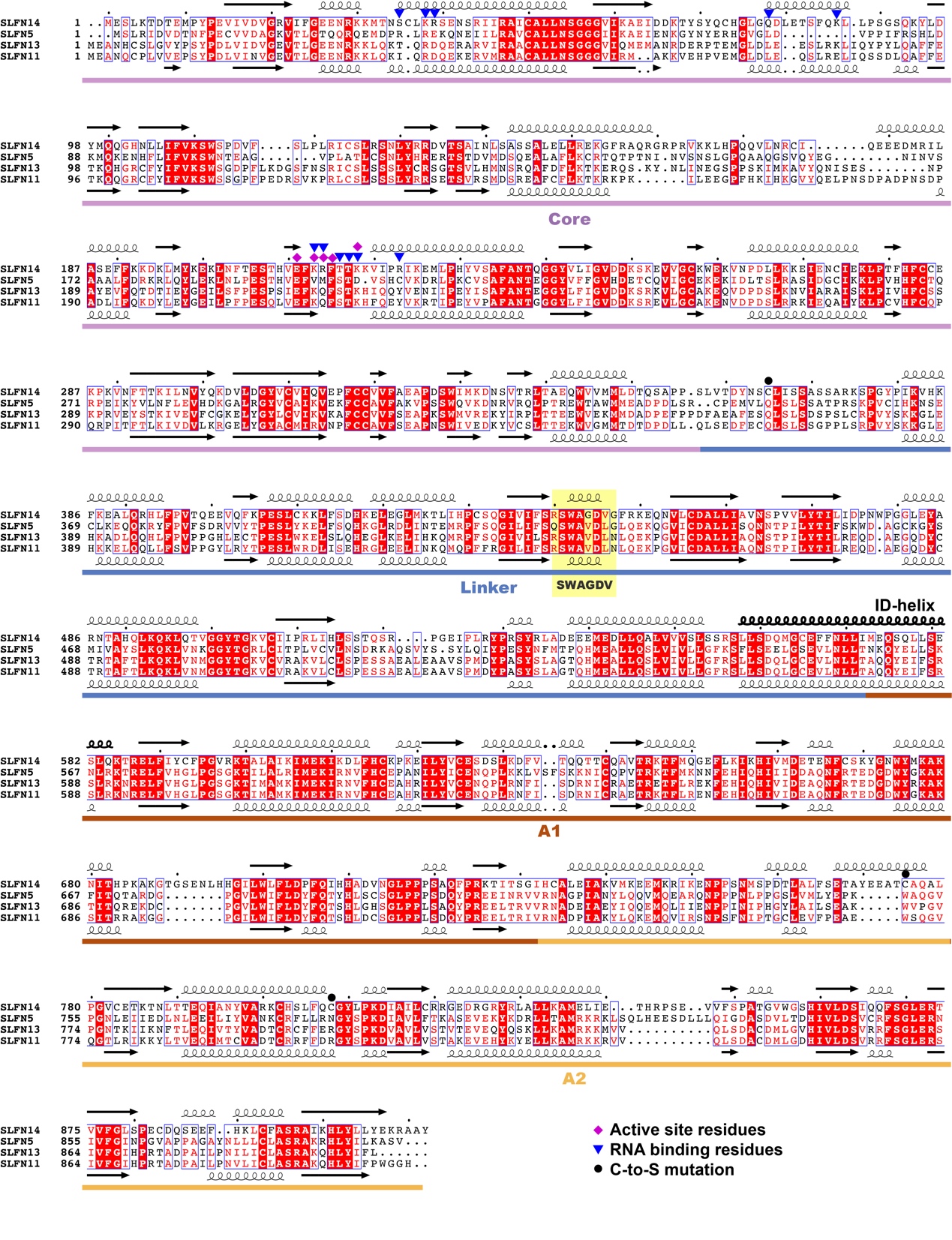


**Figure S1 Structure-based multisequence alignment of various human SLFN proteins**

Amino acid sequence of indicated human SLFN proteins including SLFN14, SLFN5, SLFN13 and SLFN11 were aligned using software Clustal Omega; invariant residues are highlighted with red background, conserved residues are colored red, non-concerted residues are colored black; secondary structural elements of SLFN14 determined from our EM structures are superimposed on top of the sequences. Key SLFN14 features: (1) domain organization is indicated by bars at the bottom of the sequences, RNase core domain is colored pink, Linker domain is colored blue, helicase domain A1 is colored brown, helicase domain A2 is colored orange. (2) RNase catalytically important residues identified in EM structures are marked by magenta diamonds. (3) RNA binding residues identified in EM structures are marked by blue triangles. (4) Engineered C-to-S mutations to improve protein stability are marked by black dots. (5) SWAGDV/SWAVDL motif is highlighted by yellow background. (6) Interdomain helix (ID-helix) connect Linker and helicase domains are highlighted thickened lines.


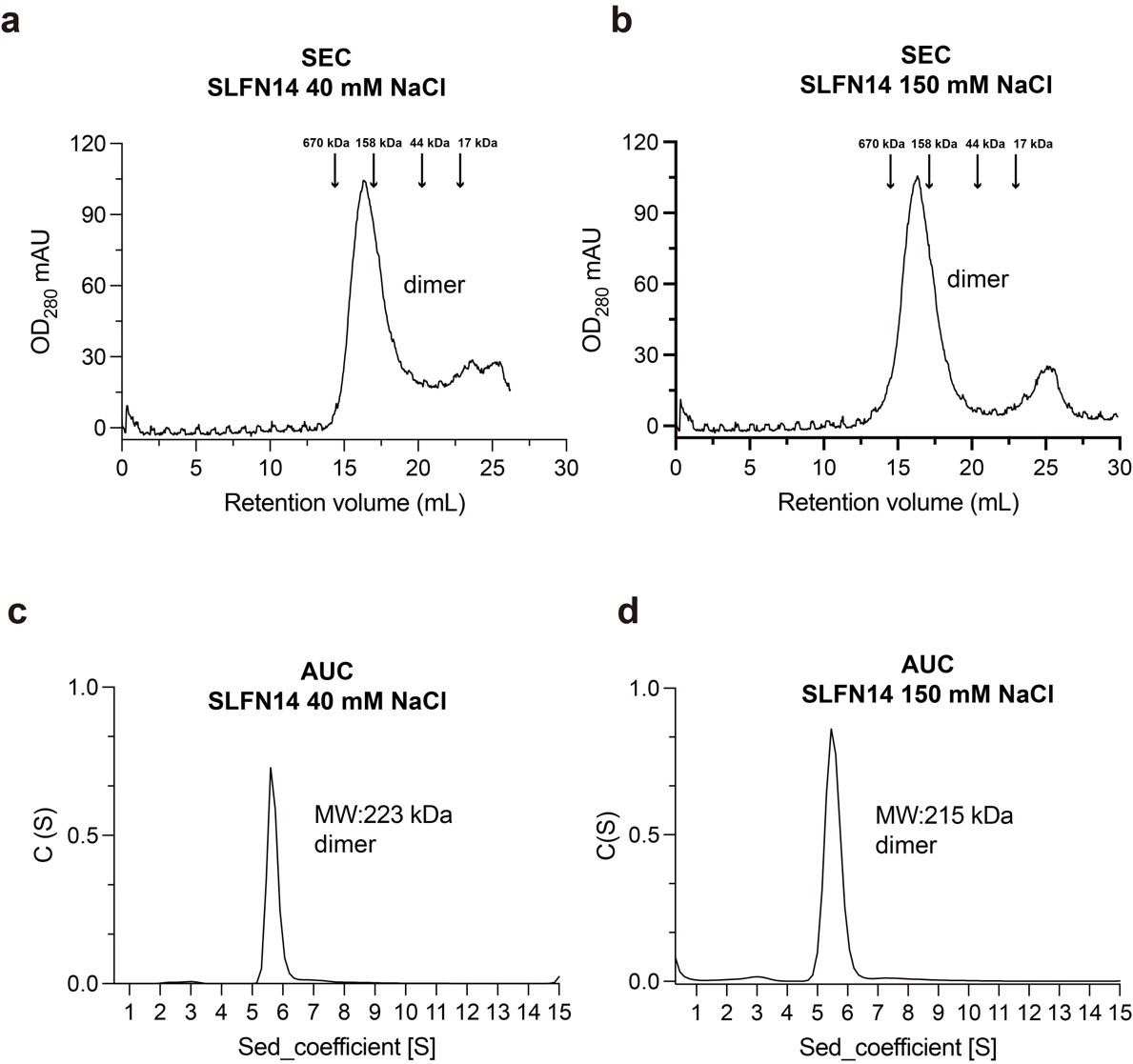


**Figure S2 Analysis of oligomerization state of human SLFN14**

1. Size-exclusion chromatograph (SEC) analysis of human SLFN14 carried out with low salt buffer (40mM NaCl); elution volume of four SEC standard proteins (thyroglobulin 670 kDa, gamma-globulin 158 kDa, ovalbumin 44 kDa and myoglobin 17 kDa) is indicated with arrows.
2. SEC analysis of human SLFN14 carried out with physiological salt buffer (150mM NaCl); elution volume of four SEC standard proteins is indicated with arrows. This is the same experiment illustrated in figure 1a
3. Analytical ultracentrifugation (AUC) analysis of human SLFN14 carried out with low salt buffer (40mM NaCl);
4. AUC analysis of human SLFN14 carried out with normal salt buffer (150mM NaCl); this is the same experiment illustrated in figure 1a.


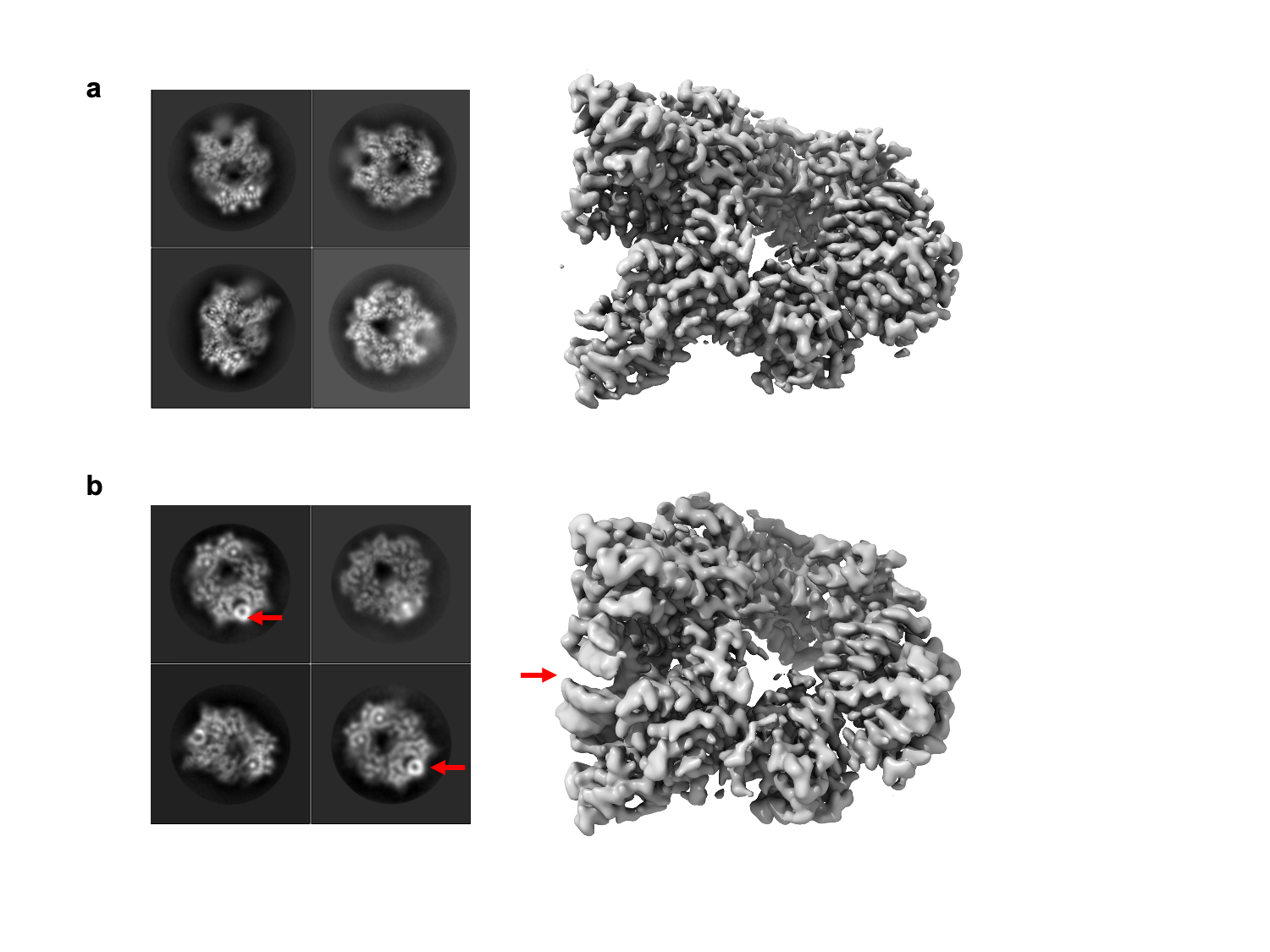


**Figure S3 2D averaged classification and 3D reconstruction of SLFN14 particles**

1. Left, four representative views of 2D-averaged SLNF14 apoenzyme particles; right, 3D reconstruction of SLNF14 apoenzyme.
2. Left, four representative views of 2D-averaged SLNF14-hairpin RNA complex particles; right, 3D reconstruction of SLNF14-hairpin RNA complex. Red arrows indicate density for hairpin RNA.


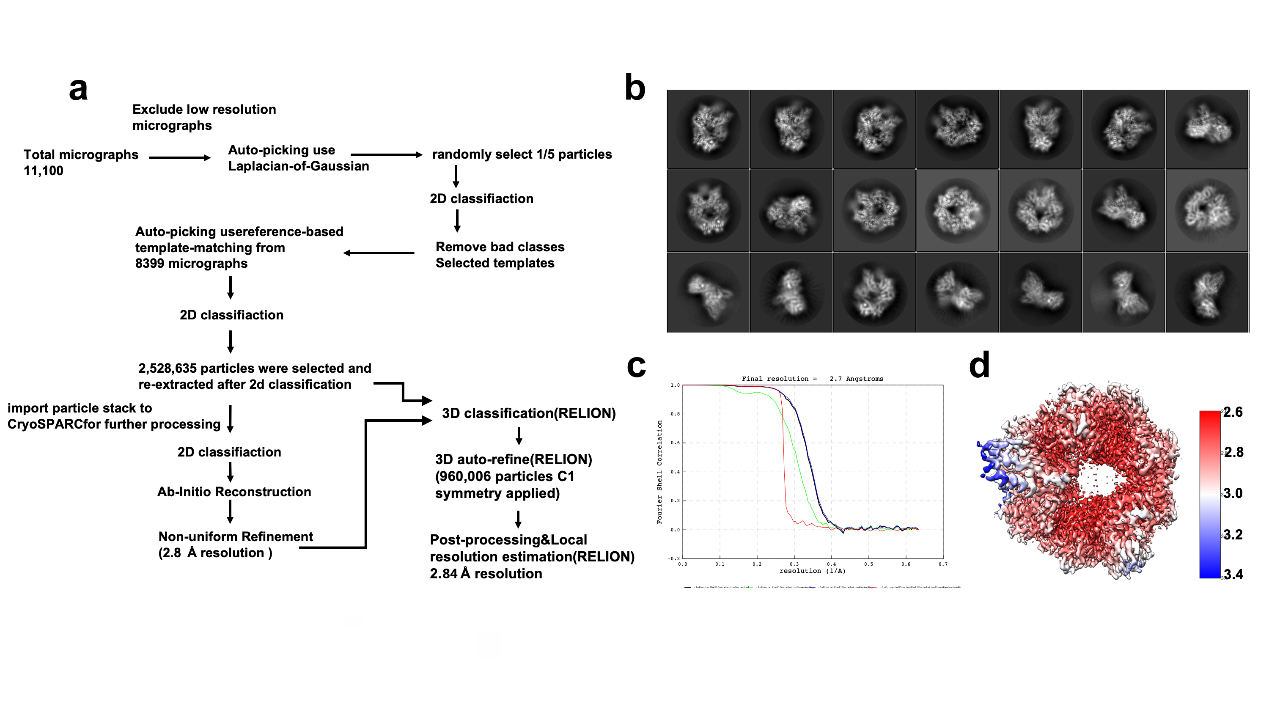


**Figure S4 Single particle cryo-EM analysis of human SLFN14 apoenzyme**

1. Workflow of cryo-EM data processing for SLFN14 apoenzyme.
2. Representative 2D averaged classification from the particles for 3D reconstruction of SLFN14 apoenzyme.
3. Gold-standard Fourier shell correlation (FSC) curves of SLFN14 apoenzyme, indicating a resolution of 2.84 Å.
4. Local resolution cryo-EM density map calculated using Relion4.0, blue-red color indicates high-low resolution.


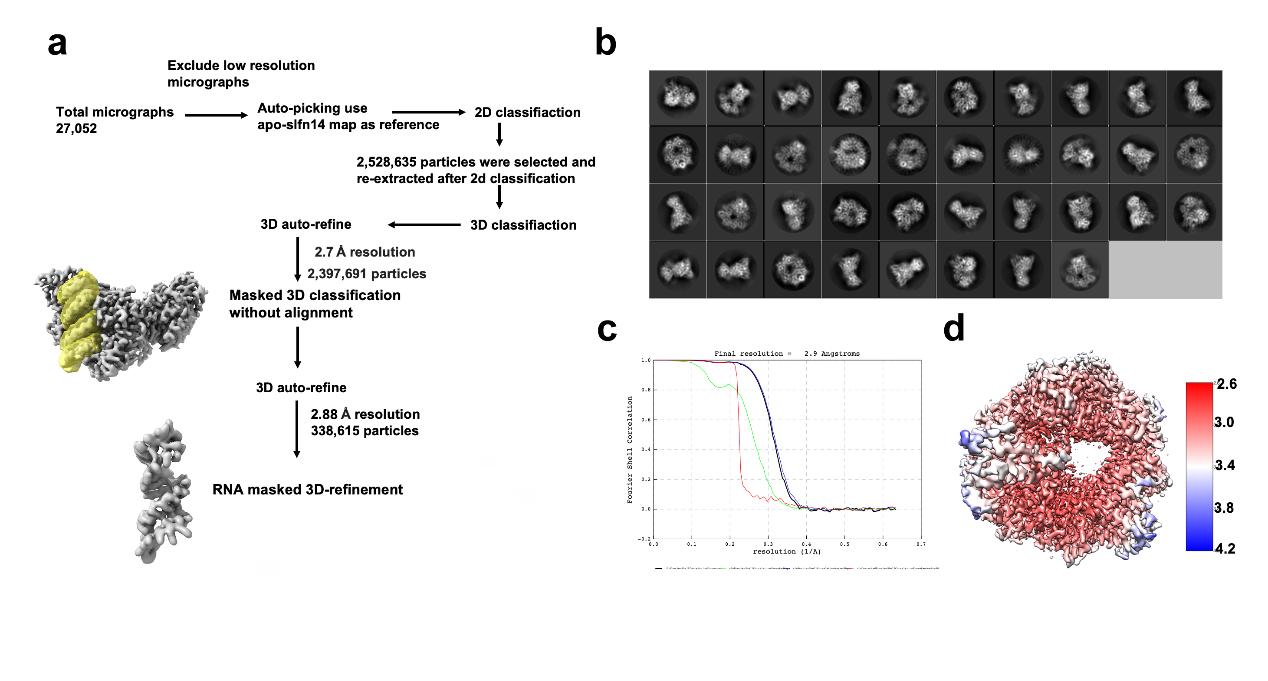


**Figure S5 Cryo-EM data analysis workflow of human SLFN14 complexed by hairpin RNA**

1. Workflow of cryo-EM data processing for SLFN14-hairpin RNA complex.
2. Representative 2D averaged classification from the particles for 3D reconstruction SLFN14- hairpin RNA complex.
3. Gold-standard Fourier shell correlation (FSC) curves of SLFN14-hairpin RNA complex, indicating a resolution of 2.88 Å.
4. Local resolution cryo-EM density map calculated using Relion4.0, blue-red color indicates high-low resolution.


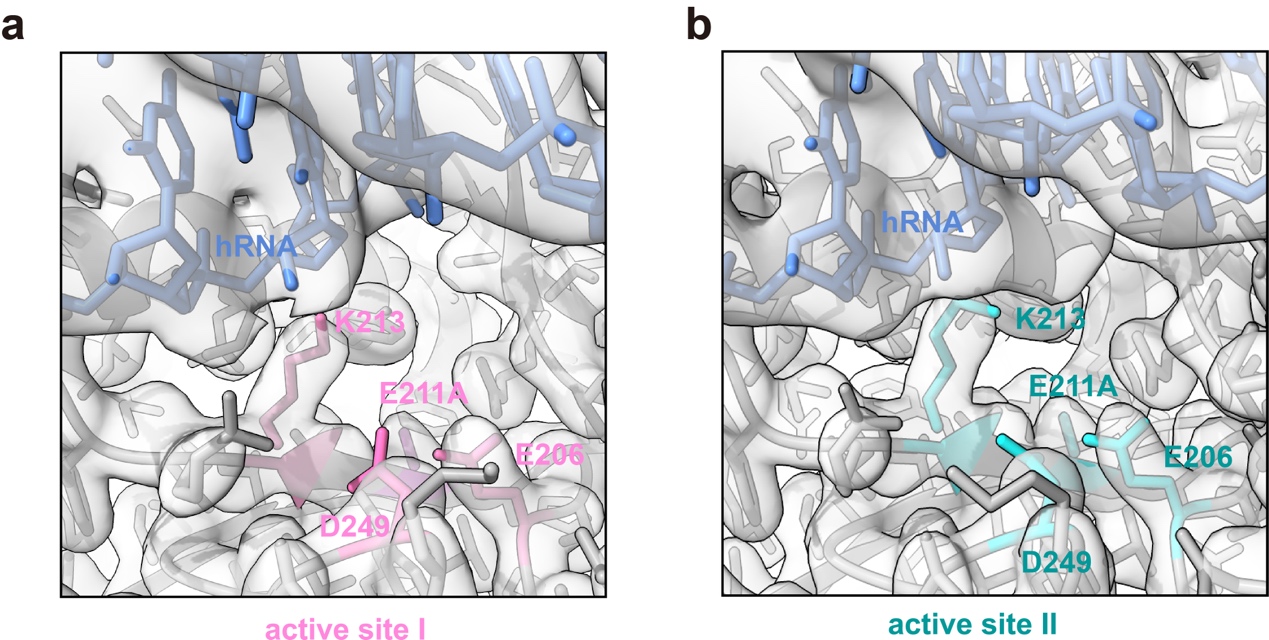


**Figure S6 Magnified view of two SLFN14 RNase active sites with superimposed final cryo-EM densities.**

Zoom-in view of SLFN14 RNase active site I (a) and II (b) shown with stick model and semitransparent final cryo-EM map. Key residues for cleavage at the active sites I and II are labeled and colored pink and cyan respectively; the bound hRNA is colored blue. No densities for ions were found in the active site.


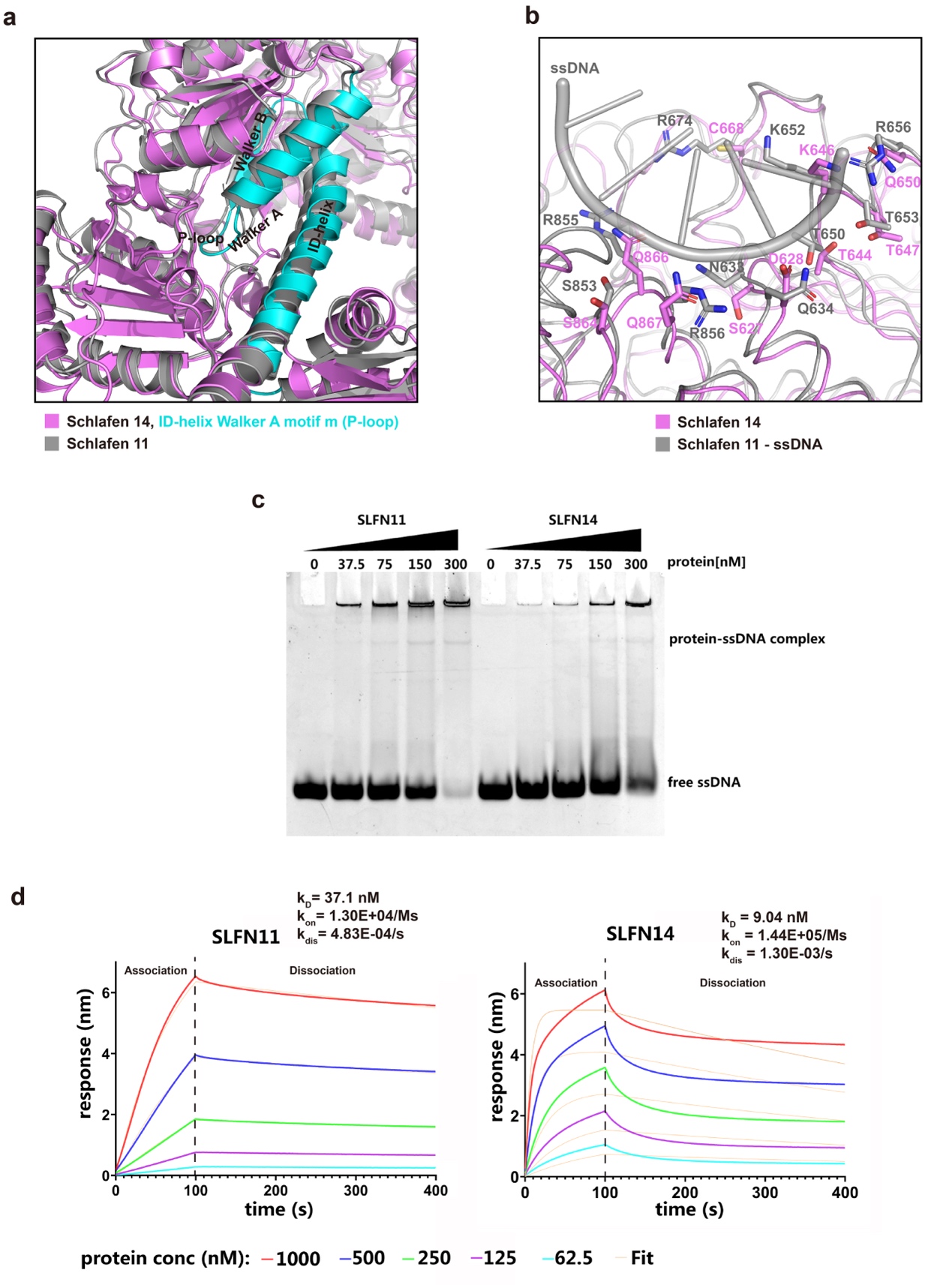


**Figure S7 Structure of SLFN14 ATPase active site and DNA binding of SLFN14**

1. SLFN14 apo structure (magenta) is superimposed with SLFN11 apo structure (gray). The ATPase active site of two proteins is magnified. Walker A & B motif, ID-helix of SLFN14 is highlighted by cyan color.
2. SLFN14 apo structure (magenta) is superimposed with SLFN11-ssDNA complex structure (PDB: 7ZES, gray). Structural counterparts of SLFN11 residues that interact with ssDNA (shown with stick model, gray) are identified in SLFN14 (shown with stick model, magenta) and labelled.
3. Electrophoretic mobility shift assay (EMSA) of human SLFN14 and SLFN11 with single-stranded DNA (ssDNA); concentration of protein used in the assays is indicated on top of the gel; SLFN-ssDNA complexes and free DNA probes are indicated.
4. Biolayer interferometry (BLI) titration of SLFN14 and SLFN11 with ssDNA. Biotinylated ssDNA (a 50-nt ssDNA used in characterizing DNA binding activity of SLFN11 ([Metzner *et al.*, 2022b](#_ENREF_23)) was immobilized on SA biosensors for binding with SLFN11 (left) and SLFN14 (right) in solution. Protein concentration in solution varied from 62.5-1000 nM during BLI titration. Binding kinetic parameters, K_D_, k_on_ and k_dis_ calculated form the BLI titrations are indicated.


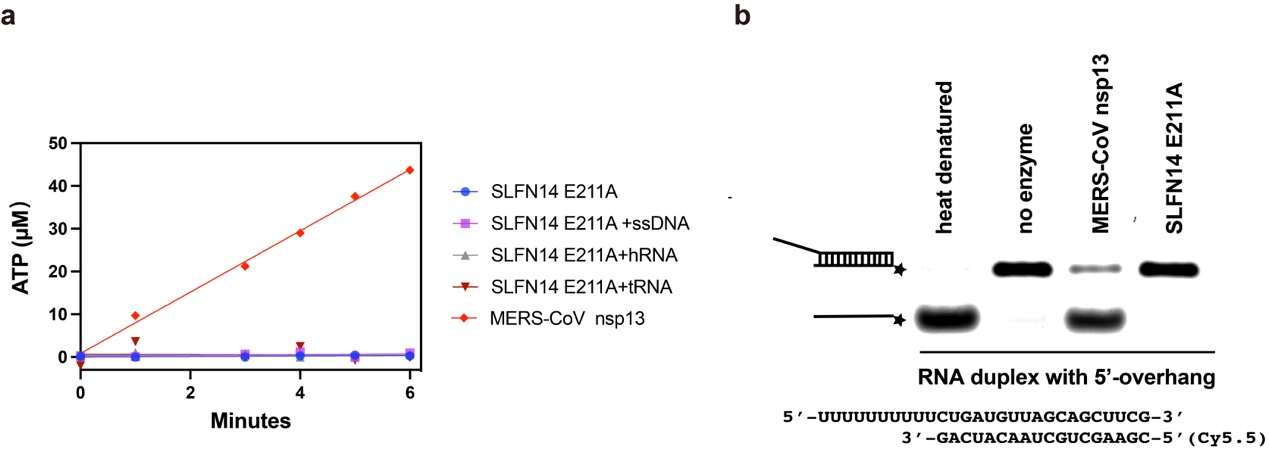


**Figure S8 SLFN14 is inactive in ATPase and helicase activity**

1. ATPase assays of SLFN14 E211A (N-terminal RNase deficient mutant) in the absence or presence of indicated nucleic acids. MERS-CoV nsp13 (characterized ATPase) served as the positive control.
2. Helicase assay of SLFN14 E211A. MERS-CoV nsp13 (characterized helicase) served as the positive control. RNA strand with cy5.5 label is marked with asterisk. The sequence of the RNA substrates with 5’ overhang is shown at the bottom.


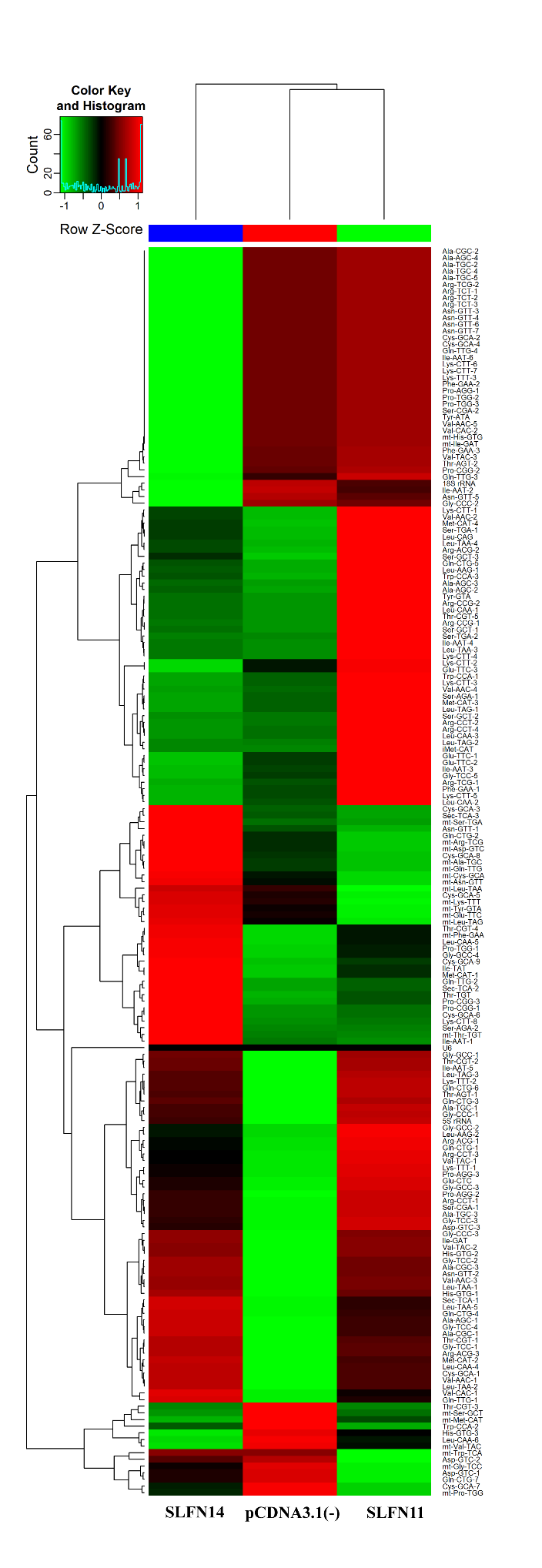


**Figure S9 human tRNA PCR microarray**

Hierarchical clustering of the all human tRNAs (163 nuclear-encoded tRNA and 22 mitochondrial tRNA species) abundance in HEK293T transfected with plasmids pCDNA3.1(-), pCDNA3.1-SLFN14 and pCDNA3.1-SLFN11. Normalized gene expression is shown by the row Z-score in the top left corner, with red indicating up-fold changes and green indicating down-fold changes.


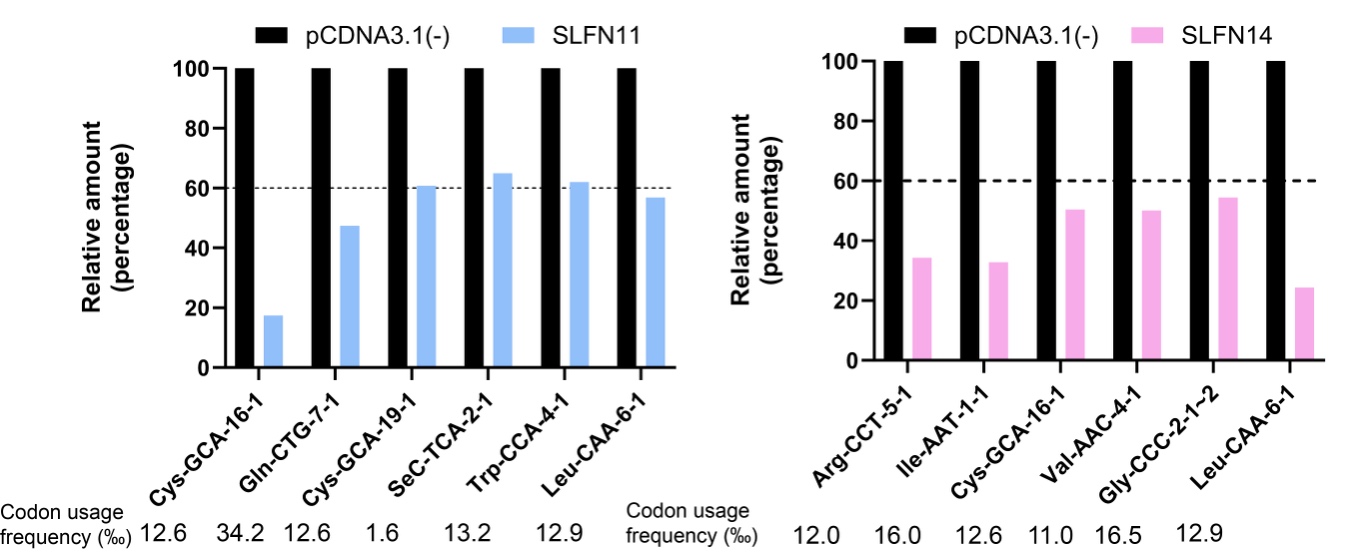


**Figure S10 Top** **decreased tRNAs by SLFN11 and SLFN14**

Bar graph illustrating top tRNA genes decreased by SLFN proteins, codon usage frequency (‰) each tRNA is indicated below

1. Top six tRNAs decreased by SLFN11 expression in HEK293T cells. Relative tRNA expression (blue) is shown as percentile comparing to empty vector pCDNA3.1 (100).
2. Top six tRNAs decreased by SLFN14 expression in HEK293T cells. Relative tRNA expression (pink) is shown as percentile comparing to empty vector pCDNA3.1 (100).


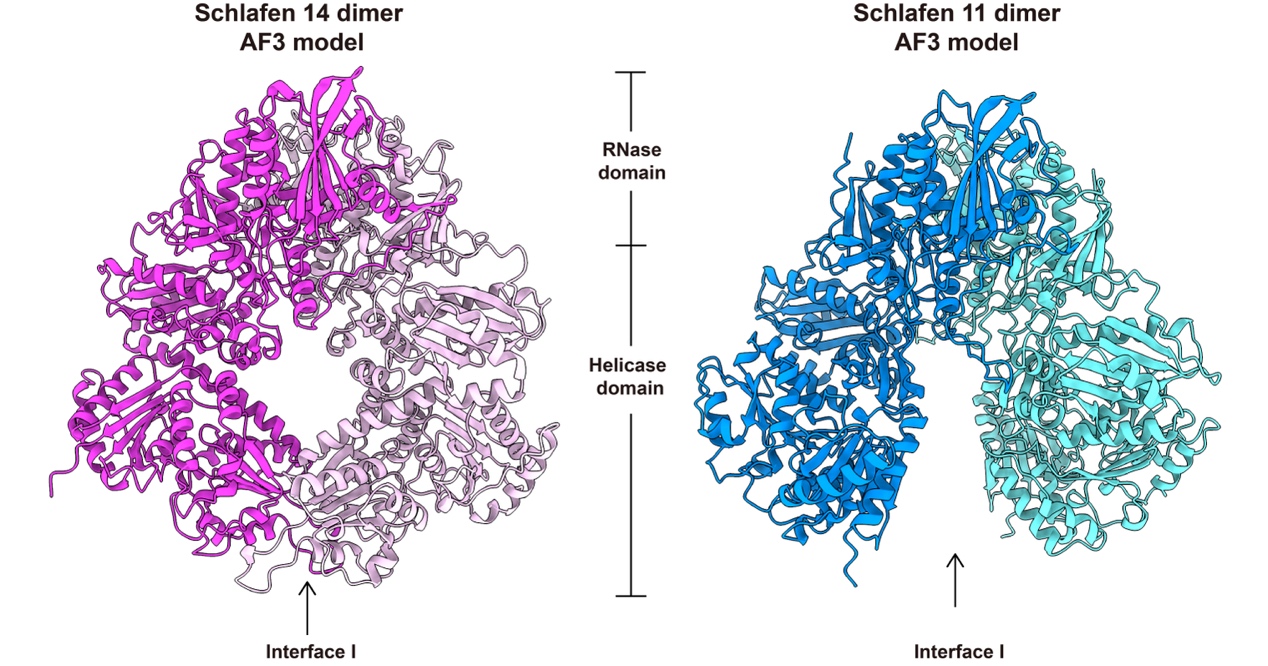


**Figure S11 AlphaFold3 models of SLFN14 and SLFN11 dimers**

Ribbon model of SLFN14 dimer (left) and SLFN11 dimer (right) predicted by software AlphaFold3. Dimer interface I (between C-terminal helicase domains of two monomers) is indicated by arrows.

# **Supplementary tables**

# **Table S1** **Cryo-EM data collection parameters, refinement and validation statistics on reconstruction**

| Name | SLFN14(E211A) | SLFN14(E211A)-hRNA complex |
| --- | --- | --- |
| **PDB ID** | 9JR9 | 9UIE |
| **EMDB ID** | EMD-61748 | EMD-61749 |
| **Data collection and processing** | | |
| Microscope | FEI Titan Krios | FEI Titan Krios |
| Voltage (keV) | 300 | 300 |
| Cumulative dose (e/Å^2) | 60 | 60 |
| Requested defocus range (um) | -0.8-2.0 | -0.8-2.0 |
| Detector | K3 | K3 |
| Pixel size (physical pixel, Å) | 0.5044 | 0.5044 |
| Dose rate (e⁻/physical pixel/sec) | 15 | 15 |
| Micrographs collected | 11,100 | 27,052 |
| **Reconstruction** |  | |
| Symmetry Imposed | C1 | C1 |
| Final particle images(no.) | 960,006 | 338,615 |
| FSC Threshold | FSC=0.143 | FSC=0.143 |
| Resolution Reported,(Å) | 2.84 | 2.88 |
| Map sharpening B factor(Å2 ) | -121.1 | -111.9 |
| **Model Refinement** |  | |
| Initial Model (PDB) | N/A | N/A |
| Protein residues (atoms) | 1731(13866) | 1803(14855) |
| Ligands (atoms) | Zn:2 | Zn:2 |
| RNA/DNA(Nucleotide) | N/A | 31 |
| **Validation** |  | |
| Ramachandran Outliers (%) | 0.00 | 0.2 |
| Clashscore (all atoms) | 7 | 6 |
| Sidechain outliers (%) | 1.5 | 3.7 |

**Table S2 Residues involved in dimerization at Interface I and II of SLFN14 and SLFN11.**

|  | **SLFN14** | **SLFN11** |
| --- | --- | --- |
| **Interface I** | R587 vs F723 | R593 vs Y722 |
|  | T586 vs P724 | N592 vs P723 |
|  | L694 vs P717 | N/A |
| **Interface II** | Y71-C73-Y98 vs V135-M183-T205 | P70-E72-T96 vs E137-P208 |
|  | Q100 vs E179 | N/A |
|  | S92 vs D134 | L90 vs S136 |

# **Table S3. Raw data of Figure 4a.**

| Exp1 | Luciferase | | | Relative pCDNA3.1(-) Luciferase activity (percentage) | | |
| --- | --- | --- | --- | --- | --- | --- |
| pCDNA3.1(-) | 4.33E+06 | 4.57E+06 | 4.77E+06 | 9.50E+01 | 1.00E+02 | 1.05E+02 |
| SLFN11 | 7.97E+04 | 1.24E+05 | 1.02E+05 | 1.75E+00 | 2.73E+00 | 2.24E+00 |
| SLFN14 | 2.10E+04 | 2.03E+04 | 3.37E+04 | 4.60E-01 | 4.47E-01 | 7.39E-01 |
| SLFN14 1-353 | 2.84E+06 | 2.23E+06 | 2.51E+06 | 6.24E+01 | 4.89E+01 | 5.52E+01 |
| SLFN14 354-end | 4.65E+06 | 5.07E+06 | 4.69E+06 | 1.02E+02 | 1.11E+02 | 1.03E+02 |
| SLFN14 E211A | 4.35E+06 | 4.42E+06 | 4.35E+06 | 9.55E+01 | 9.70E+01 | 9.55E+01 |
| SLFN14 E211A& K213A | 4.74E+06 | 4.46E+06 | 4.85E+06 | 1.04E+02 | 9.78E+01 | 1.06E+02 |

| Exp2 | Luciferase | | | Relative pCDNA3.1(-) Luciferase activity (percentage) | | |
| --- | --- | --- | --- | --- | --- | --- |
| pCDNA3.1(-) | 7.33E+05 | 6.89E+05 | 8.57E+05 | 9.65E+01 | 9.07E+01 | 1.13E+02 |
| SLFN11 | 1.07E+04 | 8.82E+03 | 8.58E+03 | 1.40E+00 | 1.16E+00 | 1.13E+00 |
| SLFN14 | 7.04E+03 | 8.98E+03 | 9.70E+03 | 9.26E-01 | 1.18E+00 | 1.28E+00 |
| SLFN14 1-353 | 4.07E+05 | 4.38E+05 | 4.69E+05 | 5.35E+01 | 5.77E+01 | 6.18E+01 |
| SLFN14 354-end | 8.59E+05 | 7.60E+05 | 6.98E+05 | 1.13E+02 | 1.00E+02 | 9.19E+01 |
| SLFN14 E211A | 8.14E+05 | 9.41E+05 | 7.38E+05 | 1.07E+02 | 1.24E+02 | 9.72E+01 |
| SLFN14 E211A& K213A | 8.32E+05 | 8.66E+05 | 7.97E+05 | 1.10E+02 | 1.14E+02 | 1.05E+02 |

| Exp3 | Luciferase | | | Relative pCDNA3.1(-) Luciferase activity (percentage) | | |
| --- | --- | --- | --- | --- | --- | --- |
| pCDNA3.1(-) | 3.64E+05 | 3.79E+05 | 3.78E+05 | 9.75E+01 | 1.01E+02 | 1.01E+02 |
| SLFN11 | 6.24E+03 | 4.26E+03 | 7.05E+03 | 1.67E+00 | 1.14E+00 | 1.88E+00 |
| SLFN14 | 4.07E+03 | 5.17E+03 | 5.71E+03 | 1.09E+00 | 1.38E+00 | 1.53E+00 |
| SLFN14 1-353 | 2.76E+05 | 2.25E+05 | 2.51E+05 | 7.39E+01 | 6.01E+01 | 6.70E+01 |
| SLFN14 354-end | 3.80E+05 | 3.60E+05 | 3.99E+05 | 1.02E+02 | 9.64E+01 | 1.07E+02 |
| SLFN14 E211A | 4.40E+05 | 4.06E+05 | 3.72E+05 | 1.18E+02 | 1.09E+02 | 9.96E+01 |
| SLFN14 E211A& K213A | 4.27E+05 | 4.15E+05 | 4.21E+05 | 1.14E+02 | 1.11E+02 | 1.13E+02 |

Luciferase activities were measured with the Luminescence Quick Read Reporter Assay System.

# **Table S4. Raw data of Figure 4e.**

| Exp1 |  | HIV-1(0) | | | HIV-1(-1) | | |
| --- | --- | --- | --- | --- | --- | --- | --- |
| pCDNA3.1(-) | Fluc | 5.28E+08 | 5.60E+08 | 5.70E+08 | 3.32E+07 | 3.60E+07 | 4.39E+07 |
|  | Rluc | 1.52E+09 | 1.57E+09 | 1.59E+09 | 5.37E+08 | 5.75E+08 | 6.66E+08 |
| Relative Fluc/Rluc*100 | | 9.60E+01 | 1.02E+02 | 1.02E+02 | 9.74E+01 | 9.88E+01 | 1.04E+02 |
| SLFN14 | Fluc | 8.05E+07 | 9.04E+07 | 7.25E+07 | 3.11E+06 | 3.33E+06 | 3.26E+06 |
|  | Rluc | 2.13E+08 | 2.37E+08 | 1.87E+08 | 9.68E+07 | 9.99E+07 | 8.76E+07 |
| Relative Fluc/Rluc*100 | | 1.08E+02 | 1.09E+02 | 1.11E+02 | 5.06E+01 | 5.26E+01 | 5.87E+01 |
| SFL | Fluc | 3.82E+08 | 3.94E+08 | 3.62E+08 | 8.47E+07 | 8.53E+07 | 8.39E+07 |
|  | Rluc | 1.07E+09 | 1.08E+09 | 9.96E+08 | 1.67E+09 | 1.69E+09 | 1.63E+09 |
| Relative Fluc/Rluc*100 | | 1.02E+02 | 1.04E+02 | 1.04E+02 | 7.99E+01 | 7.97E+01 | 8.11E+01 |

| Exp2 |  | HIV-1(0) | | | HIV-1(-1) | | |
| --- | --- | --- | --- | --- | --- | --- | --- |
| pCDNA3.1(-) | Fluc | 1.04E+09 | 1.01E+09 | 9.72E+08 | 7.60E+07 | 8.21E+07 | 8.25E+07 |
|  | Rluc | 3.25E+09 | 2.98E+09 | 2.75E+09 | 8.58E+08 | 8.75E+08 | 8.81E+08 |
| Relative Fluc/Rluc*100 | | 9.48E+01 | 1.00E+02 | 1.05E+02 | 9.63E+01 | 1.02E+02 | 1.02E+02 |
| SLFN14 | Fluc | 6.37E+08 | 6.37E+08 | 6.60E+08 | 1.16E+07 | 1.20E+07 | 1.20E+07 |
|  | Rluc | 1.79E+09 | 1.80E+09 | 1.83E+09 | 2.39E+08 | 2.32E+08 | 2.36E+08 |
| Relative Fluc/Rluc*100 | | 1.06E+02 | 1.05E+02 | 1.07E+02 | 5.06E+01 | 5.26E+01 | 5.87E+01 |
| SFL | Fluc | 8.69E+08 | 9.40E+08 | 9.53E+08 | 4.90E+07 | 4.86E+07 | 4.89E+07 |
|  | Rluc | 2.66E+09 | 2.81E+09 | 2.90E+09 | 7.10E+08 | 7.15E+08 | 7.31E+08 |
| Relative Fluc/Rluc*100 | | 9.71E+01 | 9.95E+01 | 9.77E+01 | 7.50E+01 | 7.40E+01 | 7.27E+01 |

| Exp3 |  | HIV-1(0) | | | HIV-1(-1) | | |
| --- | --- | --- | --- | --- | --- | --- | --- |
| pCDNA3.1(-) | Fluc | 1.11E+09 | 1.16E+09 | 1.14E+09 | 8.06E+07 | 8.18E+07 | 7.37E+07 |
|  | Rluc | 3.90E+09 | 3.96E+09 | 3.79E+09 | 1.23E+09 | 1.21E+09 | 1.11E+09 |
| Relative Fluc/Rluc*100 | | 9.73E+01 | 1.00E+02 | 1.03E+02 | 9.85E+01 | 1.01E+02 | 1.01E+02 |
| SLFN14 | Fluc | 5.24E+08 | 5.57E+08 | 5.35E+08 | 2.79E+06 | 2.67E+06 | 2.52E+06 |
|  | Rluc | 1.61E+09 | 1.66E+09 | 1.61E+09 | 8.35E+07 | 7.48E+07 | 7.01E+07 |
| Relative Fluc/Rluc*100 | | 1.12E+02 | 1.15E+02 | 1.14E+02 | 5.01E+01 | 5.36E+01 | 5.40E+01 |
| SFL | Fluc | 1.04E+09 | 1.12E+09 | 1.02E+09 | 2.39E+07 | 2.00E+07 | 1.85E+07 |
|  | Rluc | 4.11E+09 | 3.98E+09 | 3.67E+09 | 4.46E+08 | 3.76E+08 | 3.45E+08 |
| Relative Fluc/Rluc*100 | | 8.67E+01 | 9.59E+01 | 9.48E+01 | 8.05E+01 | 8.00E+01 | 8.06E+01 |

Luciferase activities were measured with the Dual-Luciferase Reporter Assay System.

Fluc: Firefly luciferase; Rluc: Renilla luciferase.

# **Table S5. Raw data of Figure 4f.**

| Exp1 |  | HIV-1(-1) | | |
| --- | --- | --- | --- | --- |
| pCDNA3.1(-) | Fluc | 1.35E+08 | 1.37E+08 | 1.30E+08 |
|  | Rluc | 2.34E+09 | 2.34E+09 | 2.21E+09 |
| Relative Fluc/Rluc*100 | | 9.86E+01 | 1.00E+02 | 1.01E+02 |
| SLFN14 | Fluc | 1.83E+07 | 1.86E+07 | 1.67E+07 |
|  | Rluc | 5.06E+08 | 5.08E+08 | 4.67E+08 |
| Relative Fluc/Rluc*100 | | 6.19E+01 | 6.26E+01 | 6.13E+01 |
| SLFN14 E211A | Fluc | 6.51E+07 | 7.01E+07 | 6.63E+07 |
|  | Rluc | 1.07E+09 | 1.12E+09 | 1.07E+09 |
| Relative Fluc/Rluc*100 | | 1.04E+02 | 1.07E+02 | 1.06E+02 |
| SLFN14 E211A-K213A | Fluc | 6.40E+07 | 7.33E+07 | 6.46E+07 |
|  | Rluc | 1.03E+09 | 1.17E+09 | 1.05E+09 |
| Relative Fluc/Rluc*100 | | 1.06E+02 | 1.08E+02 | 1.05E+02 |

| Exp2 |  | HIV-1(-1) | | |
| --- | --- | --- | --- | --- |
| pCDNA3.1(-) | Fluc | 1.56E+08 | 1.65E+08 | 1.42E+08 |
|  | Rluc | 2.74E+09 | 2.73E+09 | 2.38E+09 |
| Relative Fluc/Rluc*100 | | 9.64E+01 | 1.02E+02 | 1.01E+02 |
| SLFN14 | Fluc | 2.20E+07 | 2.16E+07 | 1.90E+07 |
|  | Rluc | 5.89E+08 | 5.72E+08 | 5.35E+08 |
| Relative Fluc/Rluc*100 | | 6.35E+01 | 6.41E+01 | 6.03E+01 |
| SLFN14 E211A | Fluc | 7.09E+07 | 7.02E+07 | 6.96E+07 |
|  | Rluc | 1.19E+09 | 1.12E+09 | 1.09E+09 |
| Relative Fluc/Rluc*100 | | 1.01E+02 | 1.06E+02 | 1.09E+02 |
| SLFN14 E211A-K213A | Fluc | 8.44E+07 | 8.36E+07 | 7.65E+07 |
|  | Rluc | 1.32E+09 | 1.31E+09 | 1.22E+09 |
| Relative Fluc/Rluc*100 | | 1.09E+02 | 1.08E+02 | 1.07E+02 |

| Exp3 |  | HIV-1(-1) | | |
| --- | --- | --- | --- | --- |
| pCDNA3.1(-) | Fluc | 7.60E+07 | 8.21E+07 | 8.25E+07 |
|  | Rluc | 8.58E+08 | 8.75E+08 | 8.81E+08 |
| Relative Fluc/Rluc*100 | | 9.63E+01 | 1.02E+02 | 1.02E+02 |
| SLFN14 | Fluc | 1.16E+07 | 1.20E+07 | 1.20E+07 |
|  | Rluc | 2.39E+08 | 2.32E+08 | 2.36E+08 |
| Relative Fluc/Rluc*100 | | 5.30E+01 | 5.61E+01 | 5.51E+01 |
| SLFN14 E211A | Fluc | 4.96E+07 | 4.64E+07 | 4.61E+07 |
|  | Rluc | 5.28E+08 | 5.06E+08 | 4.96E+08 |
| Relative Fluc/Rluc*100 | | 1.02E+02 | 9.97E+01 | 1.01E+02 |
| SLFN14 E211A-K213A | Fluc | 1.79E+07 | 1.89E+07 | 1.91E+07 |
|  | Rluc | 1.81E+08 | 1.87E+08 | 1.78E+08 |
| Relative Fluc/Rluc*100 | | 1.07E+02 | 1.10E+02 | 1.17E+02 |

Luciferase activities were measured with the Dual-Luciferase Reporter Assay System.

Fluc: Firefly luciferase; Rluc: Renilla luciferase.

# **Table S6. Synthesized genes in this study**

| **Gene names** | **genes sequences** |
| --- | --- |
| SLFN14 ^mut^ | **>**ATGGAGAGTCTCAAGACTGATACTGAAATGCCGTATCCTGAGGTAATAGTAGATGTGGGCAGAGTGATTTTTGGAGAAGAAAACAGGAAGAAGATGACCAACAGCTGTTTGAAAAGATCTGAGAATTCTAGAATTATCCGGGCTATATGTGCACTGTTAAATTCTGGAGGTGGTGTGATCAAAGCAGAGATTGATGATAAAACCTATAGTTACCAATGCCATGGGCTGGGACAGGATTTGGAAACTTCTTTTCAAAAGCTCCTTCCTTCAGGTTCACAGAAATACCTTGACTACATGCAGCAGGGGCACAATCTCCTGATTTTTGTGAAGTCATGGAGCCCAGATGTTTTCAGCCTTCCACTAAGGATTTGCAGCTTGCGCTCCAATTTGTATCGGAGAGATGTGACTTCTGCTATCAACTTGAGTGCTAGCAGTGCCCTGGAGCTTCTCAGAGAGAAGGGGTTTAGAGCCCAAAGAGGAAGACCAAGGGTGAAGAAGTTGCATCCTCAGCAGGTTCTCAATAGATGCATTCAGGAAGAGGAAGATATGAGGATATTGGCCTCAGAATTTTTTAAAAAGGACAAACTCATGTATAAGGAGAAACTCAACTTTACTGAGTCAACACATGTTGCTTTTAAAAGGTTCACCACCAAAAAAGTCATACCTCGGATTAAGGAAATGCTGCCTCATTATGTTTCTGCATTTGCCAACACTCAAGGGGGATATGTCCTCATTGGGGTGGATGATAAGAGCAAAGAAGTGGTTGGATGTAAGTGGGAAAAAGTGAATCCTGACTTACTAAAAAAAGAAATCGAAAACTGCATAGAAAAATTGCCTACATTCCACTTCTGCTGTGAGAAGCCAAAGGTAAATTTCACTACAAAAATCCTGAATGTGTACCAAAAAGATGTCCTGGATGGTTATGTCTGTGTGATTCAAGTGGAGCCCTTCTGTTGCGTGGTGTTTGCAGAGGCCCCAGATTCCTGGATCATGAAAGACAATTCTGTCACACGGCTGACAGCTGAGCAGTGGGTGGTCATGATGCTGGATACTCAGTCAGCTCCTCCCAGTTTGGTCACAGACTACAACTCTAGTCTGATTTCATCAGCTTCATCTGCACGAAAAAGTCCCGGATATCCCATAAAAGTCCACAAATTTAAGGAGGCTCTGCAACGACATTTGTTTCCAGTGACACAGGAAGAGGTACAATTTAAACCAGAATCCCTCTGTAAGAAGCTGTTCTCAGATCATAAAGAACTGGAGGGTTTAATGAAGACCCTGATACATCCTTGTTCTCAGGGGATTGTGATATTTTCTAGAAGCTGGGCTGGTGATGTTGGCTTCAGGAAAGAACAGAATGTCCTGTGTGATGCTCTCCTGATAGCAGTTAACAGCCCCGTGGTACTCTATACAATCTTAATAGACCCCAATTGGCCTGGAGGACTTGAATATGCCCGAAACACAGCTCATCAGTTAAAGCAGAAACTGCAAACTGTTGGTGGTTACACAGGGAAAGTGTGCATCATTCCAAGGCTGATACACCTGAGCAGCACACAGAGTAGACCTGGTGAGATCCCCCTGCGTTACCCCAGGTCCTACAGGCTTGCTGATGAGGAGGAAATGGAAGACTTGCTGCAGGCTCTGGTGGTGGTCTCCCTGTCCTCCAGATCTCTTCTGAGTGACCAGATGGGCTGTGAATTTTTCAACTTGCTCATAATGGAGCAGAGCCAGTTGCTTTCTGAGAGTCTTCAGAAGACACGTGAATTATTCATCTACTGCTTTCCAGGAGTCAGGAAGACAGCCCTAGCCATAAAGATCATGGAGAAAATTAAGGACTTGTTTCACTGCAAACCAAAAGAGATCCTCTATGTTTGTGAAAGCGACTCCCTAAAGGATTTTGTGACCCAACAAACCACCTGCCAAGCTGTGACCAGGAAAACTTTCATGCAAGGGGAGTTTCTAAAGATTAAACACATAGTGATGGATGAGACTGAGAATTTCTGCAGCAAATATGGCAATTGGTACATGAAGGCTAAGAACATCACCCATCCAAAGGCGAAGGGGACTGGAAGTGAAAACCTTCACCATGGGATTCTCTGGCTTTTTCTTGACCCTTTTCAAATCCATCACGCAGATGTCAATGGCCTTCCCCCTCCATCTGCTCAGTTTCCTCGAAAAACAATCACCAGTGGGATCCACTGTGCTCTGGAAATAGCGAAGGTTATGAAAGAAGAAATGAAGAGGATCAAAGAAAATCCTCCCTCCAACATGTCTCCAGACACATTAGCATTGTTCAGCGAGACTGCCTATGAGGAAGCAACGAGCGCCCAGGCTCTGCCTGGGGTGTGTGAGACAAAGACTAATCTGACAACAGAACAAATAGCTAACTATGTGGCAAGAAAATGTCACAGCCTGTTCCAGAGCGGCTATCTGCCCAAAGATATAGCAATTCTGTGCAGGAGAGGGGAGGACAGAGGACGCTATAGGCTTGCACTACTCAAAGCAATGGAATTAATTGAGACCCACAGACCATCAGAGGTTGTGTTTAGCCCGGCCACCGGTGTTTGGGGAAGTCACATTGTTTTAGACAGTATTCAGCAATTTTCAGGCCTGGAGAGGACTGTCGTGTTTGGGCTTAGTCCAGAAAGCGACCAGTCAGAGGAATTTCATAAGCTCTGCTTTGCTTCAAGAGCCATTAAACACCTCTACCTGCTTTATGAAAAGAGGGCAGCCTACTGA |
| WT-SLFN14 | **>**ATGGAGAGTCTAAAGACGGATACCGAGATGCCGTACCCGGAGGTGATTGTGGATGTGGGCCGCGTGATCTTCGGAGAGGAGAATCGAAAGAAGATGACCAACTCCTGCCTGAAACGCTCCGAAAACTCCCGCATCATCAGAGCGATCTGCGCCCTGCTGAACTCGGGAGGCGGCGTGATCAAGGCCGAGATCGATGATAAGACCTACAGCTACCAGTGCCACGGACTGGGCCAGGATCTGGAGACGAGCTTCCAGAAGCTGCTGCCCAGCGGCTCGCAGAAGTACCTGGATTACATGCAGCAGGGCCATAACCTGCTGATCTTCGTGAAGAGCTGGAGTCCTGACGTGTTCTCCCTGCCCCTGCGCATTTGCAGCCTGCGCTCGAACCTGTATCGTCGTGATGTGACTTCCGCCATCAACCTGTCCGCCAGCAGCGCCCTGGAGCTGCTGCGCGAAAAGGGCTTCCGCGCCCAGAGGGGCCGCCCCCGCGTGAAGAAGCTGCACCCACAGCAGGTGCTGAACCGCTGTATTCAGGAGGAGGAGGATATGCGTATCCTGGCCTCGGAGTTCTTCAAAAAGGATAAGCTGATGTACAAGGAGAAGCTGAATTTCACCGAGAGCACACACGTTGAGTTCAAGCGCTTCACCACGAAGAAGGTGATCCCCCGCATTAAGGAGATGCTGCCCCATTATGTGTCGGCCTTCGCCAACACCCAGGGCGGCTACGTGCTGATCGGCGTGGACGACAAGTCCAAGGAGGTGGTGGGCTGCAAGTGGGAGAAGGTGAATCCCGATCTGCTGAAGAAGGAGATCGAGAACTGCATCGAGAAGCTGCCCACCTTCCACTTCTGCTGCGAGAAGCCAAAGGTGAACTTCACCACGAAGATTCTGAACGTTTACCAGAAGGATGTGCTGGATGGCTACGTGTGCGTGATCCAGGTGGAGCCGTTCTGCTGCGTGGTGTTCGCCGAGGCCCCGGATAGCTGGATCATGAAGGATAACTCCGTCACCCGCCTGACCGCCGAGCAGTGGGTGGTGATGATGCTGGATACCCAGTCCGCCCCACCGTCGCTGGTGACGGATTACAATAGCTGCCTGATCAGCTCCGCTTCTTCCGCCCGCAAGTCCCCAGGCTACCCGATCAAGGTGCATAAGTTCAAGGAGGCTCTGCAGCGGCATCTGTTCCCCGTCACCCAGGAGGAGGTTCAGTTCAAGCCGGAGTCCCTGTGCAAGAAGCTGTTCTCCGATCACAAGGAGCTGGAGGGCCTGATGAAGACCCTGATCCACCCGTGCAGCCAGGGCATTGTGATCTTCAGCCGCTCGTGGGCCGGCGACGTGGGCTTCCGCAAGGAGCAGAATGTGCTGTGCGACGCCCTGCTGATCGCCGTCAACAGCCCAGTCGTGCTGTACACCATACTGATCGATCCGAACTGGCCAGGCGGACTGGAGTACGCTCGCAACACGGCCCACCAGCTGAAGCAGAAGCTGCAGACCGTGGGCGGCTATACGGGCAAGGTGTGCATCATCCCGCGCCTGATCCATCTGTCGAGCACGCAGTCGCGCCCCGGAGAGATCCCGCTGCGCTACCCCCGAAGTTACCGCCTGGCCGATGAGGAGGAGATGGAGGATCTGCTGCAGGCCCTGGTGGTGGTGTCGCTCTCCTCGCGCAGCCTGCTGTCCGATCAGATGGGATGCGAGTTTTTCAACCTGCTGATCATGGAGCAGTCCCAACTGCTGTCTGAATCGCTGCAGAAGACCCGCGAGCTGTTCATTTACTGCTTCCCAGGAGTGCGCAAGACGGCTCTGGCCATCAAAATCATGGAGAAGATCAAGGATCTGTTTCACTGCAAGCCCAAGGAGATCCTGTACGTCTGCGAGAGCGATTCCCTGAAGGATTTTGTGACCCAGCAGACAACCTGCCAGGCCGTGACCCGCAAGACCTTCATGCAGGGTGAGTTCCTAAAGATAAAGCACATCGTGATGGACGAGACCGAGAATTTCTGCAGCAAGTACGGAAACTGGTACATGAAAGCCAAAAACATCACCCACCCCAAGGCCAAGGGCACCGGCTCCGAGAACCTGCACCACGGCATCCTGTGGCTGTTTCTGGACCCGTTCCAGATCCACCATGCGGACGTGAACGGCCTGCCCCCGCCCTCCGCCCAGTTCCCCCGCAAGACTATCACCAGCGGCATCCACTGCGCCCTGGAGATCGCCAAGGTGATGAAAGAGGAAATGAAGCGCATCAAGGAGAACCCCCCCTCGAATATGAGCCCGGATACCCTGGCCCTGTTCAGCGAGACCGCTTATGAGGAGGCCACGTGCGCCCAGGCCCTGCCCGGCGTGTGCGAGACCAAGACCAACCTGACCACCGAGCAGATCGCCAATTACGTGGCCCGCAAGTGCCACTCGCTGTTCCAGTGTGGCTACTTGCCCAAGGACATCGCCATCCTGTGCCGCCGGGGAGAGGACCGCGGACGCTACCGCCTGGCCTTGCTGAAGGCCATGGAGCTGATTGAGACCCACCGCCCGAGCGAGGTGGTCTTTAGCCCGGCCACCGGCGTCTGGGGAAGCCACATCGTGCTGGACTCCATTCAGCAGTTCAGCGGCCTGGAGCGAACCGTGGTGTTTGGACTGAGCCCCGAGTGCGATCAGAGCGAGGAGTTCCACAAGCTGTGTTTTGCCTCTCGCGCCATCAAACACCTCTACCTGCTGTACGAGAAACGCGCCGCCTAC |

# **Table S7. Primers for the construction of recombinant plasmids**

| Name | Forward primer (5’-3’) | Reverse primer (5’-3’) |
| --- | --- | --- |
| pFastBac I-6xHis-SUMO-SLFN14^mut^ | CAGGGGCCCCTGGGATCCATGGAGAGTCTCAAG | GGTACCGCATGCCTCGAGTCAGTAGGCTGCCCTC |
| pCDNA3.1-strepII-SUMO-SLFN14 | tagtccagtgtggtggaattcATGGCCACCTggtcccacccccagttcgagaagtggagccacccccagttcgaaaagGGGTCCCTGCAGGACTCAGA | gaagggccctctagactcgagCTAGTAGGCGGCGCGTTTC |
| pCDNA3.1-strepII-SUMO- SLFN14 E211A | CACACGTTGCATTCAAGCGCTTCACCAC | CGCTTGAATGCAACGTGTGTGCTCTCGG |
| pCDNA3.1-strepII-SUMO- SLFN14 K213A | GTTGAGTTCGCCCGCTTCACCACGAAGAA | GTGAAGCGGGCGAACTCAACGTGTGTGC |
| pCDNA3.1-strepII-SUMO- SLFN14 R214A | GTTCAAGGCCTTCACCACGAAGAAGGT | GTGGTGAAGGCCTTGAACTCAACGTGTG |
| pCDNA3.1-strepII-SUMO- SLFN14 F215D | CAAGCGCGACACCACGAAGAAGGTGAT | CTTCGTGGTGTCGCGCTTGAACTCAACGT |
| pCDNA3.1-strepII-SUMO- SLFN14 T216D | GCGCTTCGACACGAAGAAGGTGATCCC | CTTCTTCGTGTCGAAGCGCTTGAACTCAAC |
| pCDNA3.1-strepII-SUMO- SLFN14 T217D | CTTCACCGACAAGAAGGTGATCCCCCG | CCTTCTTGTCGGTGAAGCGCTTGAACTC |
| pCDNA3.1-strepII-SUMO- SLFN14 K218A | CTTCACCACGGCCAAGGTGATCCCCCGCAT | GATCACCTTGGCCGTGGTGAAGCGCTTGA |
| pCDNA3.1-strepII-SUMO- SLFN14 K219A | CACGAAGGCCGTGATCCCCCGCATTAA | GGATCACGGCCTTCGTGGTGAAGCGCT |
| pCDNA3.1-strepII-SUMO- SLFN14 R223A | GATCCCCGCCATTAAGGAGATGCTGCC | CCTTAATGGCGGGGATCACCTTCTTCG |
| pCDNA3.1-strepII-SUMO- SLFN14 M227D | CATTAAGGAGGACCTGCCCCATTATGTGTC | GGGGCAGGTCCTCCTTAATGCGGGGGA |
| pCDNA3.1-strepII-SUMO- SLFN14 S35D | GACCAACGACTGCCTGAAACGCTCCGA | TCAGGCAGTCGTTGGTCATCTTCTTTC |
| pCDNA3.1-strepII-SUMO-PSP SLFN14 K38A | CTGCCTGGCCCGCTCCGAAAACTCCCG | CGGAGCGGGCCAGGCAGGAGTTGGTCA |
| pCDNA3.1-strepII-SUMO- SLFN14 R39A | CCTGAAAGCCTCCGAAAACTCCCGCAT | GTTTTCGGAGGCTTTCAGGCAGGAGTTGG |
| pCDNA3.1-strepII-SUMO- SLFN14 Q78D | GACTGGGCGACGATCTGGAGACGAGCTT | CCAGATCGTCGCCCAGTCCGTGGCACTG |
| pCDNA3.1-strepII-SUMO- SLFN14 K86A | CTTCCAGGCCCTGCTGCCCAGCGGCTC | GCAGCAGGGCCTGGAAGCTCGTCTCCAG |
| pCDNA3.1-SLFN14-V5 | tagtccagtgtggtggaattcGCCACCATGGAGAGTCTAAAGACG | gaagggccctctagactcgagCTACGTAGAATCGAGACCGAGGAGAGGGTTAGGGATAGGCTTACCGTAGGCGGCGCGTTTCTC |
| pCDNA3.1-SLFN14  1-353-V5 | tagtccagtgtggtggaattcGCCACCATGGAGAGTCTAAAGACG | gaagggccctctagactcgagCTACGTAGAATCGAGACCGAGGAGAGGGTTAGGGATAGGCTTACCGGACTGGGTATCCAGCATCATC |
| pCDNA3.1-SLFN14 E211A-V5 | CACACGTTGCATTCAAGCGCTTCACCAC | CGCTTGAATGCAACGTGTGTGCTCTCGG |
| pCDNA3.1-SLFN14-E211A-K213A -V5 | GTTgcattcgccCGCTTCACCACGAAGAAGGT | AAGCGggcgaatgcAACGTGTGTGCTCTCGGTGAA |
| pCDNA3.1-SLFN14  354-End-V5 | tagtccagtgtggtggaattcGCCACCATGGCCCCACCGTCGCTGGT | gaagggccctctagactcgagCTACGTAGAATCGAGACCGAGGA |
| pDual-HIV( 0) | GAGAGACAGGCTAATTaTTTTAGGGAAGATCTGGCCTTCC | tAATTAGCCTGTCTCTCAGTGCTTCCGCCGCT |

# **Table S8. Sequences for the nucleic acid substrates**

| **Nucleic acids** | **Sequences** | **Description** |
| --- | --- | --- |
| ssRNA | 5’-UUUUUUUUUUUUUUUUUUUUU-3’ | 21nt, 5’Cy3 |
| G4RNA | 5’-CAACAACAACAAGGGAGGGGCGGGUCU  GGG-3’ | 30nt, 5’Cy3 |
| dsRNA | Sense: 5’-CUGAGGUAGUAGGUUGUAUCGCU  UUAGGGUCACACCCACCA-3’  Anti-sense:5’-UGGUGGGUGUGACCCUAAAGC  GAUACAACCUACUACCUCAUU-3’ | 40nt, Sense 5’Cy3 |
| hairpin RNA for endonuclease assays | 5’-GCAUGGCGGCCAUCAGCUACCUAAAGU  UCAGGUAGCUGAUGGCCGCCAUG-3’ | 50nt, 5’Cy3 |
| ssDNA | 5’-TTTTTTTTTTTTTTTTTTTTT-3’ | 21nt, 5’Cy3 |
| dsDNA | Sense: 5’-CTGAGGTAGTAGGTTGTATCGCTTT  AGGGTCACACCCACCA-3’  Anti-sense:5’-TGGTGGGTGTGACCCTAAAGC  GATACAACCTACTACCTCATT-3’ | 40nt, Sense 5’Cy3 |
| hairpin DNA | 5’-GCATGGCGGCCATCAGCTACCTAAAGTTC  AGGTAGCTGATGGCCGCCATG-3’ | 50nt, 5’Cy3 |
| Short hairpin RNA | 5’-GCAUCAGCUACCUAAAGUUCAGGUAGC  UGAUG-3’ | 32nt |
| Trap RNA | 5’-CGAAGCUGCUAACAUCAG-3’ | 18nt |
| RNA duplex with 5’-overhang | Sense: 5’-UUUUUUUUUUCUGAUGUUAGCA  GCUUCG-3’  Anti-sense: 5’-CGAAGCUGCUAACAUCAG-3’ | Anti-sense:18nt, 5’Cy5.5 |
| tRNA-Leu UAA | 5’-ACCAGGAUGGCCGAGUGGUUAAGGCG  UUGGACUUAAGAUCCAAUGGACAUAUGUCCGCGUGGGUUCGAACCCCACUCCUGGUACCA-3’ | 86nt, 5’Cy3 |
| tRNA-Leu AAG | 5’-GGUAGCGUGGCCGAGCGGUCUAAGGCG  CUGGAUUAAGGCUCCAGUCUCUUCGGAGGCGUGGGUUCGAAUCCCACCGCUGCCA-3’ | 82nt, 5’Cy3 |
| tRNA-Leu CAA | 5’-GUCAGGAUGGCCGAGUGGUCUAAGGCG  CCAGACUCAAGUUCUGGUCUCCGUAUGGAGGCGUGGGUUCGAAUCCCACUUCUGACACCA-3’ | 87nt, 5’Cy3 |
| tRNA-Leu CAG | 5’-GUCAGGAUGGCCGAGCGGUCUAAGGC  GCUGCGUUCAGGUCGCAGUCUCCCCUGGAGGCGUGGGUUCGAAUCCCACUCCUGACACCA-3’ | 86nt, 5’Cy3 |
| tRNA-Ser UGA | 5’-GUAGUCGUGGCCGAGUGGUUAAGGCG  AUGGACUUGAAAUCCAUUGGGGUUUCCCCGCGCAGGUUCGAAUCCUGCCGACUACGCCA-3’ | 85nt, 5’Cy3 |
| tRNA-Ser AGA | 5’-GUAGUCGUGGCCGAGUGGUUAAGGCG  AUGGACUAGAAAUCCAUUGGGGUUUCCCCGCGCAGGUUCGAAUCCUGCCGACUACGCCA-3’ | 85nt, 5’Cy3 |
| tRNA-Ser CGA | 5’-GCUGUGAUGGCCGAGUGGUUAAGGCG  UUGGACUCGAAAUCCAAUGGGGUCUCCCCGCGCAGGUUCGAAUCCUGCUCACAGCGCCA-3’ | 85nt, 5’Cy3 |
| tRNA-Ser GCU | 5’-GACGAGGUGGCCGAGUGGUUAAGGCG  AUGGACUGCUAAUCCAUUGUGCUCUGCACGCGUGGGUUCGAAUCCCACCCUCGUCGCCA-3’ | 85nt, 5’Cy3 |
| tRNA-His GUG | 5’-GGCCGUGAUCGUAUAGUGGUUAGUACU  CUGCGUUGUGGCCGCAGCAACCUCGGUUCGAAUCCGAGUCACGGCACCA-3’ | 76nt, 5’Cy3 |
| tRNA-Sec UCA | 5’-GCCCGGAUGAUCCUCAGUGGUCUGGG  GUGCAGGCUUCAAACCUGUAGCUGUCUAGCGACAGAGUGGUUCAAUUCCACCUUUCGGGCGCCA-3’ | 90nt, 5’Cy3 |
| -PRF | 5’-GACACCAAATGAAAGATTGTACTGAGAG  ACAGGCTAATTTTTTAGGGAAGATCTGGCCTTCCTACAAGGGAAGGCCAGGGAATTTTCTTCAGAGCAGACC-3’ | 100nt, |
| PRF | 5’-AATTTTTTAGGGAAGATCTGGCCTTCCTA  CAAGGGAAGGCCAGGGAATTTTCTTC-3’ | 55nt, 5’Cy5.5 |

**Fig. 5b**

**
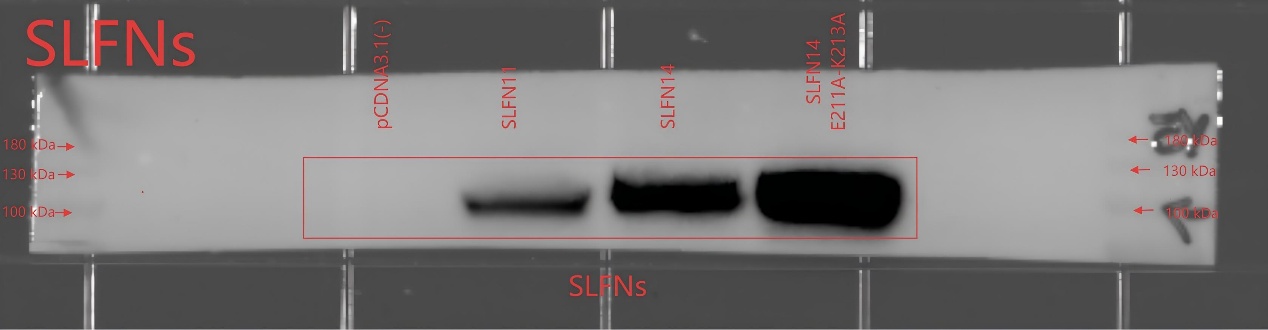
**

**
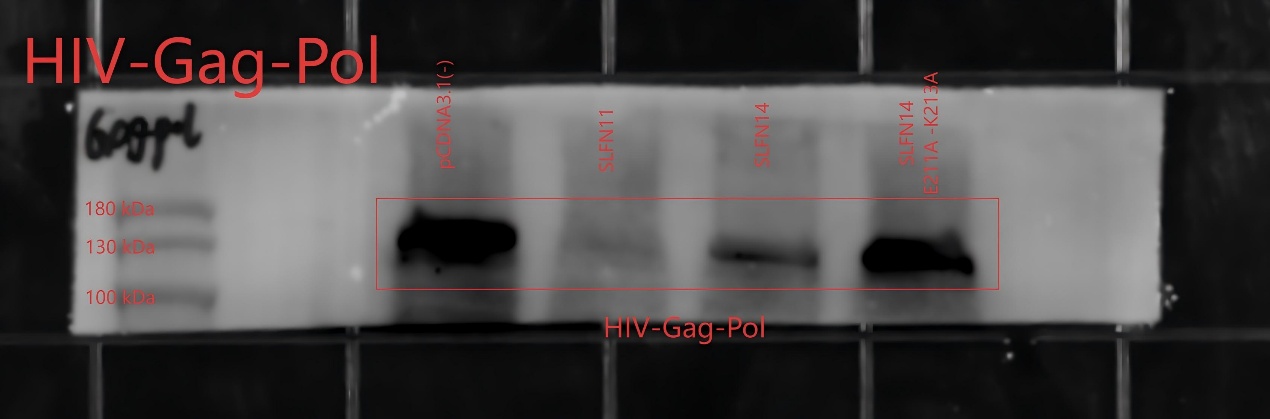
**

**
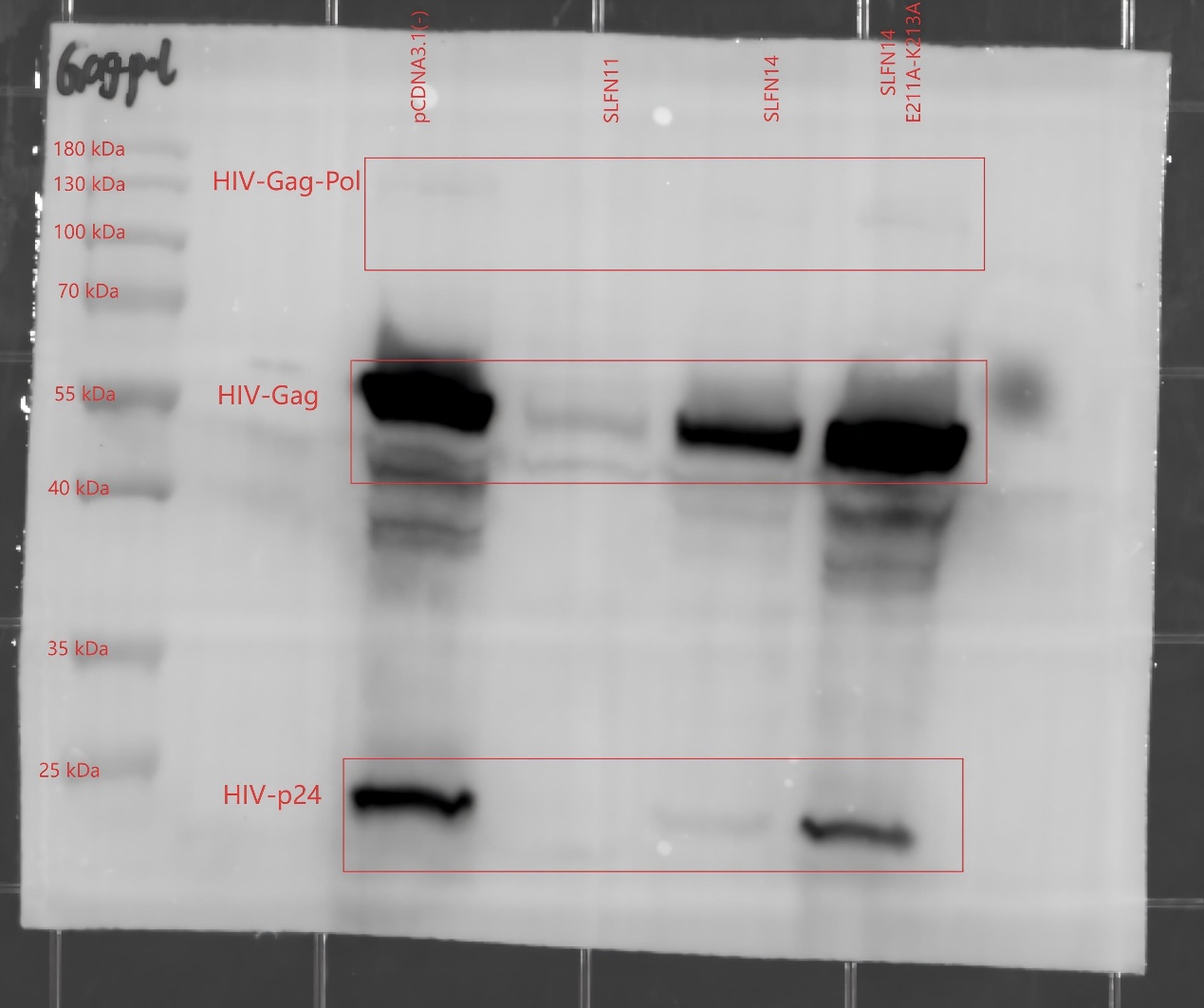
**

**
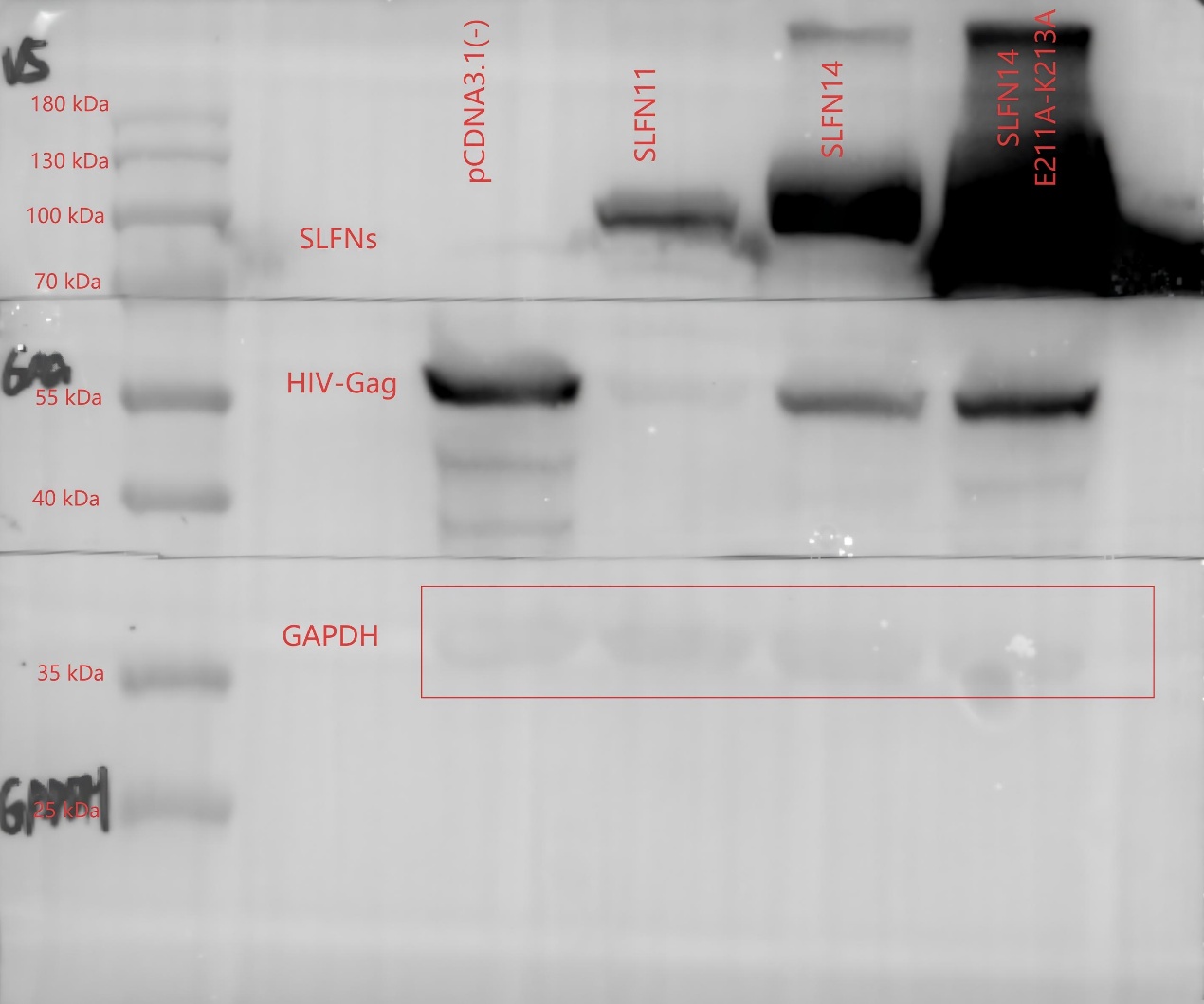
**

**Fig.5 c**

**
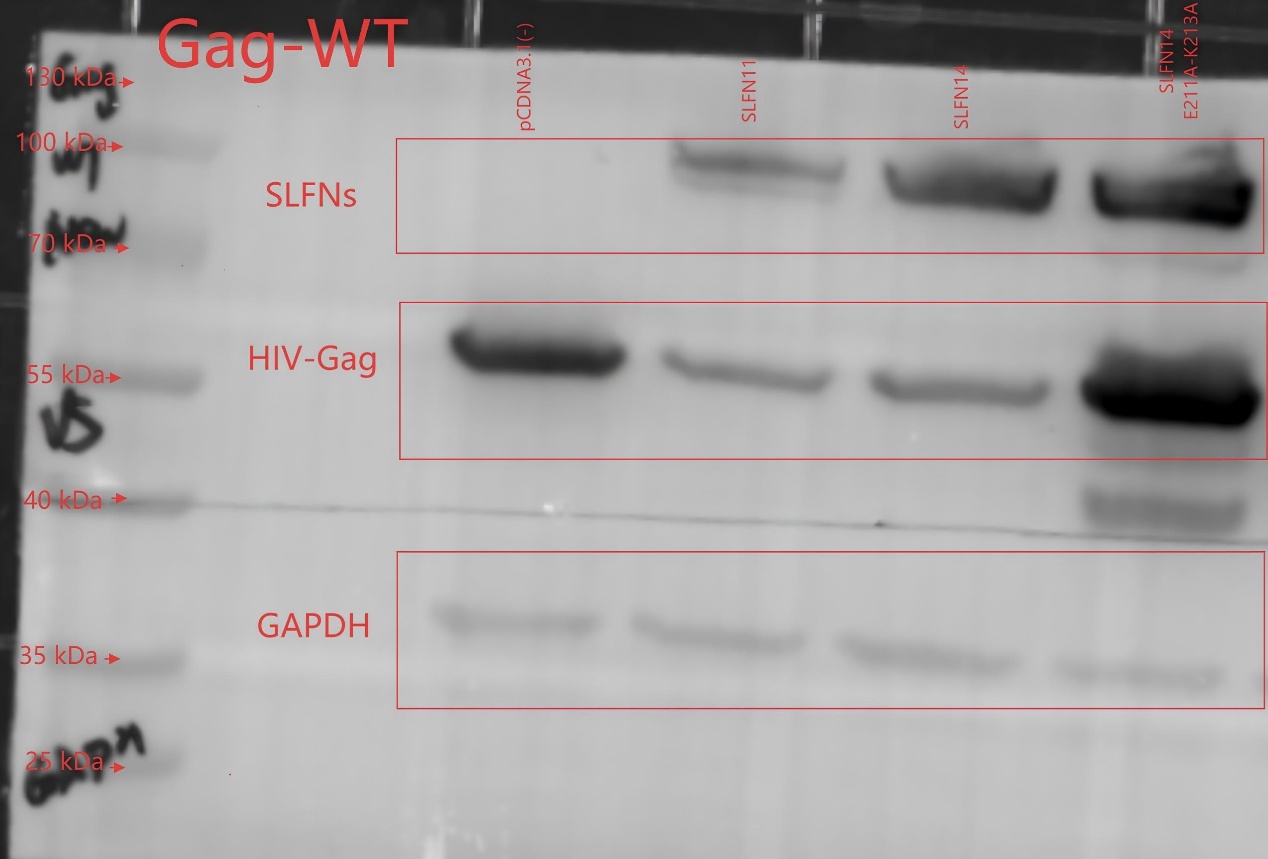
**

**
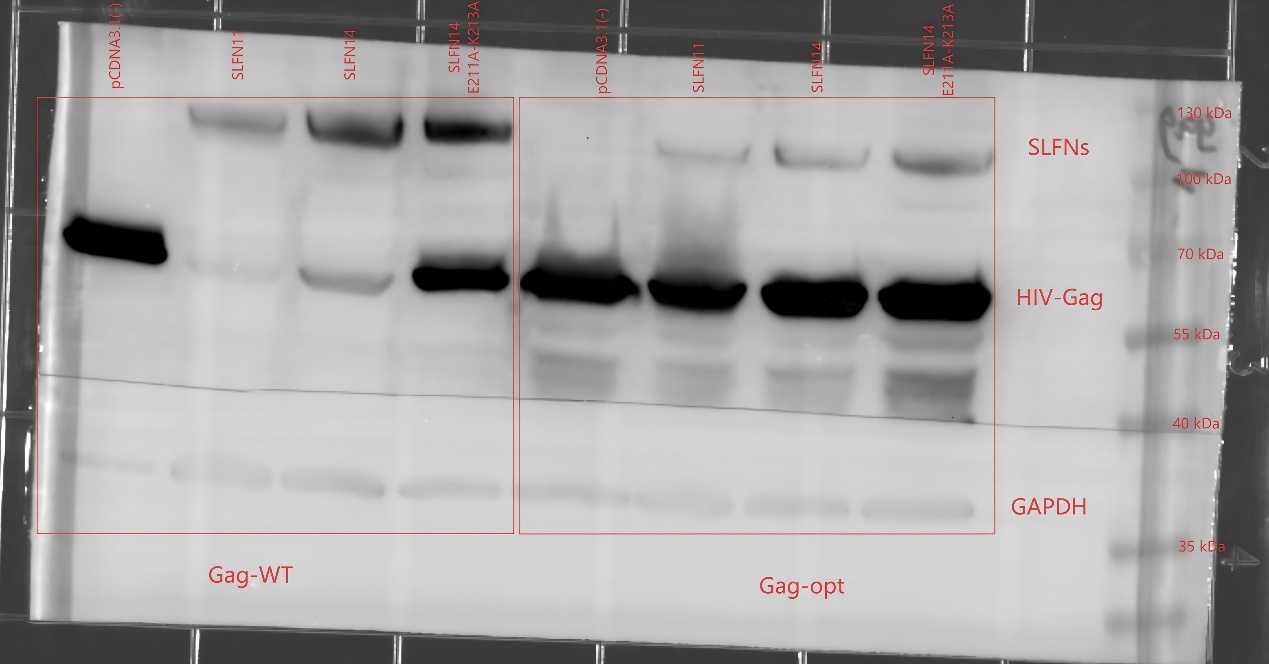
**

**
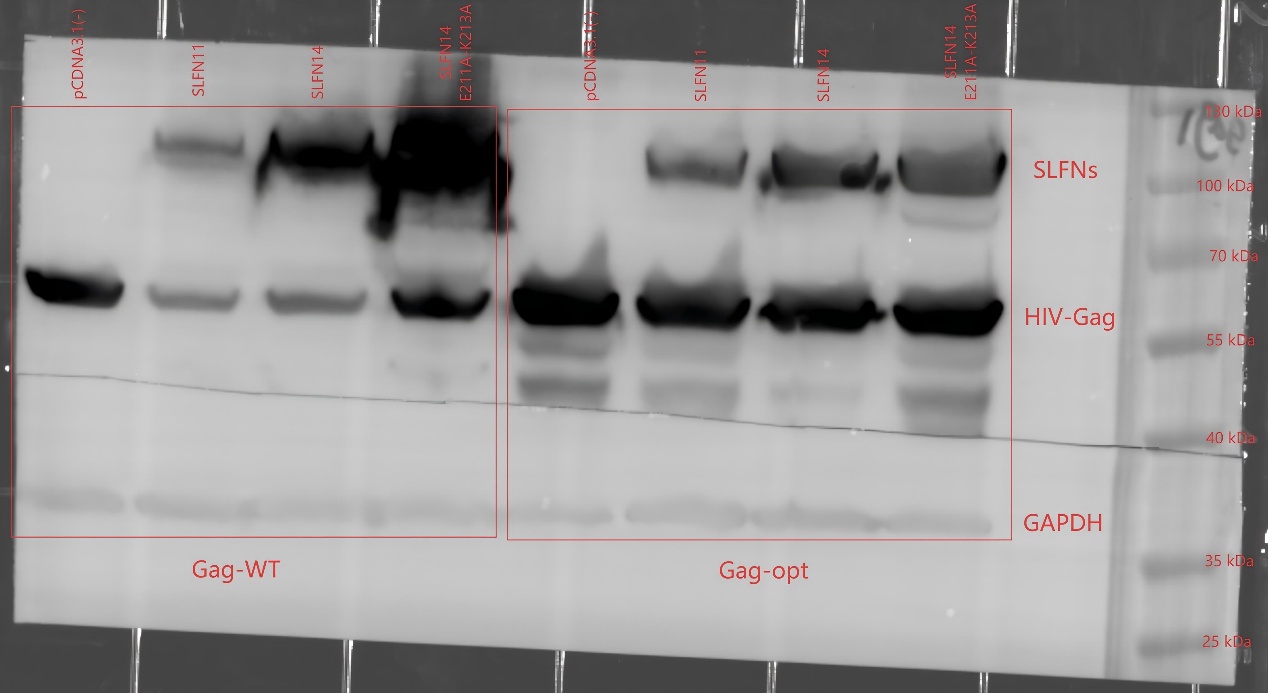
**

**Fig.5 f**

**
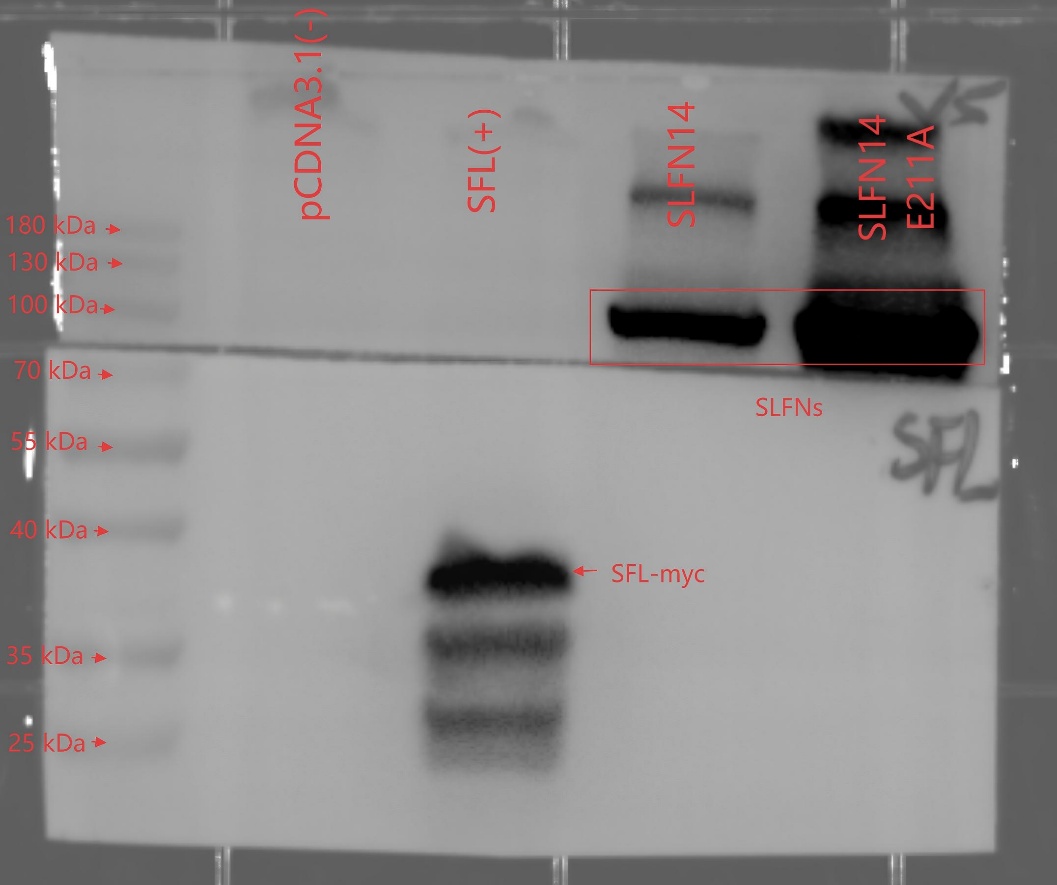
**

**
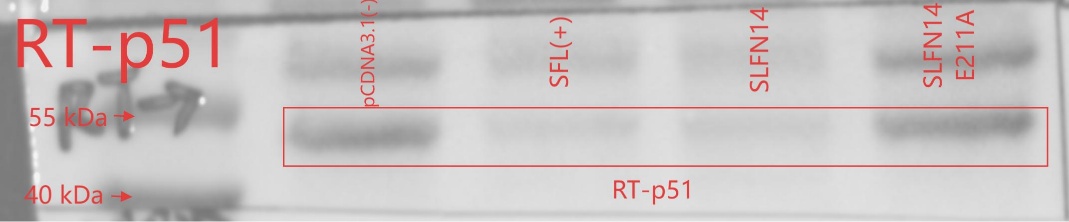
**

**
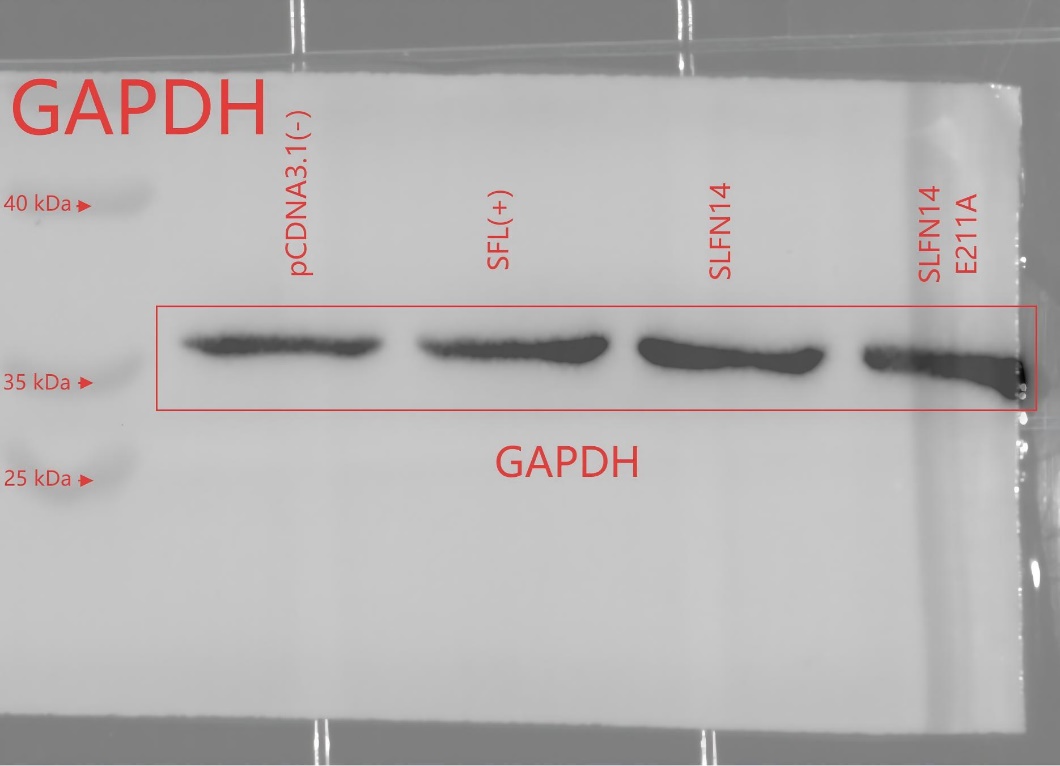
**

**Fig.6 e**

**
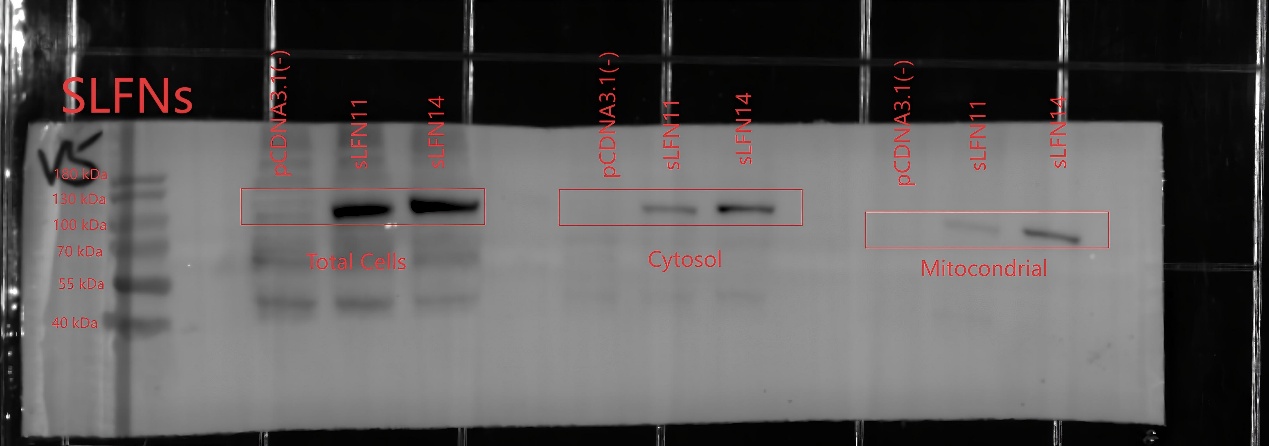
**

**
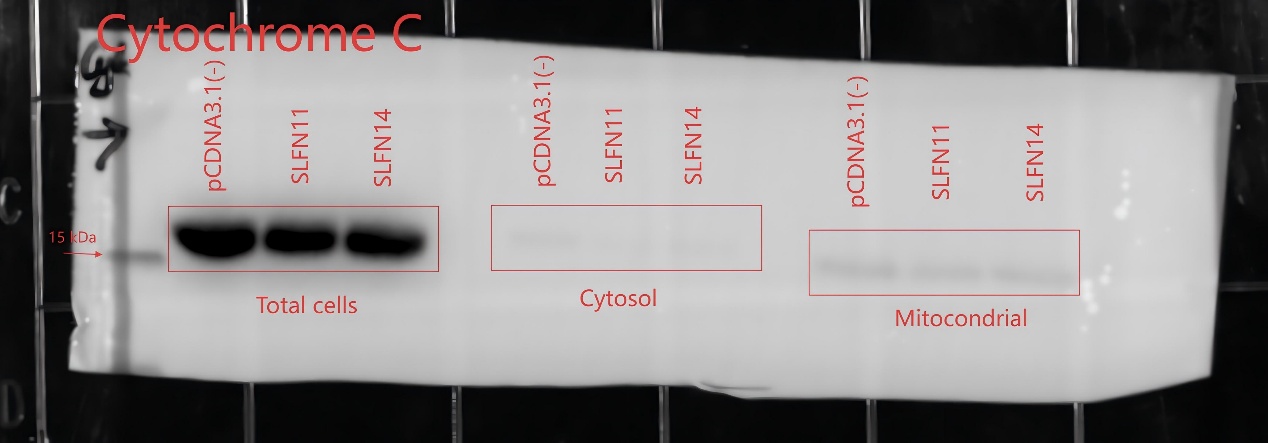
**

**
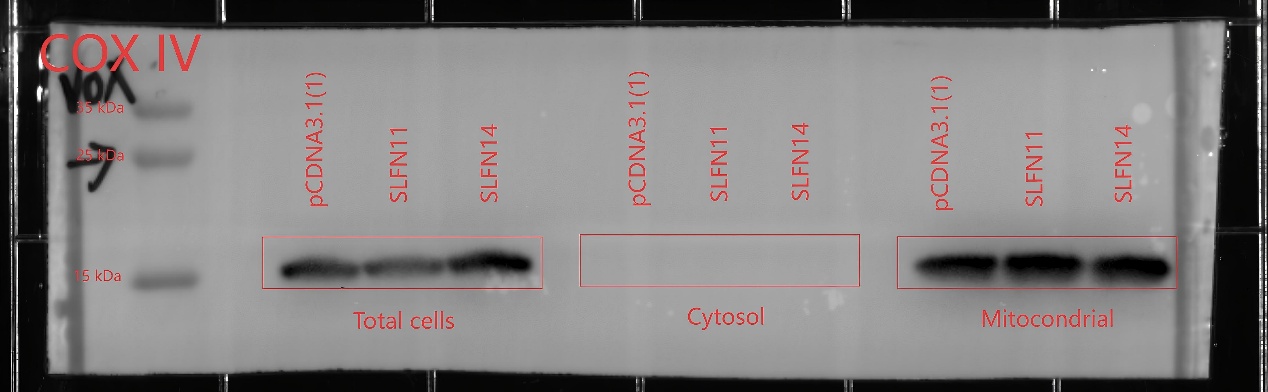
**

**
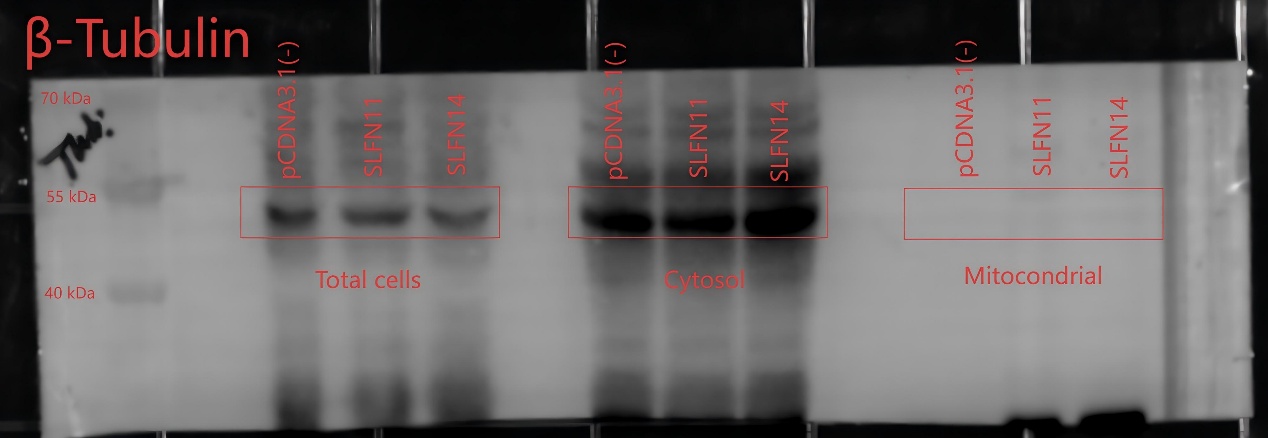
**
